# School-based interventions to prevent anxiety and depression in children and young people: a systematic review and network meta-analysis

**DOI:** 10.1016/S2215-0366(19)30403-1

**Published:** 2019-12

**Authors:** Deborah M Caldwell, Sarah R Davies, Sarah E Hetrick, Jennifer C Palmer, Paola Caro, José A López-López, David Gunnell, Judi Kidger, James Thomas, Clare French, Emily Stockings, Rona Campbell, Nicky J Welton

**Affiliations:** a Population Health Sciences, Bristol Medical School, University of Bristol, Bristol, UK; b School for Policy Studies, University of Bristol, Bristol, UK; c Departamento de Psicología Básica y Metodología, Facultad de Psicología, Universidad de Murcia, Spain; d National Drug and Alcohol Research Centre, University of New South Wales, Sydney, NSW, Australia; e EPPI-Centre, University College, London, UK; f Faculty of Medical and Health Sciences, University of Auckland, Auckland, New Zealand; g NIHR Biomedical Research Centre at University Hospitals Bristol NHS Foundation Trust, Bristol, UK

## Abstract

**Background:**

Rates of anxiety and depression are increasing among children and young people. Recent policies have focused on primary prevention of mental disorders in children and young people, with schools at the forefront of implementation. There is limited information for the comparative effectiveness of the multiple interventions available.

**Methods:**

We did a systematic review and network meta-analysis, searching MEDLINE, Embase, PsycINFO, and Cochrane Central Register of Controlled trials for published and unpublished, passive and active-controlled randomised and quasi-randomised trials. We included educational setting-based, universal, or targeted interventions in which the primary aim was the prevention of anxiety and depression in children and young people aged 4–18 years. Primary outcomes were post-intervention self-report anxiety and depression, wellbeing, suicidal ideation, or self-harm. We assessed risk of bias following the Cochrane Handbook for Systematic Reviews of Interventions. We estimated standardised mean differences (SMD) using random effects network meta-analysis in a Bayesian framework. The study is registered with PROPSERO, number CRD42016048184.

**Findings:**

1512 full-text articles were independently screened for inclusion by two reviewers, from which 137 studies of 56 620 participants were included. 20 studies were assessed as being at low risk of bias for both random sequence generation and allocation concealment. There was weak evidence to suggest that cognitive behavioural interventions might reduce anxiety in primary and secondary settings. In universal secondary settings, mindfulness and relaxation-based interventions showed a reduction in anxiety symptoms relative to usual curriculum (SMD −0·65, 95% credible interval −1·14 to −0·19). There was a lack of evidence to support any one type of intervention being effective to prevent depression in universal or targeted primary or secondary settings. Comparison-adjusted funnel plots suggest the presence of small-study effects for the universal secondary anxiety analysis. Network meta-analysis was not feasible for wellbeing or suicidal ideation or self-harm outcomes, and results are reported narratively.

**Interpretation:**

Considering unclear risk of bias and probable small study effects for anxiety, we conclude there is little evidence that educational setting-based interventions focused solely on the prevention of depression or anxiety are effective. Future research could consider multilevel, systems-based interventions as an alternative to the downstream interventions considered here.

**Funding:**

UK National Institute for Health Research.

## Introduction

Common mental disorders are a key cause of morbidity in children and young people younger than 18 years. Globally, depressive disorders are the third most frequent cause of adolescent disability-adjusted life-years lost, and anxiety disorders are the fifth most frequent cause of disability-adjusted life-years lost for adolescent girls.^1^ Evidence suggests that lifetime trajectories of common mental disorders are established by mid-adolescence^2^ with half of all mental disorders starting by age 14 years, and three-quarters by age 25 years.^3^ Even in the context of under-reporting and detection, rates of anxiety and depression are high and increasing among children and young people.^4^, ^5^, ^6^

The use of psychopharmacotherapy for children and young people is debated^7^, ^8^, ^9^, ^10^ and the capacity of child and adolescent mental health services remains under-resourced worldwide.^11^ Economic modelling studies suggest that, even with optimal health-care access and treatment, less than 30% of the burden of common mental disorders could be alleviated.^12^ Against this background there is a growing imperative for primary prevention of common mental disorders in children and young people.

Globally, educational settings are at the forefront of the prevention initiative.^13^ In the UK, the Green Paper, Transforming Children and Young People's Mental Health Provision,^14^ calls for every school to appoint a mental health lead and for a greater role for schools in cross-sectoral mental health teams. Multiple systematic reviews of preventive interventions^15^, ^16^, ^17^, ^18^, ^19^ suggest a modest beneficial effect for prevention of depression and anxiety in the short term. However, high levels of statistical heterogeneity are common, rendering policy interpretation and implementation challenging.^20^ Heterogeneity can be caused by combining studies over disparate populations, settings, or interventions.^21^ In standard pairwise meta-analysis only two interventions can be compared at a time (eg, mental health interventions versus control). This makes it necessary to conflate disparate interventions together to form a single comparator that is compared with a conflated control condition. Consequently, standard meta-analyses can only help a policy maker understand if an intervention works in principle but do not provide evidence for the comparative effectiveness of specific competing intervention options. The need to conflate interventions and controls can be mitigated by using a network meta-analysis. Network meta-analyses combine all available direct and indirect evidence on relative intervention effects in a single coherent analysis, which can increase the precision of effect estimates.^22^ This type of analysis also allows for more detailed definitions of each intervention to be specified and for heterogeneity to be minimised.^23^ Network meta-analysis enables the ranking of interventions according to the probability that each is the best, or worst, for a given outcome, to help inform policy decisions.

**Research in context**

**Evidence before this study**

Previous systematic reviews of educational setting-based preventive interventions suggested a modest effect for anxiety and depression. However, the comparative effectiveness of the multiple competing interventions available has not been assessed. We did a network meta-analysis to identify if any intervention could be considered superior for preventing anxiety and depression in children and young people.

We searched MEDLINE, Embase, PsycINFO and Cochrane Central Register of Controlled trials from inception to April 4, 2018. Full search strategies are reported in the supplementary information. Studies were eligible for inclusion if they were randomised or quasi-randomised trials of educational setting-based, universal or targeted, interventions explicitly for the prevention of anxiety and depression in children and young people aged 4–18 years. Psychological, psychosocial, educational, or physical interventions implemented in educational settings were included. We did not restrict by language.

**Added value of this study**

To the best of our knowledge, this is the first network meta-analysis of interventions to prevent anxiety and depression in children and young people. It is the largest contemporary systematic review of preventive interventions in educational settings, including 137 studies of more than 56 000 participants. Our network meta-analysis retains the distinct identity of every intervention and control comparator enabling us to rank the interventions according the probability they were most effective. Our findings contradict previously published reviews, as we observed little evidence to suggest that school-based interventions are effective for prevention of anxiety or depression. Most studies were at unclear risk of bias for random sequence generation and allocation concealment and there was evidence of small study effects for self-report anxiety outcomes. In a post-hoc analysis, our results were consistent with previous reviews when we combined the four distinct control conditions to form a single comparator.

**Implications of available evidence**

There is insufficient evidence to recommend school-based anxiety and depression prevention interventions. We conclude that the beneficial effect observed in previous meta-analyses is possibly due to conflating control conditions. Future trials should be commissioned only if they use an active-control condition, such as an attention control or an alternative intervention. The results reported here are part of a larger study of effectiveness and cost-effectiveness of components of preventive anxiety and depression interventions in educational settings (NIHR 15/49/08).

Network meta-analysis is routinely used for health technology assessments and by health reimbursement agencies worldwide.^24^ We report a systematic review and network meta-analysis to assess the comparative effectiveness of educational setting-based interventions for preventing depression and anxiety in children and young people. Although network meta-analysis has previously been applied to public health interventions,^25^ to our knowledge this is the first of preventive mental health interventions comparing effectiveness of distinct psychological, educational, and physical interventions in a single analysis for children and young people.

## Methods

### Search strategy and selection criteria

We searched MEDLINE, Embase, PsycINFO, and the Cochrane Central Register of Controlled Trials (appendix p 2), from the earliest date possible until April 4, 2018. Searches were not restricted by language, country, or date of publication. We also searched Epistemonikos.org to identify relevant published systematic reviews and imported all references into our database for eligibility assessment.

Randomised and quasi-randomised controlled trials were eligible, where quasi-randomised was defined as based on a pseudo-random sequence (eg, date of birth). We included both individually randomised and cluster-randomised trials. Eligible trials included participants aged 4–18 years at recruitment and in full or part-time education. Studies were eligible if they included psychological, psychosocial, educational, or physical interventions that were implemented in educational settings to children and young people as individuals or in groups. Universal or targeted (selective or indicated) interventions were eligible if the explicit aim was to prevent anxiety and depression. We adopted the Institute of Medicine (now officially known as National Academy of Medicine) definitions of primary prevention that refer to universal, selective, and indicated prevention,^26^ which is to say that universal prevention addresses whole populations not defined on the basis of risk; selective prevention is targeted at subgroups with higher than average risk of developing a mental disorder; and indicated prevention is targeted at subgroups at high risk and individuals with detectable but subclinical symptoms of a mental disorder. Where interventions were delivered to a whole class or school with the same at-risk characteristic (such as schools in low-income areas) they were combined with universal interventions, and differences examined via subgroup analysis (see inequality outcome). All types of control group were eligible.

We excluded studies in which more than 40% of participants had an identifiable mental disorder. Studies addressing emotional wellbeing, positive mental health, mental health promotion, awareness, or literacy were not eligible for inclusion, unless the explicit aim of the trial was the prevention of anxiety and depression. Interventions designed to target problems potentially on the causal pathway to a mental health disorder (eg, stress, bullying, substance abuse) were also excluded. We excluded interventions aiming to help children and young people manage the consequences of a specific event or situation (eg, divorce, exams). Digital interventions were excluded unless they were delivered in the education setting or were an adjunct to a wider programme delivered in the educational setting (eg, as homework). Studies using schools as the source of recruitment but where the intervention was not school based were excluded. Primary care, outpatient, and inpatient settings were excluded.

Study inclusion and exclusion was independently assessed by two reviewers and disagreement resolved by a third, if necessary (SD, JP, DC, PC, SH). Data were extracted by one reviewer and double checked by a second (SD, JP, DC, PC, and CF). Study authors were contacted for additional data.

The results reported in this paper are part of a larger project, the protocol for which is registered with PROSPERO, number CRD42016048184. Results are reported in accordance with the PRISMA extension statement for network meta-analysis.^27^

### Outcomes

The primary outcome was effectiveness based on self-report anxiety or depression (defined according to standard diagnostic criteria such as DSM-5, or as measured by a validated scale); self-reported wellbeing (defined by study author); and self-reported suicidal ideation, behaviour, or self-harm. Reducing inequalities in health is a key aim of public health interventions. We planned to investigate the effect of the interventions on inequalities in health via subgroup analyses examining intervention effects by socioeconomic status, sex, and ethnicity, as defined by study authors. These were post-hoc subgroup analyses and have been included in our change from protocol statement (appendix p 100). If studies reported multiple symptom scales, we applied a prespecified hierarchy to select the most appropriate outcome (appendix p 14). If a study only reported a composite outcome (eg, total Depression, Anxiety and Stress scale or Strengths and Difficulties Questionnaire [SDQ]), it was included in the review but not the meta-analysis.

The primary endpoint was assessed immediately post-intervention. We also report results for mid-term (p 15)(6–12 months) and longer-term (13–24 months) follow-up categories. If studies reported multiple timepoints within a category (eg, at both 6 and 12 months), we prioritised the longer follow-up. If studies had follow-up at 25 months or later, we report this in the appendix (p 16). Intervention and control descriptions are provided in the appendix (p 15). Following classifications adopted in previous reviews,^16^, ^29^, ^30^, ^31^, ^32^, ^33^ the content of psychological and psychosocial interventions was classified into five broad intervention types: cognitive behavioural therapy (CBT), behavioural, third-wave, interpersonal, and psycho-supportive interventions; physical interventions were classified as exercise, relaxation, or biofeedback interventions. Psychoeducational, mindfulness and relaxation, and bias modification intervention categories were also identified. Network meta-analysis also provides an opportunity to distinguish between different types of control conditions. This is based on evidence that use of different control conditions can result in different effect size estimates.^34^, ^35^ We identified four types: no intervention, waitlist, usual curriculum, and attention controls.

Additional outcomes will be reported in the full monograph on which this paper is based (NIHR 15/49/08).^28^ These are listed in the appendix for transparency (p 13).

### Statistical analysis

Analyses are based on study completers (available case analysis). We extracted data for number randomised to each intervention group at baseline, baseline mean and SD, number assessed at follow-up, and follow-up mean and SD (for each timepoint listed above). When cluster randomised trials did not explicitly account for clustering, we followed guidance from the Cochrane Handbook to estimate the approximate sample size. When intracluster correlation coefficients (ICC) were not available, we used estimates from studies within this review (appendix p 72) as recommended by the Cochrane Handbook.^36^

We assessed risk of bias using the Cochrane Risk of Bias tool.^36^

### Statistical methods

We did network meta-analyses within a Bayesian framework, implemented using OpenBUGS^37^ (appendix p 19). Both fixed and random effects models were fitted. Heterogeneity was assessed by examining the posterior median between-study SD (τ) and 95% credible intervals (CrIs) from the random effects model and by comparing model fit of the fixed and random effects models. Further information on checking assumptions, prior distributions, convergence, and the statistical models fitted are reported in the appendix (p 21).

For continuous outcomes we report standardised mean differences (Hedge's *g*) to summarise intervention effects, with 95% CrIs. For dichotomous outcomes we report odds ratios and 95% CrIs. We did separate network meta-analyses by population and educational setting (primary, age 4–11 years; secondary, age 12–18 years; and tertiary, age older than 18 years). Studies in mixed-age settings were excluded from the network meta-analysis (k=5). Meta-regression was done to examine whether intervention effects differed by method of intervention delivery and who delivered the intervention (appendix p 82). We did subgroup analyses to assess whether intervention effects differed by intended focus of the intervention—eg, whether interventions addressing anxiety had a larger effect on anxiety outcomes than interventions intended to focus on depression, but which also recorded anxiety outcomes.

For primary outcomes we did sensitivity analyses excluding studies at high or unclear risk of bias on the domains of random sequence generation and allocation concealment. Further sensitivity analyses were done to examine the robustness of the findings to the assumed ICC value for cluster randomised trials. Small study effects were investigated using comparison-adjusted funnel plots.^38^

### Role of the funding source

The funder of the study had no role in study design, data collection, data analysis, data interpretation, writing the report, or the decision to submit for publication. DMC, JLL, SRD, NJW, and JP had access to the data in the study, and DMC had final responsibility for the decision to submit for publication.

## Results

**Figure 1:**
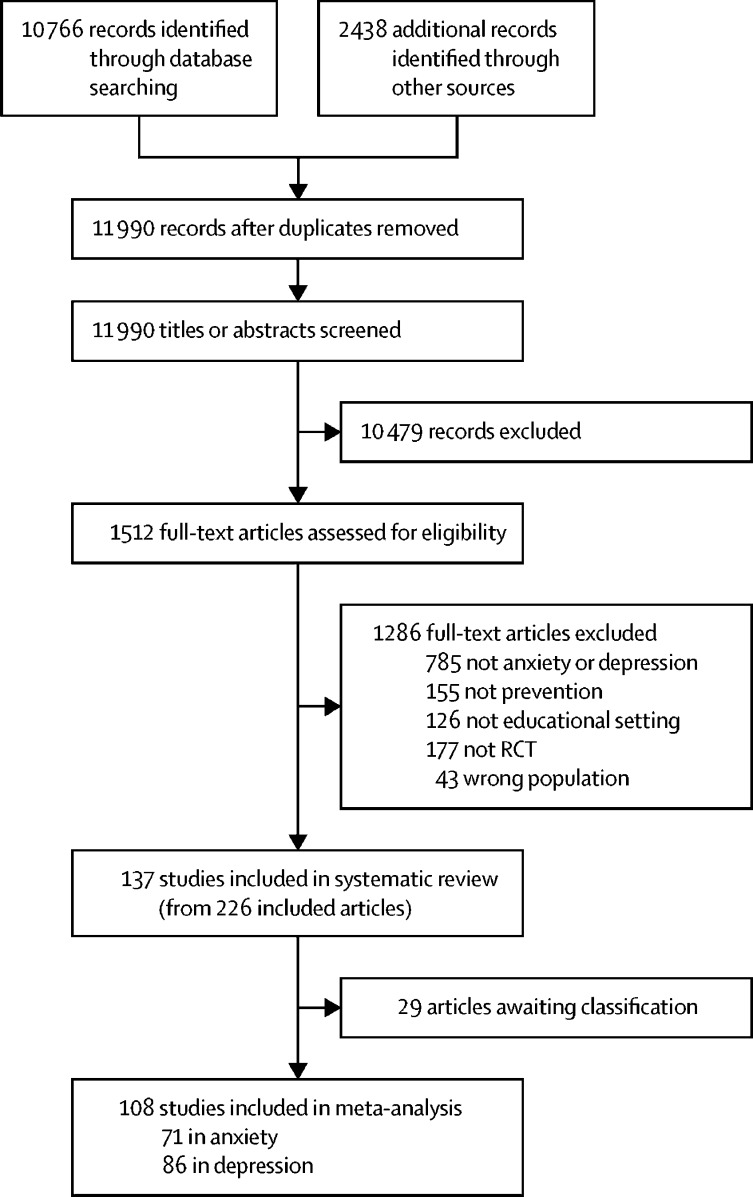
Study selection process Articles awaiting classification reasons are listed in the appendix (p 98).RCT=randomised controlled trial.

We screened 11 990 citations, from which we retrieved 1512 full-text articles (figure 1). 137 studies including 56 620 participants were included in the review.

Study characteristics are reported in the appendix (pp 24–30). Studies were published between 1982 and 2018 and included between 13 and 5633 participants (median 174). 123 of 137 studies were published in peer-reviewed academic journals, and 14 studies were doctoral dissertations. 69 were individually randomised trials and 68 studies were cluster randomised. Approximate sample sizes were calculated for 59 cluster trials. 126 studies were done in high-income countries, and 11 were done in low-income or middle-income countries. Of the studies done in high-income countries, eight were done in lower-income settings as defined by authors.

76 studies were classified as universal and 61 as targeted (51 indicated, ten selective). 39 studies were implemented in primary school, 85 in secondary school, eight in tertiary education, and five in mixed-age settings. The primary focus of 41 studies was the prevention of anxiety, 62 focused on the prevention of depression, and 34 addressed both anxiety and depression.

103 studies included an intervention based on CBT, 11 studies included a relaxation or mindfulness-based intervention, four included interventions based on interpersonal therapy, six included a third-wave intervention, four included a behavioural intervention, four used methods of biofeedback, four included an exercise intervention, and two used bias modification approaches. One study used an occupational therapy-based intervention. With regards to non-active comparators, 41 studies were waitlist controlled, 36 were usual curriculum controlled, 28 had a no-intervention control, and 19 used an attention control.

For the main outcome of self-reported depression or anxiety, 123 studies reported a post-intervention endpoint, 73 reported a follow-up between 6–12 months, and 18 reported follow-up between 13–24 months. Three studies were not included in the meta-analysis as they only reported a composite outcome. All reported SDQ. The number of sessions ranged from two to 48 (mean 10·5, SD 6·1). As a proxy for intervention dose, we calculated the intervention intensity as total session time (number of sessions × length in min); this ranged from 60 min to 3240 min (mean 669·7 min, SD 471·9). 96% of interventions were delivered to whole classrooms or small groups. Most were delivered by personnel external to the educational setting; 49% used an intervention delivered by an external mental health professional and 5% by miscellaneous external professionals. 20% of studies used interventions delivered by teachers and 4% by school counsellors. 15% of studies involved a combination of both teaching and psychology professionals. Four studies implemented interventions exclusively via computer.

**Figure 2:**
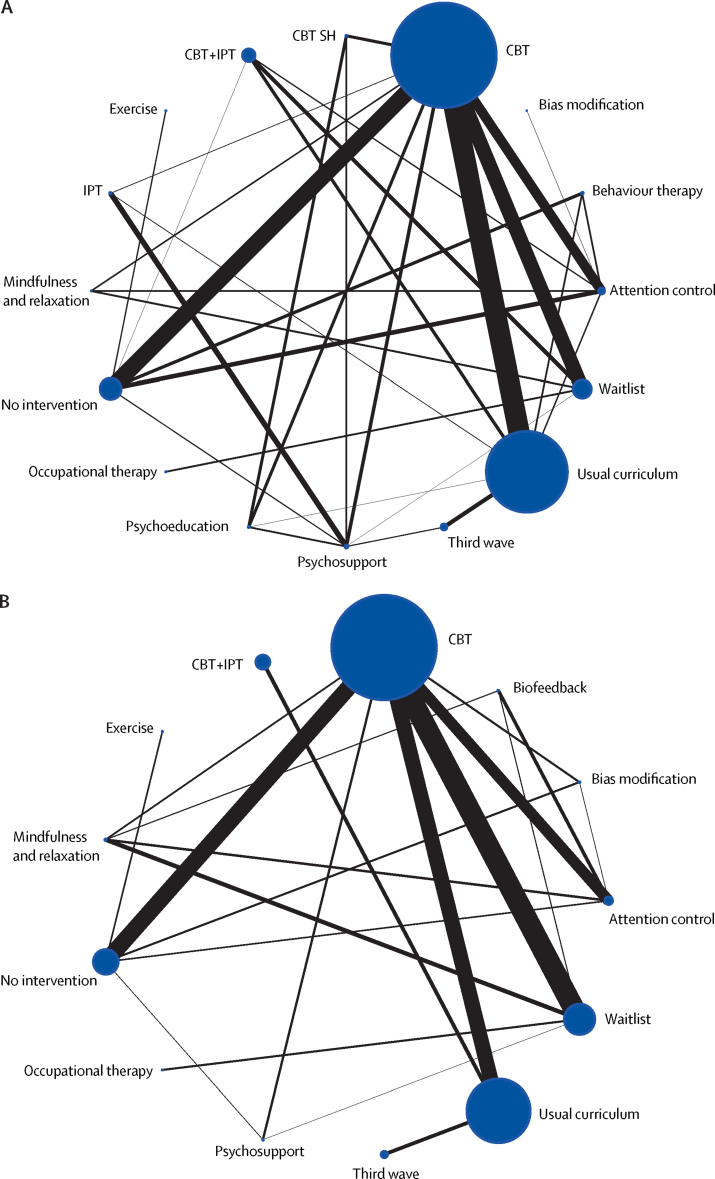
Network meta-analysis of eligible comparisons for depression and anxiety Depression (A) and anxiety (B). Width of solid black lines is proportional to the number of trials comparing each pair of interventions. Size of node (circle) is proportional to the number of randomly assigned participants receiving that intervention. CBT=cognitive behavioural therapy. IPT=interpersonal therapy. CBT SH=cognitive behavioural therapy self-help. Third wave=third wave CBT-based therapies.

Figure 2 shows the combined network of intervention comparisons, for anxiety and depression outcomes. Separate setting and population specific networks are reported in the appendix (p 40).

20 of the 137 studies were assessed at being at low risk of bias for both random sequence generation and allocation concealment (appendix p 43). 41 studies reported a suitable randomisation approach but did not report details of allocation concealment. 82 studies were judged as having unclear risk of bias for random sequence generation and allocation concealment.

Between-study posterior median SDs (τ) were indicative of moderate heterogeneity. Model fit and comparison statistics suggested evidence of heterogeneity (appendix p 21). All reported results are from a random effects network meta-analysis model. When direct comparisons are available, results for standard pairwise meta-analyses are reported in the appendix (p 44). The table shows posterior mean ranks and 95% CrIs for the post-intervention timepoint. Substantial uncertainty surrounds the mean rank across all populations and settings. As such we are unable to recommend any one type of intervention as the most effective for preventing anxiety or depression.

**Table:** Posterior mean rank and 95% CrIs by population and setting, for primary endpoint measured immediately post-intervention

	**Universal primary population**		**Universal secondary population**		**Targeted primary population**		**Targeted secondary population**	
	Depression (n=6)*	Anxiety (n=5)*	Depression (n=10)*	Anxiety (n=7)*	Depression (n=4)*	Anxiety (n=5)*	Depression (n=11)*	Anxiety (n=9)*
Usual curriculum	3·9 (1–6)	3·2 (1–5)	6·3 (3–9)	5·5 (3–7)	..	..	8·0 (3–11)	..
Waitlist	3·2 (1–6)	3·5 (1–5)	6·3 (2–10)	4·9 (2–7)	3·3 (1–4)	4·0 (2–5)	9·7 (6–11)	7·9 (7–8)
No intervention	4·8 (2–6)	4·7 (2–5)	7·1 (3–10)	4·4 (2–7)	..	..	7·9 (5–11)	5·2 (2–7)
Attention control	3·3 (1–6)	1·6 (1–4)	8·0 (3–10)	3·4 (2–7)	1·7 (1–4)	2·4 (1–5)	3·5 (2–9)	4·1 (2–7)
CBT	2·6 (1–5)	2·0 (1–3)	4·8 (2–8)	2·9 (2–5)	2·2 (1–4)	2·1 (1–4)	5·8 (4–8)	5·8 (3–7)
Behavioural therapy	3·2 (1–6)	..	5·8 (1–10)	..	..	..	..	..
Third wave	..	..	5·2 (1–9)	5·9 (2–7)	..	..	1·0 (1–1)	..
CBT+IPT	..	..	2·4 (1–8)	..	..	..	..	..
IPT	..	..	5·5 (1–10)	..	..	..	3·8 (2–8)	..
Mindfulness	..	..	..	1·0 (1–1)	..	..	..	5·4 (2–8)
Psychoeducation	..	..	3·7 (1–10)	..	..	..	8·8 (5–11)	..
Psychosupport	..	..	..	..	..	..	8·1 (4–11)	9·0 (9–9)
Occupational therapy	..	..	..	..	2·9 (1–4)	4·0 (1–5)	..	..
Biofeedback	..	..	..	..	..	2·4 (1–5)	..	3·1 (1–7)
Bias modification	..	..	..	..	..	..	3·5 (2–10)	3·1 (1–6)
Exercise	..	..	..	..	..	..	6·0 (2–11)	1·4 (1–4)

All data are posterior mean rank (95% CrIs). Lower rank numbers are better, with 1 the best and 10 the worst. CrI=credible interval from random effects network meta-analysis. CBT=cognitive behavioural therapy. IPT=interpersonal therapy.

* Number of treatments in each network.

**Figure 3:**
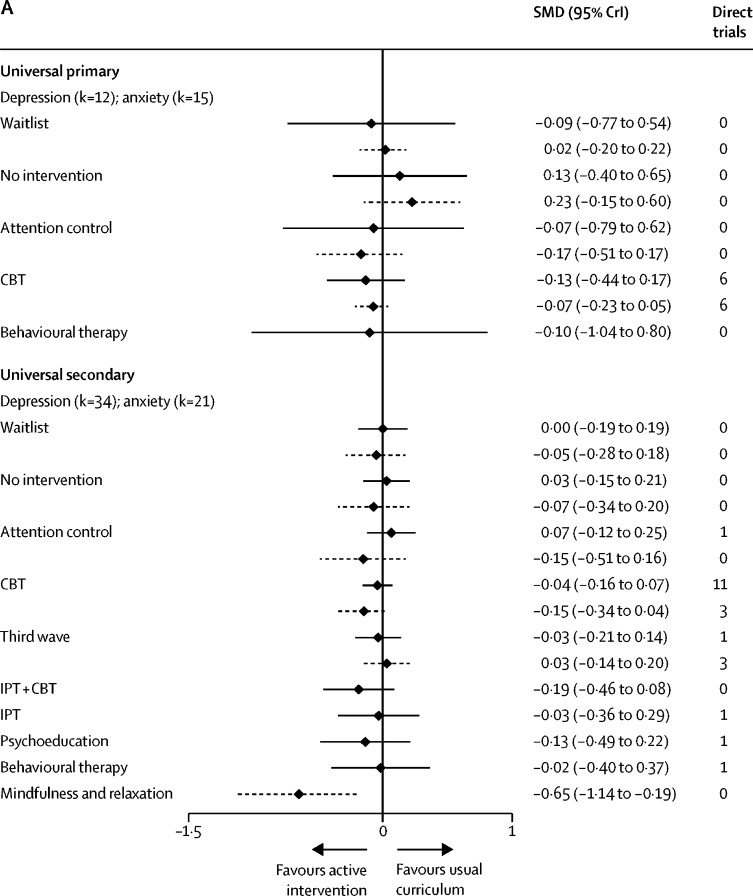

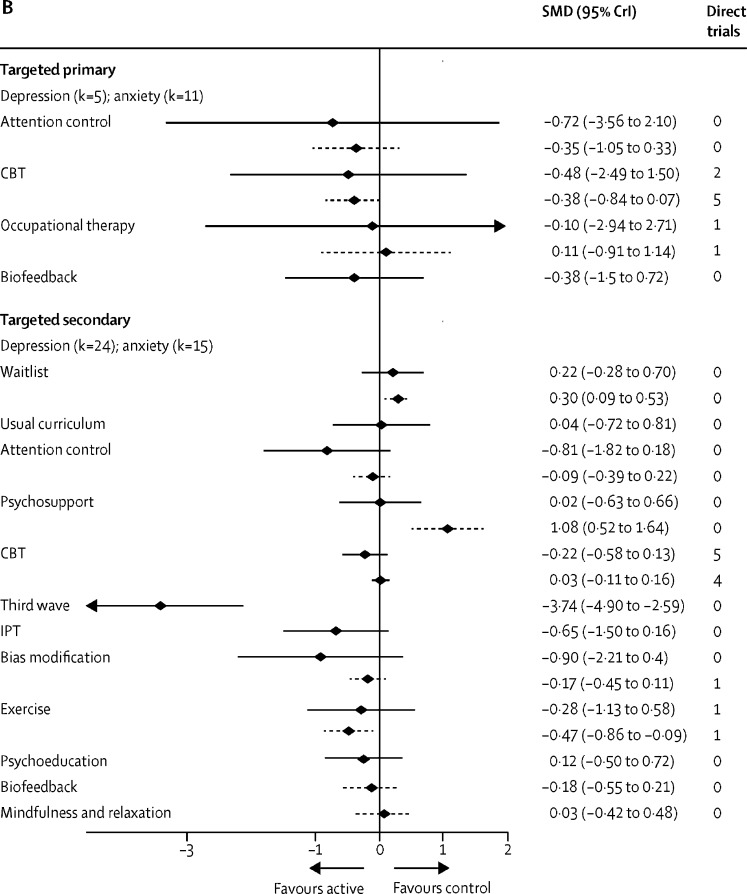
Self-report depression and anxiety immediately post-intervention (A) Universal population. Active intervention is displayed relative to the reference intervention Usual Curriculum. (B) Targeted population. For targeted primary the reference intervention displayed is waiting list. For targeted secondary the reference is no intervention. Effect estimates are based on combination of direct and indirect evidence from a random effects network meta-analysis. Direct trials are the number of head-to-head trials in the network making that comparison. Solid black lines denote the depression outcome and the dashed lines denote the anxiety outcome. k=number of studies included in network meta-analysis. SMD=standardised mean difference. CrI=credible interval. CBT=cognitive behavioural therapy. IPT=interpersonal therapy.

In universal primary settings, there was no evidence that any intervention reduced depression or anxiety symptoms relative to usual curriculum, although there is weak evidence to suggest that CBT might be beneficial for anxiety symptoms (SMD −0·07, 95% CrI −0·23 to 0·05; τ=0·10, figure 3A). In universal secondary settings, mindfulness and relaxation interventions showed a reduction in anxiety symptoms relative to usual curriculum (−0·65, −1·14 to −0·19; τ=0·11). There is weak evidence to suggest that CBT reduced anxiety symptoms (−0·15, −0·34 to 0·04). There is no evidence that any one intervention reduced depression symptoms in universal secondary settings.

In targeted primary settings, there is no evidence that any type of intervention is effective relative to waitlist for reduction of depression or anxiety symptoms (figure 3B). In targeted secondary settings, exercise appears effective for reducing anxiety symptoms relative to a no intervention comparator (SMD −0·47, 95% CrI −0·86 to −0·09; τ=0·06). For the prevention of depression symptoms, third-wave interventions appear effective (−3·74, −4·90 to −2·59; τ=0·37). However, each estimate is based on a single trial, which contributed to the network via a spur (appendix p 40).

Only eight tertiary institution-based interventions met our inclusion criteria, of which seven were for a targeted population. Network meta-analysis results for targeted university-based interventions are reported in the appendix (p 51).

Results by population and setting for the 6–12 months and 13–24 months follow-up timepoints are reported in the appendix (p 47). In universal primary school settings, there was little evidence that any type of intervention prevents depression or anxiety symptoms relative to usual curriculum. At 6–12 months in universal secondary school settings, weak evidence suggested that third-wave CBT-based therapies (SMD −0·13, 95% CrI −0·27 to 0·01) and CBT plus interpersonal psychotherapy (−0·10, −0·26 to 0·05; τ=0·08) might be effective relative to usual curriculum for depression but not anxiety symptoms. At 13–24 months, there was no evidence that any type of intervention is effective for the prevention of anxiety or depression in either primary or secondary universal settings.

For targeted interventions at 6–12 months post-intervention, there is no evidence that any type of intervention was effective for reduction of depression or anxiety symptoms in either primary or secondary settings. At 13–24 months, CBT reduced depressive symptoms relative to waitlist in targeted primary settings (SMD −0·50, 95% CrI −0·96 to −0·05). In targeted secondary settings at 13–24 months, CBT reduced anxiety symptoms (−0·26, −0·52 to −0·01) but not depressive symptoms. These findings are based on a single study.14 studies reported a measure of psychological wellbeing, quality of life, or life satisfaction. Network meta-analysis was not possible because of insufficient data, and data are reported by outcome measure and study in the appendix (pp 53–58). 34 studies provided information on suicidal ideation, suicide attempt, or self-harm behaviours. There was no evidence to suggest that educational setting-based interventions to prevent common mental disorders affect suicidal ideation or self-harm (appendix pp 53–59).

To explore inequalities in health, we did a post-hoc subgroup analysis to examine whether intervention effects varied according to socioeconomic status (appendix pp 59–70). There was no evidence of a difference by socioeconomic status for depression or anxiety in primary settings. However, in secondary school settings, results suggest that interventions delivered in lower socioeconomic status settings were less effective than those in higher or mixed socioeconomic status settings. Due to insufficient data, we did not do subgroup analyses for sex and ethnicity.

We did sensitivity analyses for the main outcomes of self-reported depression and anxiety post-intervention. We removed studies at high or unclear risk of bias for randomisation and allocation concealment, leaving only five studies in the universal secondary depression network and three studies in the secondary anxiety network. For targeted secondary interventions, only four studies were included in the low risk of bias analyses for self-report depression, and three studies for self-report anxiety. Findings were unchanged (appendix p 43).

Results were robust to preplanned sensitivity analyses examining assumed ICC values. Comparison adjusted funnel plots suggested that smaller studies report more beneficial results than larger studies among non-active controlled trials for universal anxiety outcomes (appendix p 77). There was no evidence of effect modification by facilitator or mode of delivery; however, point estimates indicate a slight preference for interventions facilitated by mental health professionals. Intended focus of the intervention could be important; interventions focused on preventing anxiety seemed to have a larger effect on anxiety symptoms, and interventions focused on preventing depression appeared to have a more beneficial effect on depressive symptoms. However, because of the absence of participant blinding, a Hawthorne effect cannot be ruled out and the likelihood of publication bias casts further doubt on this finding.

## Discussion

To the best of our knowledge, this is the first network meta-analysis of interventions to prevent anxiety and depression in children and young people. We find insufficient evidence to conclude that educational setting-based interventions are effective for the prevention of anxiety and depression in children and young people. Only 15% of included studies were rated as being at low risk of bias, and there was substantial uncertainty in the intervention rankings, and evidence of publication bias. The beneficial effect observed in previous meta-analyses is possibly an artefact of conflating control conditions and combining primary and secondary school settings (appendix pp 88–97). We suggest that future studies should be commissioned only if they are active-controlled, such as an attention control or an alternative intervention.

Our findings should be interpreted in the context of several limitations. We only searched four electronic databases, although our searches retrieved all but two eligible studies included in previously published systematic reviews. We were unable to assess 29 papers for eligibility in time for analysis (figure 1; appendix p 98). Although our review includes 137 studies, only 108 contributed data to the network meta-analysis. Our primary outcomes were self-report symptoms and therefore could be at higher risk of performance bias than observer-rated outcomes. Furthermore, the outcome of interest for decision makers might be clinical diagnosis and not symptoms. However, clinical diagnosis was rarely reported because of short follow-up periods in most of the studies. Only 18 studies reported follow-up longer than 1 year, and only seven reported follow-up longer than 2 years. Our findings regarding sustainability of intervention effects are inconclusive.

The upper age limit of 18 years at baseline, coupled with the exclusion of remotely delivered digital and online interventions and clinic-based or health service-based interventions, restricted the number of university-based interventions included. Another review,^39^ with broader inclusion criteria, included 62 studies for preventing anxiety and depression in university students, compared with eight in our paper.^39^ As such, our findings for tertiary settings should not be generalised, and we have not included them in the main text.

Our typology of interventions and control conditions was based on previous literature,^29^, ^30^, ^31^, ^32^, ^35^ piloting, and discussion in our team. Nevertheless, we acknowledge that the process of categorising complex interventions is subjective^40^ and future work should agree on a process for node-making as well as agreeing on a taxonomy for classifying psychological interventions. Reporting of experimental interventions was adequate for classification. By comparison, control conditions were less well reported, particularly the usual curriculum comparator. Few studies reported enough detail to judge how randomisation or allocation concealment had been done. Improved reporting of complex interventions should be addressed in future publications, adhering to the TIDieR framework.^41^

In this paper we focused on interventions for which the primary aim was the prevention of common mood disorders. As such we excluded interventions focused on mental health promotion (MHP). Here we followed the Institute of Medicine's definition of MHP as interventions, which aim to “enhance an individuals' ability to achieve developmentally appropriate tasks (developmental competence) and a positive sense of self-esteem, mastery, well-being, and social inclusion and to strengthen their ability to cope with adversity”.^26^ Evidence has suggested that wellbeing is only weakly associated with mental illness in children (*r*=0·2),^42^ raising doubts that interventions targeting one will necessarily affect the other.^43^ However, some interventions aim to address both prevention and promotion, such as the Aussie Optimism Programme^44^ (which was included here). In such instances, we referred to trial registrations and protocols to inform our inclusion decision. However, in their absence it was difficult to operationalise this distinction, which might be considered a limitation of our work. In common with previously published systematic reviews^15^, ^16^, ^17^, ^18^, ^19^ we did not include interventions that primarily addressed substance use, bullying, or stress prevention, although these factors have been shown to be associated with later mental health problems. These decisions were pragmatic, taken to minimise potential between-study heterogeneity and to increase comparability of our findings with previous work. As there have been calls to reframe mental health towards a broader dimensional approach,^45^ future work should consider network meta-analysis of MHP and emotional wellbeing interventions.

Whole-school interventions have also been suggested as a structural prevention approach and as an alternative to a narrow focus on the individual or group, such as those considered in this review. However, although whole-school interventions have shown promise for physical health outcomes^46^ and emotional wellbeing^47^ there is little evidence that they are effective for prevention of common mental disorders.^46^, ^48^ Despite this, we surmise that future preventive interventions should not focus solely on the individual child's cognitions, emotions, or mood without also addressing the wider familial and structural context in which interventions are implemented.^49^ Insights from systems science, which conceptualise schools as complex adaptive systems, might also be useful when considering future interventions.^50^

## Data sharing

The dataset on which this paper is based will be made available on publication of the NIHR report (due 2020).
