# Correction to *Lancet Psychiatry* 2019; 6: 1011–20

**DOI:** 10.1016/S2215-0366(20)30352-7

**Published:** 2020-09

*Caldwell D M, et al. School-based interventions to prevent anxiety and depression in children and young people: a systematic review and network meta-analysis.* Lancet Psychiatry 2019; **6:** 1011–20—In this Article, the standardised mean difference and 95% credible interval of third-wave interventions should have read −0·68 (−1·83 to 0·47) in figure 3B. In Results, reference to these data on the prevention of depressive symptoms has been removed. In figure 1, the final box should have read 109 studies included in meta-analysis. These corrections have been made to the online version as of Aug 20, 2020.

