# Supplementary material for: School-based interventions to prevent anxiety and depression in children and young people: a systematic review and network meta-analysis
Source: Lancet Psychiatry. 2019 Dec;6(12):1011–20. doi: 10.1016/S2215-0366(19)30403-1 (PMC7029281; doi:10.1016/S2215-0366(19)30403-1)
Supplement: Supplementary appendix [file mmc1.pdf]

THE LANCET
Psychiatry

Supplementary appendix

This appendix formed part of the original submission and has been peer reviewed. We post it as supplied by the authors.

Supplement to: Caldwell MC, Davies SR, Hetrick SE, et al. School-based interventions to prevent anxiety and depression in children and young people: a systematic review and network meta-analysis. *Lancet Psychiatry* 2019; published online Nov 13. https://doi.org/10.1016/S2215-0366(19)30403-1.

Contents

Search strategies used for each database: ... 2
Additional outcomes to be included in NIHR monograph ... 13
Decision Rule for choosing between multiple-report scales ... 14
Intervention and control classifications applied in this study ... 15
Study follow-up and timepoints (months from end of intervention) ... 16
Further statistical details and OpenBUGS code used for network meta-analysis ... 19
Model fit, heterogeneity and convergence details: self-report depression ... 21
Study characteristics: Universal interventions ... 24
Study characteristics: Targeted interventions (selective and indicated) ... 27
Attendance and Fidelity ... 29
Facilitator fidelity/ integrity ... 35
Setting and population specific network plots: immediately post-intervention ... 38
Risk of bias assessment for included studies ... 41
Standard pairwise meta-analyses: results ... 42
Results at follow-up timepoints: 6-12 months and 13-24 months. ... 45
University based interventions: forest plot ... 49
University based interventions: Mean Rank and 95% credible intervals ... 50
Results for wellbeing outcome immediately post-intervention ... 51
Suicidal ideation, behaviour and self-harm outcomes ... 53
Descriptions of socio-economic status, gender and ethnicity: universal interventions ... 56
Descriptions of socio-economic status, gender and ethnicity: targeted interventions ... 62
Results from subgroup analysis for socio-economic status ... 67
Sensitivity analysis to ICC values ... 69
Comparison-adjusted funnel plots to explore small study effects ... 73
Meta-regression and subgroup analyses ... 78
Comparison of results from recent meta-analyses and the results from the NMA ... 84
Post-hoc exploration of control groups and influence on findings ... 85
Relative effects of active interventions compared to separate controls: NMA ... 89
References to studies awaiting screening ... 92
Changes from protocol ... 94
References to included studies ... 95
Complete results of network meta-analysis by population, setting and outcome: post-intervention ... 100

Search strategies used for each database:

Medline

1. CHILD, PRESCHOOL/ or CHILD/ or ADOLESCENT/ or
YOUNG ADULT/
2. (child* or boy* or girl* or kids or juvenil* or minors or paediatric* or pediatric* or adolesc* or preadolesc* or preadolesc* or pubert* or pubescen* or prepube* or prepube* or teen* or (young adj (adult* or people or patient* or men* or women* or male or female or survivor* or offender* or minorit*)) or youth* or student* or undergrad*).ti,ab,kf.
3. (child* or adolesc* or paediatr* or pediatr*).jn.
4. or/13
5. EDUCATION/
6. SCHOOLS/ or SCHOOLS, NURSERY/
7. SCHOOL HEALTH SERVICES/ or SCHOOL NURSING/
8. STUDENTS/ or UNIVERSITIES/
9. (preschool or kindergarten or school* or college* or campus* or classroom* or curricul* or teacher or gatekeeper or pupil*).ti,ab,kf.
10. PEER GROUP/
11. ((peer or peers) adj (education or group or relation* or
support* or intervention* or leader*)).ti,ab,kf.
12. student* union.ti,ab,kf.
13. ((church or communit* or holiday* or religi* or spiritual* or youth or vacation) adj2 (camp or club or group)).ti,ab,kf.
14. ((church or communit* or holiday* or religi* or spiritual* or youth or vacation) adj based).ti,ab,kf.
15. or/5-14
16. ADAPTATION, PSYCHOLOGICAL/
17. EMOTIONS/
18. MENTAL HEALTH/
19. SOCIAL ADJUSTMENT/
20. exp STRESS, PSYCHOLOGICAL/
21. (mental health or mental* ill* or psychiatric).ti,ab,kf.
22. (wellbeing or well being).ti,ab,kf.
23. (stress* or distress*).ti,ab,kf.
24. or/16-23
25. DEPRESSION/
26. DEPRESSIVE DISORDER
27. MOOD DISORDERS
28. (depress* or dysthymi* or affective disorder* or affective
symptom* or mood* or mental).ti.
29 (depress* adj2 (adolescent* or child* or anaclitic* or episode* or disorder or scale* or score* or symptom* or unipolar)).ti,ab,kf.
30. ((depress*or mood* or mental or psychological or wellbeing or well being or emotion*) adj2 (improve* or onset or prevent* or reduc*)).ti,ab,kf.
31. ((Axis 1 or Axis I) adj disorder*).ab.
32. or/25-31
33. exp ANXIETY DISORDERS/
34. ANXIETY/
35. anxi*.ti.

36. (anxi* adj3 (adolescent* or child* or disorder* or general* or interpersonal or separation or social*)).ti,ab,kf.
37. (phobi* or agoraphobi* or PTSD or post trauma* or posttrauma or panic* or OCD or obsess* or compulsi* or GAD or stress disorder* or stress reaction* or acute stress or neurosis or neuroses or neurotic or psychoneuro* or
(school adj2 (refusal or avoid*)) or social avoidance or mutism).ti,ab,kf.
38. (((anxi* or fear or fright) adj3 (perform* or athlet* or music* or act* or test* or exam*)) or math* anxiety).ti,ab,kf.
39. (public adj3 (speak* or speech)).ti,ab,kf.
40. or/33-39
41. CONDUCT DISORDER/
42. CHILD BEHAVIOR DISORDERS/
43. JUVENILE DELINQUENCY/
44. SOCIAL BEHAVIOR/
45. SOCIAL BEHAVIOR DISORDERS/
46. ((behavi* or conduct or personalit*) adj2 (agressi* or nonagressi* or antisocial or anti social or dyssocial or defiant or delinquen* or disturb* or disrupt* or disorder* or internali#ing or externali#ing or problem*)).ti,ab,kf.
47. ((conduct or behavi* or antisocial or anti social or dyssocial or emotional* or internali#ing or externali#ing) adj3 (problem* of difficult* or psychopathol*)).ti,ab,kf.
48. (oppositional adj3 (defiant* or disorder*)).ti,ab,kf.
49 or/41-48
50 PREVENTIVE HEALTH SERVICES/ or "EARLY INTERVENTION (education)"/ or HEALTH LITERACY/ or PATIENT EDUCATION AS TOPIC/ or HEALTH PROMOTION/ or PRIMARY PREVENTION/ or SECONDARY PREVENTION/
51. prevention & control.fs.
52. prevent*.ti,kf.
53. prevention of.ab,kf.
54. (prevent* adj2 (intervention or educat* or pilot or program* or project or protocol* or training or universal or targeted or primary or secondary or selective or indicated or study or trial)).ti,ab,kf.
55. ((early or brief) adj intervention*).ti,ab,kf. 56 ((universal or targeted) adj2 (program* or intervention*)).ti,ab,kf.
57. (vulnerabl* or at risk or (risk adj2 reduc*)).ti,ab,kf.
58. RISK/ or RISK FACTORS/
59. exp ACCIDENTS/
60. BEREAVEMENT/ or GRIEF/
61. SOCIAL PROBLEMS/
62. BULLYING/
63. CHILD OF IMPAIRED PARENTS/
64. CHILD, ORPHANED/
65. CRIME VICTIMS/
66. exp DISASTERS/
67. DIVORCE/
68. LIFE CHANGE EVENTS/
69. RUNAWAY BEHAVIOR/
70. URBAN POPULATION/
71. RURAL POPULATION/
72. SURVIVORS/
73. VIOLENCE/
74. WARFARE/

75. social problems/ or exp civil disorders/ or exp crime/ or exp human rights abuses/ or exp parental death/ or poverty/ or exp social behavior disorders/ or domestic violence/ or exp child abuse/ or exp ethnic violence/ or physical abuse/ or exp terrorism/ or torture/ or exposure to violence/ or exp
"warfare and armed conflicts"/
76."dissent and disputes"/ or family conflict/ or psychosocial deprivation/
77. or/50-76
78. RANDOMIZED CONTROLLED TRIAL/ or PRAGMATIC CLINICAL TRIAL/
79. Randomized Controlled Trial.pt.
80. (randomi#ed or randomi#ation).ab,ti,kf.
81. (RCT or (random* adj3 (administ* or allocat* or assign* or class* or cluster* or control* or determine* or divide* or distribut* or expose* or fashion or number* or place* or recruit* or subsitut* or treat*))).ab.
82. at random.ab.
83. placebo.ab.
84. trial.ti,kf.
85. or/78-84
86. (treatmentasusual or (treatment* adj2 usual) or (standard adj2 care) or (standard adj2 treatment) or (routine adj2 care) or (usual adj2 medication*) or (usual adj2 care) or TAU).ti,ab,kf.
87. (waitlist* or waitlist* or waitinglist* or wait* list* or (waiting adj (condition or control)) or WLC).ti,ab,kf.
88. (((delay* adj3 (start or treatment*)) or no intervention or no treatment* or notreatment or non treatment* or nontreatment* or nontreatment
or minim* treatment* or untreated group* or untreated control* or without any treatment) and (control* or group*)).ti,ab,kf.
89. ((no intervention* or non intervention* or nonintervention*
or without any intervention*) and (control* or group*)).ti,ab,kf.
90. or/86-89
91. 85 or 90
92. 4 and 15 and (24 or 32 or 40 or 49) and 77 and 91
93. ((universal or indicated or targeted or at risk) and prevent* and (anxiety or depress* or conduct) and (child* or adolesc* or school*)).mp.
94. ((prevent* adj (program* or intervention)) and (anxiety or depress* or conduct) and (child* or adolesc* or school*)).mp.
95. 93 or 94

PsycINFO
1. "3580".cc. [=Classification Code: Educational/Vocational Counseling & Student Services]
2. exp school based intervention/
3. school*.ti.
4. or/1-3
5. (child* or boy* or girl* or kids or juvenil* or minors or paediatric* or pediatric* or adolesc* or preadolesc* or pre-adolesc* or pubert* or pubescen* or prepube* or pre-pube* or teen* or (young adj (adult* or people or patient* or men* or women* or male or female or survivor* or offender* or minorit*)) or youth* or student* or undergrad*).ti,ab,id.
6. pediatrics/
7. child psychiatry/ or child psychopathology/ or child psychology/
8. adolescent psychiatry/ or adolescent psychopathology/ or adolescent psychology/
9. child psychotherapy/ or adolescent psychotherapy/
10. childhood development/ or early childhood development/ or adolescent development/

11. students.hw.
12. ("160" or "180" or "200" or "320").ag.
[=Age Group Field/Codes: preschool 2-5; school age 6-12; adolescence 13-17; young adulthood 18-29]
13. or/5-12
14. education/
15. education/ or elementary education/ or high school education/ or higher education/ or middle school education/ or multicultural education/ or nontraditional education/ or preschool education/ or private school education/ or public school education/ or secondary education/ or special education/
16. schools/ or academic settings/ or boarding schools/ or charter schools/ or exp colleges/ or elementary schools/ or graduate schools/ or high schools/ or institutional schools/ or junior high schools/ or kindergartens/ or middle schools/ or nongraded schools/ or nursery schools/
17. school environment/ or college environment/
18. school facilities/ or campuses/ or classrooms/ or "learning centers (educational)"/ or school libraries/
19. community facilities/ or community mental health centers/ or exp libraries/
20. "summer camps (recreation)"/
21. curriculum/
22. exp extracurricular activities/ or exp after school programs/
23. (preschool or nursery or kindergarten or school* or college* or university or universities or campus* or classroom* or curricul* or gatekeeper or pupil*).ti,ab,id.
24. peers/ or peer counseling/ or peer tutoring/
25. ((peer or peers) adj (education or group or relation* or support* or intervention* or leader*)).ti,ab,id.
26. student* union.ti,ab,id.
27. ((church or communit* or holiday* or religi* or spiritual* or youth or vacation) adj3 (camp* or club*1 or group*1)).ti,ab,id.
28. ((primary or secondary or tertiary) adj educat*).ti,ab,id.
29. ((detention or refugee*) adj (camp*1 or centre*1 or center*1)).ti,ab,id.
30. or/14-29
31. "3300".cc. [=Classification Code: Health & Mental Health Treatment & Prevention]
32. primary mental health prevention/
33. mental health/ or well being/
34. Stress/ or Distress/
35. emotional adjustment/
36. "resilience (psychological)"/ or coping behavior/ or psychological stress/
37. *affective disorders/
38. major depression/ or dysthymic disorder/ or reactive depression/ or "depression (emotion)"/
39. (depress* adj3 (adolescent* or infant* or child* or student* or anaclitic* or episode* or disorder or scale* or score* or symptom* or unipolar)).ti,ab,id.
40. ((depress* or mood* or mental or psychological or wellbeing or well being or emotion*) adj3 (improve* or onset or prevent* or reduc*)).ti,ab,id.
41. (depress* or dysthymi* or affective disorder* or affective symptom* or mood* or mental).ti,id.
42. ((axis 1 or axis I) adj disorder*).ti,ab,id.
43. exp anxiety/
44. anxiety disorders/ or acute stress disorder/ or death anxiety/ or generalized anxiety disorder/ or exp obsessive compulsive disorder/ or panic disorder/ or post-traumatic stress/ or exp posttraumatic stress disorder/ or separation anxiety disorder/
45. phobias/ or acrophobia/ or agoraphobia/ or claustrophobia/ or ophidiophobia/ or school phobia/ or social phobia/

46. fear/ or panic/ or panic attack/
47. (anxi* adj3 (adolescent* or child* or disorder* or general* or interpersonal or separation or social*)).ti,ab,id.
48. (phobi* or agoraphobi* or PTSD or post trauma* or posttrauma* or panic* or OCD or obsess* or compulsi* or GAD or stress disorder* or stress reaction* or acute stress or neurosis or neuroses or neurotic or psychoneuro* or (school adj3 (refusal or avoid*)) or social avoidance or mutism).ti,ab,id.
49. (((anxi* or fear or fright) adj3 (perform* or athlet* or music* or act* or test* or exam*)) or math* anxiety).ti,ab,id.
50. (public adj3 (speak* or speech)).ti,ab,id.
51. conduct disorder/ or explosive disorder/ or oppositional defiant disorder/
52. *behavior disorders/
53. exp juvenile delinquency/
54. exp antisocial behavior/
55. ((behavi* or conduct or personalit*) adj3 (agressi* or nonagressi* or antisocial or anti social or dyssocial or defiant or delinquen* or disturb* or disrupt* or disorder* or internalizing or externalizing or internalising or externalising or problem*)).ti,ab,id.
56. ((conduct or behavi* or antisocial or anti social or dyssocial or emotional* or internalizing or externalizing or internalising or externalising) adj3 (problem* of difficult* or psychopathol*)).ti,ab,id.
57. (oppositional adj3 (defiant* or disorder*)).ti,ab,id.
58. or/33-57
59. early intervention/
60. "onset (disorders)"/
61. health promotion/ or exp health education/ or health knowledge/ or health literacy/
62. mental health programs/
63. public health/
64. prevention/ or preventive medicine/
65. "3365".cc.
66. prevent*.ti,id.
67. prevention of.ab.
68. (prevent* adj3 (intervention or educat* or pilot or program* or project or protocol* or training or universal or targeted or primary or secondary or selective or indicated or study or trial)).ti,ab,id.
69. ((early or brief) adj3 intervention*).ti,ab,id.
70. ((universal or targeted) adj3 (program* or intervention*)).ti,ab,id.
71. (vulnerabl* or at risk or (risk adj3 reduc*)).ti,ab,id.
72. at risk populations/ or predisposition/ or risk factors/ or "susceptibility (disorders)"/
73. orphans/ or orphanages/
74. bullying/ or conflict/ or emotional abuse/ or school violence/ or teasing/ or threat/ or victimization/
75. school dropouts/
76. runaway behavior/
77. exp Crime Victims/
78. exp violent crime/
79. exp violence/
80. trauma/
81. rural environments/
82. urban environments/
83. exp neighborhoods/
84. exp social issues/
85. war/ or conflict/
86. accidents/ or exp disasters/
87. exp transportation accidents/

88. survivors/
89. bereavement/ or grief/
90. divorce/ or child custody/
91. parental death/ or exp parental absence/
92. life changes/
93. child abuse/ or abandonment/ or child neglect/
94. family conflict/ or domestic violence/ or emotional abuse/
95. (bereave* or bullying or divorce or foster care or grief or humanitarian or orphan* or RTA or refugee* or survivor* or victim* or war).ti,ab,id.
96. (stigma or help seeking).ti,ab,id,hw.
97. or/59-96
98. treatment effectiveness evaluation.sh.
99. clinical trials.sh.
100. mental health program evaluation.sh.
101. placebo.sh.
102. randomi#ed.ti,ab.
103. (random* adj3 (administ* or class* or control* or determine* or divide* or distribut* or expose* or fashion or number* or place* or recruit* or subsitut* or treat*)).ab.
104. RCT.ab,id.
105. (waitlist* or wait-list* or waiting-list* or wait* list* or (waiting adj (condition or control)) or WLC).ti,ab,id.
106. placebo.ti,ab,id.
107. at random.ab.
108. ((no intervention* or non intervention* or non-intervention* or without any intervention*) adj3 (control* or group*)).ti,ab,id.
109. (reference group or observation group or control group).ti,ab,id.
110. trial.ti.
111. or/98-110
112. (4 or (13 and 30)) and (31 or 58) and 97 and 111
113. (4 or (13 and 30)) and 32 and 111
114. 4 and 58 and 111
115. or/112-114

Embase
1 juvenile/ or exp child/ or exp adolescent/ or young adult/
2 (child* or boy* or girl* or kids or juvenil* or minors or paediatric* or pediatric* or adolesc* or preadolesc* or preadolesc* or pubert* or pubescen* or prepube* or prepube* or teen* or (young adj (adult* or people or patient* or men* or women* or male or female or survivor* or offender* or minorit*)) or youth* or student* or undergrad*).ti,ab,kw.
3 (child* or adolesc* or paediatr* or pediatr*).jn.
4 or/1-3
5 school/ or college/ or community college/ or high school/ or kindergarten/ or middle school/ or nursery school/ or primary school/ or university/
6 education/ or curriculum/ or education program/ or learning environment/ or exp special education/
7 school health service/
8 exp student/
9 (preschool or kindergarten or school* or college* or campus* or classroom* or curricul* or teacher or gatekeeper or pupil*).ti,ab,kw.
10 peer group/

11 ((peer or peers) adj (education or group or relation* or support* or intervention* or leader*)).ti,ab,kw.
12 student* union.ti,ab,kw.
13 ((church or communit* or holiday* or religi* or spiritual* or youth or vacation) adj2 (camp or club or group)).ti,ab,kw.
14 ((church or communit* or holiday* or religi* or spiritual* or youth or vacation) adj based).ti,ab,kw.
15 or/5-14
16 mental health/ or community mental health/ or psychological well being/
17 mental stress/ or *stress/
18 (mental health or mental* ill* or psychiatric).ti,kw.
19 (wellbeing or well being).ti,kw.
20 (stress* or distress*).ti,kw.
21 *wellbeing/
22 or/16-21
23 depression/ or dysthymia/ or *major depression/ or "mixed anxiety and depression"/
24 mood disorder/
25 mood/ or *emotion/
26 (depress* or dysthymi* or affective disorder* or affective symptom* or mood* or mental).ti.
27 (depress* adj2 (adolescent* or child* or anaclitic* or episode* or disorder or scale* or score* or symptom* or unipolar)).ti,ab,kw.
28 ((depress* or mood* or mental or psychological or wellbeing or well being or emotion*) adj2 (improve* or onset or prevent* or reduc*)).ti,ab,kw.
29 ((Axis 1 or Axis I) adj disorder*).ab.
30 or/23-29
31 *anxiety/
32 exp anxiety disorder/
33 anxi*.ti.
34 (anxi* adj3 (adolescent* or child* or disorder* or general* or interpersonal or separation or social*)).ti,ab,kw.
35 (phobi* or agoraphobi* or PTSD or post trauma* or posttrauma or panic* or OCD or obsess* or compulsi* or GAD or stress disorder* or stress reaction* or acute stress or neurosis or neuroses or neurotic or psychoneuro* or (school adj2 (refusal or avoid*)) or social avoidance or mutism).ti,ab,kw.
36 (((anxi* or fear or fright) adj3 (perform* or athlet* or music* or act* or test* or exam*)) or math* anxiety).ti,ab,kw.
37 (public adj3 (speak* or speech)).ti,ab,kw.
38 or/31-37
39 conduct disorder/
40 *behavior disorder/
41 psychosocial disorder/
42 juvenile delinquency/ or delinquency/
43 problem behavior/
44 *social adaptation/
45 ((behavi* or conduct or personalit*) adj2 (agressi* or nonagressi* or antisocial or anti social or dyssocial or defiant or delinquen* or disturb* or disrupt* or disorder* or internali#ing or externali#ing or problem*)).ti,ab,kw.
46 ((conduct or behavi* or antisocial or anti social or dyssocial or emotional* or internali#ing or externali#ing) adj3 (problem* of difficult* or psychopathol*)).ti,ab,kw.
47 (oppositional adj3 (defiant* or disorder*)).ti,ab,kw.
48 oppositional defiant disorder/

49 or/39-48
50 Prevention/ or Preventive Medicine/
51 Prophylaxis/
52 primary prevention/ or secondary prevention/
53 health promotion/ or health education/ or health literacy/
54 pc.fs.
55 prevent*.ti,kw.
56 prevention of.ab.
57 (prevent* adj2 (intervention or educat* or pilot or program* or project or protocol* or training or universal or targeted or primary or secondary or selective or indicated or study or trial)).ti,ab,kw.
58 ((early or brief) adj intervention*).ti,ab,kw.
59 ((universal or targeted) adj2 (program* or intervention*)).ti,ab,kw.
60 (vulnerabl* or at risk or (risk adj2 reduc*)).ti,ab,kw.
61 risk/ or risk factor/
62 risk of developing.ab.
63 exp "accidents and accident related phenomena"/
64 exp emotional deprivation/
65 exp grief/
66 social problem/ or exp abuse/ or bullying/ or exp crime/ or divorce/ or exp human rights abuse/ or exp social discrimination/ or exp social exclusion/ or exp violence/
67 orphaned child/
68 exp victim/
69 exp disaster/
70 life event/
71 coping behavior/ or runaway behavior/
72 "population and population related phenomena"/ or high risk population/ or minority group/ or rural population/ or urban population/ or vulnerable population/
73 exp survivor/
74 exp warfare/
75 conflict/ or family conflict/
76 early intervention/
77 or/50-76
78 randomized controlled trial/
79 (randomi#ed or randomi#ation).ab,ti,kw.
80 (RCT or (random* adj3 (administ* or allocat* or assign* or class* or cluster* or control* or determine* or divide* or distribut* or expose* or fashion or number* or place* or recruit* or subsitut* or treat*))).ab.
81 at random.ab.
82 trial.ti,kw.
83 or/78-82
84 4 and 15 and (22 or 30 or 38 or 49) and 77 and 83

Cochrane Central Register of Controlled Trials
#1 MeSH descriptor: [Child] explode all trees
#2 MeSH descriptor: [Adolescent] this term only
#3 MeSH descriptor: [Young Adult] this term only
#4 (child* or boy* or girl* or kids or juvenil* or minors or paediatric* or pediatric* or adolesc* or preadolesc* or pre-adolesc* or pubert* or pubescen* or prepube* or pre-pube* or teen* or (young next (adult* or people or patient* or men* or women* or male or female or survivor* or

offender* or minorit*)) or youth* or student* or undergrad*):ti,ab,kw (Word variations have been searched)
#5 child* or adolesc* or paediatr* or pediatr*:so
#6 (#1 or #2 or #3 or #4 or #5)
#7 MeSH descriptor: [Education] this term only
#8 MeSH descriptor: [Schools] this term only
#9 MeSH descriptor: [Schools, Nursery] this term only
#10 MeSH descriptor: [Students] this term only
#11 MeSH descriptor: [Universities] this term only
#12 (preschool or kindergarten or school* or college* or campus* or classroom* or curricul* or teacher or gatekeeper or pupil*):ti,ab,kw (Word variations have been searched)
#13 MeSH descriptor: [Peer Group] this term only
#14 ((peer or peers) next (education or group or relation* or support* or intervention* or leader*)):ti,ab,kw (Word variations have been searched)
#15 "student* union":ti,ab,kw (Word variations have been searched)
#16 ((church or communit* or holiday* or religi* or spiritual* or youth or vacation) near/3 (camp or club or group)):ti,ab,kw (Word variations have been searched)
#17 ((church or communit* or holiday* or religi* or spiritual* or youth or vacation) near/3 based):ti,ab,kw (Word variations have been searched)
#18 university or universities:ti,ab,kw (Word variations have been searched)
#19 (primary or secondary or tertiary) next educat*:ti,ab,kw (Word variations have been searched)
#20 (#7 or #8 or #9 or #10 or #11 or #12 or #13 or #14 or #15 or #16 or #17 or #18 or #19)
#21 MeSH descriptor: [Depression] this term only
#22 MeSH descriptor: [Depressive Disorder] this term only
#23 MeSH descriptor: [Mood Disorders] this term only
#24 depress* or dysthymi* or affective disorder* or affective symptom* or mood* or mental:ti (Word variations have been searched)
#25 depress* near/3 (adolescent* or child* or anaclitic* or episode* or disorder or scale* or score* or symptom* or unipolar):ti,ab,kw (Word variations have been searched)
#26 ((depress* or mood* or mental or psychological or wellbeing or well being or emotion*) near/3 (improve* or onset or prevent* or reduc*)):ti,ab,kw
#27 (axis 1 or axis I) next disorder*
#28 MeSH descriptor: [Anxiety Disorders] explode all trees
#29 MeSH descriptor: [Anxiety] this term only
#30 MeSH descriptor: [Performance Anxiety] this term only
#31 (anxi* near/3 (adolescent* or child* or disorder* or general* or interpersonal or separation or social*))
#32 (phobi* or agoraphobi* or PTSD or post trauma* or posttrauma or panic* or OCD or obsess* or compulsi* or GAD or stress disorder* or stress reaction* or acute stress or neurosis or neuroses or neurotic or psychoneuro* or (school near/3 (refusal or avoid*)) or social avoidance or mutism)
#33 (((anxi* or fear or fright) near/3 (perform* or athlet* or music* or act* or test* or exam*)) or math* anxiety)
#34 (public near/3 (speak* or speech))
#35 MeSH descriptor: [Conduct Disorder] this term only
#36 MeSH descriptor: [Child Behavior Disorders] this term only
#37 MeSH descriptor: [Juvenile Delinquency] this term only
#38 MeSH descriptor: [Social Behavior] this term only
#39 MeSH descriptor: [Social Behavior Disorders] explode all trees

#40 ((behavi* or conduct or personalit*) near/3 (agressi* or nonagressi* or antisocial or anti social or dyssocial or defiant or delinquen* or disturb* or disrupt* or disorder* or internalizing or externalizing or internalising or externalising or problem*))
#41 ((conduct or behavi* or antisocial or anti social or dyssocial or emotional* or internalizing or externalizing or internalising or externalising) adj3 (problem* of difficult* or psychopathol*))
#42 (oppositional near/3 (defiant* or disorder*))
#43 ((conduct disorder*) near/3 (onset or prevent*))
#44 MeSH descriptor: [Adaptation, Physiological] this term only
#45 MeSH descriptor: [Emotions] this term only
#46 MeSH descriptor: [Mental Health] this term only
#47 MeSH descriptor: [Social Adjustment] this term only
#48 MeSH descriptor: [Stress, Psychological] this term only
#49 (mental health or mental* ill* or psychiatric)
#50 (wellbeing or well-being or "well being")
#51 stress* or distress*
#52 (#21 or #22 or #23 or #24 or #25 or #26 or #27 or #28 or #29 or #30 or #31 or #32 or #33 or #34 or #35 or #36 or #37 or #38 or #39 or #40 or #41 or #42 or #43 or #44 or #45 or #46 or #47 or #48 or #49 or #50 or #51)
#53 (#6 and #20 and #52) [Population + Setting + Condition] (n=10686 Trials)
[Prevention/Risk Factors]
#54 MeSH descriptor: [Preventive Health Services] this term only
#55 MeSH descriptor: [Early Intervention (Education)] this term only
#56 MeSH descriptor: [Health Literacy] this term only
#57 MeSH descriptor: [Patient Education as Topic] this term only
#58 MeSH descriptor: [Health Promotion] this term only
#59 MeSH descriptor: [Primary Prevention] this term only
#60 MeSH descriptor: [Secondary Prevention] this term only
#61 prevent*:ti (Word variations have been searched)
#62 prevent*:kw (Word variations have been searched)
#63 "prevention of"
#64 (prevent* near/3 (intervention or educat* or pilot or program* or project or protocol* or training or universal or targeted or primary or secondary or selective or indicated or study or trial))
#65 ((early or brief) next intervention*)
#66 ((universal or targeted) near/3 (program* or intervention*))
#67 (vulnerabl* or "at risk" or (risk near/3 reduc*))
#68 MeSH descriptor: [Risk] explode all trees
#69 MeSH descriptor: [Accidents] explode all trees
#70 MeSH descriptor: [Bereavement] explode all trees
#71 MeSH descriptor: [Bullying] this term only
#72 MeSH descriptor: [Child of Impaired Parents] this term only
#73 MeSH descriptor: [Child, Orphaned] this term only
#74 MeSH descriptor: [Crime Victims] this term only
#75 MeSH descriptor: [Disasters] explode all trees
#76 MeSH descriptor: [Divorce] explode all trees
#77 MeSH descriptor: [Life Change Events] this term only
#78 MeSH descriptor: [Runaway Behavior] this term only
#79 MeSH descriptor: [Urban Population] this term only
#80 MeSH descriptor: [Rural Population] this term only
#81 MeSH descriptor: [Survivors] this term only
#82 MeSH descriptor: [Violence] explode all trees
#83 MeSH descriptor: [Warfare] explode all trees

#84 MeSH descriptor: [Family Conflict] this term only
#85 MeSH descriptor: [Psychosocial Deprivation] this term only
#86 MeSH descriptor: [Poverty] this term only
#87 (bereave* or bullying or divorce or foster care or grief or humanitarian or orphan* or RTA or refugee* or survivor* or victim* or war)
#88 (#54 or #55 or #56 or #57 or #58 or #59 or #60 or #61 or #62 or #63 or #64 or #65 or #66 or #67 or #68 or #69 or #70 or #71 or #72 or #73 or #74 or #75 or #76 or #77 or #78 or #79 or #80 or #81 or #82 or #83 or #84 or #85 or #86 or #87 or #87)
#89 #53 and #88 [Population + Setting + Condition + Prevention/Risk Factors] (n=3575)
#90 (#26 or #43) and #6 and #20 [(MH or Conduct Disorder Prevention) + Population + Setting] (n=1273)
#91 #89 or #90

Additional outcomes to be included in NIHR monograph

For transparency, additional outcomes were intervention acceptability, self-reported problem behaviours (e.g. substance use) and academic attainment, as defined by study authors. Parent-reported prevention or reduction of disorder-specific symptoms was also recorded. During our initial Patient and Public Involvement (PPI) focus groups, stigma was identified as important to young people and was included on this basis.

The report (NIHR 15/49/08) will be available from available from nihr.ac.uk in Spring 2020

Decision Rule for choosing between multiple-report scales

Decision Rule for Depression Scales

1. Scores that combine depression and other symptoms will be excluded. (e.g. scales which measure 'internalising symptoms' or combined anxiety and depression scores).
2. Choice between multiple scales
    a. Use self-reports in preference to clinician-rated scales.
    b. Use instruments with well-studied psychometric properties.
    c. Use inventories aimed at paediatric populations in preference to inventories aimed at general population.
    d. Use instruments specifically targeted to measure depressive symptoms in preference to instruments with a broader scope.
    e. Use most commonly reported scale across studies.

Decision Rule for Anxiety Scales

1. Scores that combine anxiety and other symptoms will be excluded. (e.g. total RCADS would be excluded as it is a combined depression and anxiety score. Whereas the RCADS total anxiety subscale would be included in preference)
2. Use total anxiety scores where available
    a. If total anxiety score is not available, but a generalised anxiety subscale is we will use the subscale (for universal populations we think most interventions are likely to be targeting non-specific anxiety and are not sure what the importance of separation and social anxiety are. And some other subscales e.g. PTSD, OCD are no longer considered anxiety disorders in DSM V)
3. Choice between multiple scales:
    a. Use inventories of general symptoms in preference to instruments targeting specific anxiety domains.
    b. If several inventories of general symptoms are available, use those aimed at general population in preference to instruments aimed at identifying patients with anxiety disorders.
    c. Use most commonly reported scale across studies

Intervention and control classifications applied in this study

**Intervention**	**Definition used in this study to categorise interventions**
Usual curriculum	Where an active intervention takes place during a regular timetabled class and participants in the control group continue to receive the regular class. This could be a range of different classes which could include 'wellbeing' or health lesson or a timetabled academic lesson.
Waiting List	Where participants (schools, parents or children) were explicitly told that they would receive the active intervention at a later date. Whilst participants are likely to also be receiving usual curriculum or no intervention control, the use of a waitlist design takes precedence in our categorisation.
No intervention	Where the active intervention was held outside of regular timetabled classes (e.g. after school) *and* the participants were not described as being in a wait list control
Attention control	Any *de novo* intervention provided to the participants for the purpose of the research study.
Psycho-support and counselling	A non-specific therapeutic intervention which might include listening, signposting to further services, forming an attachment or therapeutic alliance.
Psycho-education	A systematic approach to providing background information regarding the illness and/or the ways in which an intervention might help.
Cognitive behavioural	A group of allied techniques utilising a set of overlapping cognitive and behavioural techniques. Here, we took an inclusive approach, e.g. if an author labelled an intervention as Cognitive Behavioural (CBT) but only used a single component (e.g. cognitive restructuring) we categorised the intervention as CBT.
Behavioural	Behavioural Therapy (BT) is a group of allied techniques that focus on behavioural models of psychology and seek to modify maladaptive behaviours. Here this could include interventions based on behavioural activation, self-monitoring, role-playing, scheduling pleasant activities.
Third wave	Third wave therapies combine principles of CBT and principles of mindfulness, acceptance and flexibility. To be categorised as third wave, the intervention should focus on modifying the function of thoughts rather than on modifying their content.
Mindfulness/ Relaxation	A combined category. Relaxation includes breathing exercises, muscle relaxation and yoga from the Iyengar or Hatha traditions (as opposed to e.g. Vinyasa or Bikram traditions). Mindfulness interventions were included here if they focus on solely on meditation, or relaxation without aspects of traditional psychotherapeutic approaches (see Third wave for mindfulness-based CBT).
Interpersonal	Techniques which primarily focus on addressing the relationship between young people and significant adults (e.g., teachers, parents), with regards avoiding/ resolving conflict via improved communication skills.
Biofeedback	A mind-body intervention which uses instruments to learn to control physiological responses, such as heart rate.
Exercise	Cardiovascular interventions designed to raise heart rate and breathing into (at least) the moderate intensity level.
Bias/ cognitive modification	Post-hoc identified category: applied cognitive processing therapies often delivered via computer. Includes attention bias and interpretation bias training.

Study follow-up and timepoints (months from end of intervention)

Studies 1 to 76 are listed alphabetically for universal interventions. Studies 77 to 137 are listed alphabetically for targeted interventions. The reference numbers correspond to the study characteristics tables (on page 21) and the list of included studies on page 87 of this Appendix document.

Study	ref number	Timepoints	1-5	6-12	13-24	25+
Ahlen 2018	1	0	.	12	.	.
Anticich 2013	2	.	.	.	.	.
Araya 2013	3	.	3	12	.	.
Attwood 2012	4	0	.	.	.	.
Aune 2009	5	0	.	.	.	.
Baker 1984	6	0	.	.	.	.
Barrett 2001	7	0	.	.	.	.
Barrett 2005	8	.	.	.	.	.
Barry 2017	9	0	.	.	.	.
Bonhauser 2005	10	0	.	.	.	.
Bouchard 2013	11	0	.	.	.	.
Britton 2014	12	0	.	.	.	.
Burckhardt 2015	13	0	5	.	.	.
Burckhardt 2016	14	0	.	.	.	.
Calear 2009	15	0	.	6	.	.
Calear 2016	16	0	.	6, 12	.	.
Calear 2016b	17	0	3	.	.	.
Cardemil 2002	18	0	3	6	.	.
Chaplin 2006	19	0	.	.	.	.
Clarke 1993a	20	0	3	.	.	.
Clarke 1993b	21	0	3	.	.	.
Collins 2014	22	0	.	6	.	.
Dadds 2008	23	.	.	.	.	.
Eather 2016	24	.	.	.	.	.
Essau 2012	25	0	.	6, 12	.	.
Gallegos 2008	26	0	.	6	.	.
Gillham 1994	27	0	.	.	.	.
Gillham 2006	28	0	.	6, 12	.	.
Gillham 2007	29	0	.	6, 12	18, 24	36
Guhct 2017	30	0	.	12	.	.
Haden 2014	31	.	.	.	.	.
Hiebert 1989b	32	0	.	.	.	.
Hodas 2015	33	0	.	6	.	.
Horowitz 2007	34	0	.	6	.	.
Johnson 2016	35	0	3	.	.	.
Johnson 2017	36	0	.	6,12	.	.
Johnstone 2014	37	0	.	6	18	30, 42, 54
Khalsa 2012	38	0	.	.	.	.
Kindt 2014	39	0	.	6, 12	.	.

Lock 2003	40	0	.	12	.	.
Lowry-Webster 2001	41	0	.	12	.	.
Mendelson 2010	42	0	.	.	.	.
Merry 2004	43	0	.	6, 12	18	.
Miller 2010	44	0	.	.	.	.
Miller 2011a	45	0	.	6	.	.
Miller 2011c	46	0	.	12	.	.
Pahl 2010	47	.	.	.	.	.
Pattison 2001	48	0	.	8	.	.
Perry 2017	49	0	.	6	18	.
Pophillat 2016	50	0	.	.	.	.
Possel 2004	51	0	3	6	.	.
Possel 2008	52	0	.	6, 12	.	.
Possel 2013	53	0	4	8, 12	.	.
Potek 2012	54	0	.	.	.	.
Quayle 2001	55	0	.	6	.	.
Raes 2014	56	0	.	6	.	.
Reynolds 2011	57	0	.	6	.	.
Rivet-Duval 2011	58	0	.	6	.	.
Roberts 2003	59	0	.	6	18	30
Roberts 2010	60	0	.	6	18	
Roberts 2018	61	.	.	.	.	.
Rodgers 2015	62	0	4	.	.	.
Rooney 2006	63	0	.	9	18	
Rose 2014	64	0	.	6, 12	.	.
Ruttledge 2016	65	0	3	.	.	.
Sawyer 2010	66	.	.	.	18	30, 42, 54
Shatte 1997	67	0	4	8, 12	.	.
Sheffield 2006a	68	0	.	6, 12	.	.
Soffer 2003	69	0	1	.	.	.
Spence 2003	70	0	.	12	24	36, 48
Stallard 2012a	71	.	.	6, 12	.	.
Stallard 2014	72	.	.	12	24	.
Tak 2016	73	0	.	6,12	18, 24	.
Tomba 2010	74	0	.	6	.	.
Velásquez 2015	75	0	.	.	.	.
Wong 2014	76	0	.	.	.	.
Arnarson 2009	77	.	.	.	.	.
Balle 2010	78	0	.	6	.	.
Berry 2009	79	0	.	.	.	.
Clarke 1995	80	0	.	6, 12	.	.
Congelton 1995	81	0	.	.	.	.
Cooley-Strickland 2011	82	0	.	.	.	.
Cova 2011	83	0	.	.	.	.
Cowell 2009	84	0	.	9.5	.	.
Cui 2016	85	0	.	6	.	.
Dobson 2010	86	0	3	6	.	.
Ellis 2011	87	0	.	.	.	.

Fitzgerald 2016	88	0	3	.	.	.
Fung 2016	89	0	.	.	.	.
Gaete 2016	90	.	3	.	.	.
Gillham 2012	91	0	.	6	.	.
Hiebert 1989a	92	0	.	.	.	.
Higgins 2006	93	0	1	6, 12	.	.
Hunt 2009	94	0	.	.	24	48
Jaycox 1994	95	0	.	6, 12	18, 24	.
Jordans 2010	96	0	.	.	.	.
Kiselica 1994	97	0	3	.	.	.
Liddle 2010	98	0	.	.	.	.
Livheim 2014	99	0	.	.	.	.
Manassis 2010	100	0	.	12	.	.
McCarty 2011	101	0	.	6, 12	18	.
McCarty 2013	102	0	.	6, 12	.	.
McLaughlin 2011	103	0	.	.	.	.
McLoone 2012	104	0	.	12	.	.
Mifsud 2005	105	0	.	6	.	.
Miller 2011b	106	0	3	12	.	.
Noël 2013	107	0	.	.	.	.
Owen 1982	108	0	.	.	.	.
Peden 2000	109	0	.	6	18	.
Peng 2015	110	0	.	.	.	.
Poppelaars 2016	111	0	3	6, 12	.	.
Puskar 2003	112	0	.	6, 12	.	.
Rice 2008	113	0	2	.	.	.
Rohde 2014	114	0	.	6, 12	18, 24	.
Scholten 2016	115	0	3	.	.	.
Schoneveld 2016	116	0	3	.	.	.
Schoneveld 2018	117	0	3	6	.	.
Seligman 1999	118	0	3	.	.	36
Seligman 2007	119	0	1,3	.	.	.
Sheffield 2006b	120	0	.	6, 12	.	.
Simpson 2008	121	0	.	.	.	.
Siu 2007	122	0	.	.	.	.
Sportel 2013	123	0	.	6, 12	.	.
Stallard 2012b	124	.	.	6, 12	.	.
Stice 2006	125	0	1	6	.	.
Stice 2008	126	0	.	6, 12	24	.
Stoppelbein 2003	127	0	.	6	.	.
Takagaki 2016	128	0	.	.	.	.
Tokolahi 2018	129	0	.	.	.	.
Topper 2017	130	0	3	12	.	.
van Starrenburg 2017	131	0	3	.	.	.
Wijnhoven 2014	132	0	1	6	.	.
Woods 2011	133	0	2	12	.	.
Young 2006	134	0	3	6	.	.
Young 2010	135	0	.	6, 12	18	.
Young 2016	136	0	.	6	.	.
Yu 2002	137	0	3	6	.	.

Further statistical details and OpenBUGS code used for network meta-analysis

In this paper we conducted a network meta-analysis. In a three-intervention network, an indirect estimate of the relative effect of intervention B compared with C (d^I_{BC}) can be formed by comparing direct, head-to-head trials of A vs C (d^D_{AC}) to A vs B trials (d^D_{AB}), such that $d^I_{BC} = d^D_{AC} - d^D_{AB}$. To be valid, this requires that the A vs C studies do not differ from the A vs B studies, on average, in factors that might interact with the intervention effects. This is known as the consistency or transitivity assumption. An alternative way of expressing this is through 'joint randomisability'[1], in the respect that all three interventions could be included in a (hypothetical) multi-arm trial. The first step in evaluating this assumption is analogous to the consideration of clinical homogeneity prior to a standard meta-analysis. Here we extracted study level characteristics that could be potential effect modifiers and visually examined their similarity across all trials in the network[2]. Characteristics evaluated included population (universal or targeted), setting, baseline symptom severity, gender, socio-economic variables and participant age. The characteristics of studies tables are reported on page 24 of this appendix document. Note, if there is a loop of evidence (i.e. both and indirect and direct estimate of d_{BC})), the consistency assumption can be formally evaluated using statistical measures, as we describe below.

Random effects NMA were conducted for the main outcomes of interest. Analyses were conducted within a Bayesian framework, implemented using OpenBUGS.[3] Both fixed and random effects models were fitted. Heterogeneity was assessed by examining the posterior median between-study standard deviation (τ) and 95% Credible Intervals (CrIs) from the random effects model, and by comparing model fit of the fixed and random effects models. Model fit was measured by the posterior mean of residual deviance.[4] In addition, we examined the Deviance Information Criterion (DIC), which penalises model fit with model complexity.[5] Differences of ≥ 5 points for posterior mean residual deviance and DIC were considered meaningful, with lower values preferred.[5] Inconsistency was assessed by comparing the goodness of fit of a model assuming consistency with one allowing for inconsistency. Vague prior distributions were specified for baseline effects on reference arm and intervention effect parameters (N(0, 0.0001)), and for the between study heterogeneity parameter (U(0,5)).[4] The robustness of the between study heterogeneity parameter was assessed using a U(0,10) prior. Results were unchanged. Convergence was assessed using the Brooks–Gelman–Rubin diagnostic and was satisfactory by 100,000 simulations for all outcomes. Model fit and convergence details are reported on Page 21 of this appendix.

References:

1. Salanti G. Indirect and mixed-treatment comparison, network, or multiple-treatments meta-analysis: many names, many benefits, many concerns for the next generation evidence synthesis tool. Research Synthesis Methods 2012; 3(2): 80-97
2. Chaimani A Caldwell, DM, Li T, Higgins JPT, Salanti G. . Chapter 11: Undertaking network meta-analyses. In: Higgins JPT TJ, Chandler J, Cumpston M, Li T, Page MJ, Welch VA (editors), ed. Cochrane Handbook for Systematic Reviews of Interventions. 2nd Edition ed. Chichester (UK): John Wiley & Sons; 2019: 285–320.
3. http://www.openbugs.net. Accessed 11-08-19
4. Dias S. Network Meta-Analysis for Decision Making; Wiley, 2018.
5. Spiegelhalter DJ, Best NG, Carlin BP, Van Der Linde A. Bayesian measures of model complexity and fit. Journal of the Royal Statistical Society: Series B (Statistical Methodology) 2002; 64(4): 583-639.

The following OpenBUGS code adapted from: S Dias, NJ Welton, A Sutton, AE Ades NICE DSU technical support document 2: a generalised linear modelling framework for pairwise and network meta-analysis of randomised controlled trials. Full details are available from:
http://nicedsu.org.uk/wp-content/uploads/2017/05/TSD2-General-meta-analysis-corrected-2Sep2016v2.pdf (2016)

Standardised mean differences were calculated using Hedge's *g*. J is the Hedge's *g* adjustment. To standardise we used trial-specific pooled baseline standard deviations (pooled.sd).

```
model{

for(i in 1:ns){

w[i,1] <- 0
delta[i,1] <- 0
mu[i] ~ dnorm(0,.0001)
      for (k in 1:na[i]) {
      var[i,k] <- pow(se[i,k],2)
            prec[i,k] <- 1/var[i,k]
            y[i,k] ~ dnorm(phi[i,k],prec[i,k])
            phi[i,k]<-theta[i,k]*(pooled.sd[i]/J[i])
            theta[i,k] <- mu[i] + delta[i,k]
```

```
      Dnum[i,k] <- (y[i,k]-phi[i,k])*(y[i,k]-phi[i,k])
    dev[i,k] <- Dnum[i,k]/var[i,k]

}

resdev[i] <- sum(dev[i,1:na[i]])

for (k in 2:na[i]) {
delta[i,k] ~ dnorm(md[i,k],taud[i,k])
md[i,k] <- d[t[i,k]] - d[t[i,1]] + sw[i,k]
taud[i,k] <- tau *2*(k-1)/k
w[i,k] <- (delta[i,k] - d[t[i,k]] + d[t[i,1]])
sw[i,k] <- sum(w[i,1:k-1])/(k-1)
} }

totresdev <- sum(resdev[])
d[1]<-0

for (k in 2:nt){ d[k] ~ dnorm(0,.0001) }
sd ~ dunif(0,5)
tau <- pow(sd,-2)

for (k in 1:nt) {
      rk[k] <- rank(d[],k)
      best[k] <- equals(rk[k],1)
            for (h in 1:nt){
                  prob[h,k] <- equals(rk[k],h) }
} }
```

list(nt=5, ns=11)

t[,1]	y[,1]	se[,1]	t[,2]	y[,2]	se[,2]	t[,3]	y[,3]	se[,3]	pooled.sd[]	J[]	na[]
3	-0.24	0.032348	5	-0.24	0.03347	NA	NA	NA	0.415088	0.995633	2
2	-0.05	0.038875	3	-0.13	0.03707	NA	NA	NA	0.365477	0.994307	2
1	-0.06	0.041548	3	-0.13	0.034557	NA	NA	NA	0.350819	0.99435	2
1	-4.9	0.58321	3	-5	0.670354	NA	NA	NA	4.99911	0.991736	2
1	2.9	3.640692	3	-10.1	3.374514	NA	NA	NA	26.56373	0.991549	2
2	-3.78	1.42514	3	-5.86	1.873442	NA	NA	NA	17.1976	0.995984	2
2	-7.5	1.023118	3	-6.5	0.950584	NA	NA	NA	8.882437	0.994854	2

Model fit, heterogeneity and convergence details: self-report depression

Table reports model fit statistics for each population, setting and timepoint analysis. Model fit for depression and anxiety outcomes reported in separate tables

Timepoints: PI: post-intervention, short: 0-5 months, Mid: 6 to 12 months, Long: 12 to 24 months, Longer: >25 months.
Model: FE = fixed effect, RE = random effects. Cons: consistency model. Incon: inconsistency model
Datapoints: number of effective datapoints in model
Totresdev: posterior mean total residual deviance
DIC: deviance information criterion
PD: effective parameters
SD: between study heterogeneity parameter (95% Credible Intervals)
Convergence: number of iterations before convergence occurred, on 3 chains observed using BGR diagnostic in OpenBUGS.

Population	Setting	Time	Model	Datapoints	Totresdev	DIC	PD	SD	Convergence
**Universal**	**Primary**	PI	FE Cons		69.4	126.5	17.0	-	15,000
		PI	RE Cons	29.0	29.0	95.2	26.1	0.32 (0.18, 0.59)	30,000
		PI	RE Incon		28.9	94.8	25.8	0.28 (0.16, 0.52)	20,000
		Short	FE Cons						
		Short	RE Cons	2 studies - not connected					
		Short	RE Incon						
		Mid	FE Cons		43.4	68.7	13.0	-	40,000
		Mid	RE Cons	21.0	24.7	56.6	19.5	0.29 (0.08, 0.73)	10,000
		Mid	RE Incon		25.8	57.5	19.4	0.25 (0.06, 0.70)	20,000
		Long	FE Cons		6.5	15.4	5.0	-	50,000
		Long	RE Cons	7.0	6.6	16.8	6.3	0.11 (0.00, 1.80)	
		Long	RE Incon		6.6	16.9	6.4	0.10 (0.00, 1.70)	
		Longer	FE Cons						
		Longer	RE Cons	1 study					
		Longer	RE Incon						
**Universal**	**Secondary**	PI	FE Cons		118.5	188.1	42.0	-	15,000
		PI	RE Cons	75	76.2	167.2	60.4	0.12 (0.07, 0.19)	30,000
		PI	RE Incon		82.6	170.7	60.5	0.11 (0.04, 0.19)	20,000
		Short	FE Cons		19.0	44.2	15.0	-	20,000
		Short	RE Cons	18	17.7	44.8	16.9	0.12 (0.01, 0.80)	51,000
		Short	RE Incon		17.9	45.9	17.7	0.18 (0.01, 2.29)	70,000
		Mid	FE Cons		82.8	141.0	34.0	-	10,000
		Mid	RE Cons	62	65.9	135.4	45.3	0.08 (0.02, 0.15)	25,000
		Mid	RE Incon		66.3	135.2	44.7	0.06 (0.01, 0.13)	25,000
		Long	FE Cons		12.2	36.3	11.0	-	50,000
		Long	RE Cons	15	12.8	38.1	12.2	0.05 (0.00, 0.30)	15,000
		Long	RE Incon		12.8	38.2	12.3	0.06 (0.00, 0.30)	20,000
		Longer	FE Cons		6.7	17.9	6.0	-	20,000
		Longer	RE Cons	7.0	6.9	18.9	6.9	0.51 (0.01, 4.47)	100,000
		Longer	RE Incon		6.9	18.9	6.9	0.51 (0.01, 4.45)	20,000
**Targeted**	**Primary**	PI	FE Cons		15.5	41.2	8.0	-	10,000
		PI	RE Cons	10.0	10.3	38.1	10.1	0.60 (0.08, 3.80)	60,000
		PI	RE Incon		10.3	38.0	10.0	0.60 (0.07, 3.79)	100,000
		Short	FE Cons						
		Short	RE Cons	No studies					
		Short	RE Incon						

		Mid	FE Cons		4.0	14.2	4.0	-	30,000
		Mid	RE Cons	4.0	4.0	14.2	4.0	2.51 (0.13, 4.87)	60,000
		Mid	RE Incon		4.0	14.2	4.0	2.48 (0.13, 4.88)	40,000
		Long	FE Cons	No studies					
		Long	RE Cons						
		Long	RE Incon						
		Longer	FE Cons	1 study					
		Longer	RE Cons						
		Longer	RE Incon						
**Targeted**	**Secondary**	PI	FE Cons					-	
		PI	RE Cons	56.0	57.0	180.8	51.1	0.38 (0.25, 0.58)	80,000
		PI	RE Incon		58.1	182.2	51.3	0.37 (0.24, 0.59)	50,000
		Short	FE Cons					-	
		Short	RE Cons	17.0	17.6	65.2	16.1	0.44 (0.15, 1.12)	50,000
		Short	RE Incon		18.3	66.1	16.2	0.41 (0.11, 1.10)	50,000
		Mid	FE Cons					-	
		Mid	RE Cons	45.0	45.3	160.5	40.4	0.44 (0.28, 0.69)	50,000
		Mid	RE Incon		46.1	162.4	41.4	0.43 (0.28, 0.69)	40,000
		Long	FE Cons					-	
		Long	RE Cons	12.0	11.5	42.9	11.4	0.80 (0.14, 4.16)	100,000
		Long	RE Incon		11.4	42.9	11.4	0.66 (0.11, 4.04)	50,000
		Longer	FE Cons	1 study					
		Longer	RE Cons						
		Longer	RE Incon						

Model fit, heterogeneity and convergence: self-report anxiety

Population	Setting	Time	Model	Datapoints	Totresdev	DIC	PD	SD	Convergence
**Universal**	**Primary**	PI	FE Cons		52.26	137.80	19.00	-	20,000
		PI	RE Cons	34	37.99	132.00	27.40	0.13 (0.02, 0.28)	40,000
		PI	RE Incon		40.57	135.30	28.10	0.12 (0.01, 0.29)	30,000
		Short	FE Cons	1 study					
		Short	RE Cons						
		Short	RE Incon						
		Mid	FE Cons		42.53	100.9	14.0	-	20,000
		Mid	RE Cons	24	24.42	89.9	21.1	0.21 (0.08, 0.44)	60,000
		Mid	RE Incon		25.71	91.87	21.8	0.20 (0.06, 0.46)	20,000
		Long	FE Cons		5.76	15.91	5.0	-	30,000
		Long	RE Cons	7	6.34	17.64	6.2	0.13 (0.01, 2.05)	150,000
		Long	RE Incon		6.31	17.63	6.2	0.11 (0.00, 1.88)	130,000
		Longer	FE Cons	1 study					
		Longer	RE Cons						
		Longer	RE Incon						
**Universal**	**Secondary**	PI	FE Cons		92.88	112.8	27.0	-	40,000
		PI	RE Cons	45	48.25	81.09	39.2	0.16 (0.09, 0.28)	30,000
		PI	RE Incon		48.94	81.75	39.7	0.15 (0.08, 0.27)	30,000

		Short	FE Cons		14.63	17.9	7.0	-	20,000
		Short	RE Cons	8	8.04	12.34	8.0	1.32 (0.17, 4.68)	30,000
		Short	RE Incon		8.09	12.44	8.1	1.30 (0.17, 4.68)	40,000
		Mid	FE Cons		53.25	69.01	21.0	-	20,000
		Mid	RE Cons	33	40.52	62.85	27.6	0.11 (0.03, 0.27)	20,000
		Mid	RE Incon		41.2	63.5	27.6	0.10 (0.02, 0.26)	30,000
		Long	FE Cons		5.61	15	5.0	-	10,000
		Long	RE Cons	6	5.86	16.07	5.8	0.61 (0.02, 4.50)	60,000
		Long	RE Incon		5.88	16.09	5.9	0.61 (0.02, 4.50)	80,000
		Longer	FE Cons						
		Longer	RE Cons	1 trial					
		Longer	RE Incon						
		PI	FE Cons		53.3	83.8	15.0	-	150,000
		PI	RE Cons	23	23.9	61.5	22.2	0.42 (0.21, 0.89)	60,000
		PI	RE Incon		24.0	61.8	22.3	0.43 (0.21, 0.91)	50,000
		Short	FE Cons		7.6	-8.6	7.0		20,000
		Short	RE Cons	8	7.9	-7.5	7.9	0.64 (0.02, 4.51)	80,000
		Short	RE Incon		7.9	-7.5	7.8	0.68 (0.02, 4.53)	50,000
		Mid	FE Cons		23.7	49.3	8.0	-	
**Targeted**	**Primary**	Mid	RE Cons	11	11.3	39.9	11.0	0.52 (0.15, 2.53)	50,000
		Mid	RE Incon		11.5	40.2	11.1	0.53 (0.14, 2.56)	100,000
		Long	FE Cons						
		Long	RE Cons	No studies					
		Long	RE Incon						
		Longer	FE Cons						
		Longer	RE Cons	No studies					
		Longer	RE Incon						
		PI	FE Cons		38.01	103.7	22.99	-	50,000
		PI	RE Cons	36	36.25	105.1	26.16	0.06 (0.00, 0.21)	70,000
		PI	RE Incon		37.66	109.4	29.02	0.06 (0.00, 0.23)	50,000
		Short	FE Cons		13.98	41.41	13.98	-	30,000
		Short	RE Cons	14	14.02	41.48	14.02	2.50 (0.14, 4.87)	30,000
		Short	RE Incon			41.44	14	2.53 (0.15, 4.88)	50,000
		Mid	FE Cons		13.63	51.81	9.986	-	30,000
**Targeted**	**Secondary**	Mid	RE Cons	16	13.7	53.57	11.68	0.06 (0.00, 0.25)	60,000
		Mid	RE Incon		13.73	53.55	11.62	0.05 (0.00, 0.21)	50,000
		Long	FE Cons						
		Long	RE Cons	1 study					
		Long	RE Incon						
		Longer	FE Cons						
		Longer	RE Cons	1 study					
		Longer	RE Incon						

Study characteristics: Universal interventions

Study	Ref	Design	Target	Setting	Age	Cou ntry	Control	Int 1	Int2	Int3	No. sessions	Intensity (mins)	Delivered by	Format	Format2
Ahlen 2018	1	C	A+D	Primary	8-11	HIC	UC	CBT			10	600	Teacher	F2F	Group
Anticich 2013	2	C	A	Primary	4-7	HIC	WL	PS	CBT		10	NR	Teacher	F2F	Group
Araya 2013	3	C	D	Secondary	14.5	MIC*	UC	CBT			11	660	Psychologist	F2F	Group
Attwood 2012	4	I	A	Primary	10-12	HIC	AC	CBT			6	270	Researcher	MM	Group/ Individual
Aune 2009	5	C	A	Secondary	10-15	HIC	NI	CBT			3	135	Psychologist	F2F	Group
Baker 1984	6	C	A	Secondary	16-18	HIC	CBT SH	CBT			8	360	Teacher	F2F	Group
Barrett 2001	7	C	A	Primary	10-12	HIC	UC	CBT	CBT		10	750	Teachers or Psychologist	F2F	Group
Barrett 2005	8	C	A	Secondary	9-16	HIC	NI	CBT			10	525	Psychologist	F2F	Group
Barry 2017	9	I	D	Secondary	15-16	HIC	UC	CBT			4	NC	"Coach"	F2F	Group
Bonhauser 2005	10	C	A+D	Secondary	15.3	MIC	Exercise	Exercise			120	10800	Teacher	F2F	Group
Bouchard 2013	11	I	A	Primary	9-12	HIC	WL	CBT			10	750	Psychologist	F2F	Group
Britton 2014	12	I	A	Secondary	11.79	HIC	AC	M/R			30	225	Teacher	F2F	Group
Burckhardt 2015	13	C	A+D	Secondary	14-16	HIC	AC	M/R			6	360	NA	MM	Group
Burckhardt 2016	14	C	A+D	Secondary	15-18	HIC	UC	3RD			16	480	Psychologist	F2F	Group
Calear 2009	15	C	A+D	Secondary	12-17	HIC	WL	CBT			5	150	Teacher	MM	Group
Calear 2016	16	C	A	Secondary	12-18	HIC	WL	CBT	CBT		6	210	Teacher or MHP supported	MM	Group
Calear 2016b	17	C	A	Secondary	13-17	HIC	WL	CBT			6	210	Teacher	MM	Group
Cardemil 2002	18	I	D	Primary	10-12	HIC	UC	CBT			12	1080	Psychologist	F2F	Group
Chaplin 2006	19	I	D	Secondary	11-14	HIC	NI	CBT	CBT		12	1080	Teacher & Researchers	F2F	Group
Clarke 1993a	20	C	D	Secondary	14-16	HIC	UC	PE			3	150	Teacher	F2F	NA
Clarke 1993b	21	C	D	Secondary	14-16	HIC	UC	BT			5	250	Teacher	F2F	NA
Collins 2014	22	C	A	Primary	9-10	HIC	UC	CBT			10	NR	Teacher or school counsellor	F2F	Group
Dadds 2008	23	C	A	Primary	3-7	HIC	NI	CBT			6	NR	Psychologist	F2F	Group
Eather 2016	24	C	A+D	Secondary	15-16	HIC	WL	Exercise			16	960	Fitness instructor	F2F	Group
Essau 2012	25	C	A	Primary	9-12	HIC	WL	CBT			10	600	Psychologist	F2F	Group
Gallegos 2008	26	C	A+D	Primary	9-11	MIC	UC	CBT			10	600	Teacher	F2F	Group
Gillham 1994	27	I	D	Primary	10-12	HIC	NI	CBT			12	1440	Psychologist	F2F	Group

Gillham 2006	28	I	A+D	Secondary	11-13	HIC	NI	CBT			8	720	Researchers & Psychologist	F2F	Group
Gillham 2007	29	I	D	Secondary	11-14	HIC	NI	AC + PS	CBT		12	1080	Teachers & school counsellors & psychologists	F2F	Group
Guhct 2017	30	C	A+D	Secondary	14-21	HIC	UC	3RD			4	480	Teacher	F2F	Group
Haden 2014	31	I	A+D	Primary	10-11	HIC	UC	M/R			36	3240	Teacher	F2F	Group
Hiebert 1989b	32	I	A	Secondary	13-14	HIC	AC	M/R			11	660	Teacher & school counsellor	F2F	Group
Hodas 2015	33	I	A+D	Secondary	12-14	HIC	WL	CBT			7	455	Psychologist	F2F	Group
Horowitz 2007	34	I	D	Secondary	14-15	HIC	UC	IPT	CBT		8	720	Psychologist	F2F	Group
Johnson 2016	35	C	A+D	Secondary	13.63	HIC	UC	3RD			9	495	Psychologist	F2F	Group
Johnson 2017	36	C	A+D	Secondary	13.44	HIC	UC	3RD	3RD		9	450	Psychologist	F2F	Group
Johnstone 2014	37	C	A+D	Primary	9-10	HIC	UC	CBT			10	600	Teacher	F2F	Group
Khalsa 2012	38	C	A+D	Secondary	15-19	HIC	UC	M/R			27.5	825	Yoga trainer	F2F	Group
Kindt 2014	39	C	D	Secondary	11-16	HIC	UC	CBT			16	NR	Teacher	F2F	Group
Lock 2003	40	C	A	Secondary	NR	HIC	NI	CBT			10	750	Teacher	F2F	Group
Lowry-Webster 2001	41	C	A+D	Secondary	10-13	HIC	WL	CBT			10	600	Teacher	F2F	Group
Mendelson 2010	42	C	D	Primary	9-11	HIC	WL	M/R			48	2160	Yoga trainer	F2F	Group
Merry 2004	43	I	D	Secondary	13-15	HIC	AC	CBT+IPT			11	NR	Teacher	F2F	Group
Miller 2010	44	C	A	Primary	7-12	HIC	WL	CBT			NR	NR	Teacher	F2F	Group
Miller 2011a	45	C	A	Primary	7-13	HIC	WL	CBT			9	NR	Teacher & school counsellor	F2F	Group
Miller 2011c	46	C	A	Primary	7-13	HIC	AC	CBT			9	540	Teacher & school counsellor	F2F	Group
Pahl 2010	47	C	A	Primary	4-6	HIC	WL	CBT			9	270	Psychologist	F2F	Group
Pattison 2001	48	I	D	Primary	9-12	HIC	NI	AC	CBT	CBT	10	1200	Child mental health professionals.	F2F	Group
Perry 2017	49	C	D	Secondary	16-17	HIC	AC	CBT			7	175	NA	MM	Group
Pophillat 2016	50	C	A+D	Primary	6-8	HIC	UC	CBT			10	NR	Teacher	F2F	Group
Possel 2004	51	C	D	Secondary	13-14	HIC	UC	CBT			10	900	Psychologist or graduate students	F2F	Group
Possel 2008	52	C	D	Secondary	12-13	HIC	UC	CBT			10	900	Psychologist or graduate students	F2F	Group
Possel 2013	53	C	D	Secondary	14-16	HIC	UC	AC	CBT		10	900	Psychologist or graduate students	F2F	Group
Potek 2012	54	I	A	Secondary	14-17	HIC	WL	M/R			6	270	Psychologist	F2F	Group
Quayle 2001	55	I	D	Primary	11-12	HIC	WL	CBT			8	640	Psychologist	F2F	Group
Raes 2014	56	C	D	Secondary	13-20	HIC	UC	3RD			8	800	Psychologist	F2F	Group
Reynolds 2011	57	C	D	University	17.9	HIC	UC	BT			14	1680	Psychologist	F2F	Group
Rivet-Duval 2011	58	I	D	Secondary	12-16	MIC	WL	CBT+IPT			11	660	Teacher	F2F	Group

Roberts 2003	59	C	D	Secondary	11-13	HIC	UC	CBT			12	NR	Psychologist	F2F	Group
Roberts 2010	60	C	A+D	Secondary	11-13	HIC	UC	CBT			20	1200	Teacher	F2F	Group
Roberts 2018	61	C	A+D	Primary	9-12	HIC	UC	CBT	CBT		20	1200	Teacher	F2F	Group
Rodgers 2015	62	I	A	Secondary	12-13	HIC	WL	CBT			10	600	Psychologist	F2F	Group
Rooney 2006	63	C	D	Primary	8-9	HIC	NI	CBT			8	480	Psychologist	F2F	Group
Rose 2014	64	C	D	Secondary	9-14	HIC	WL	CBT+IPT	CBT+ IPT		11	495	Psychologist	F2F	Group
Ruttledge 2016	65	C	A	Primary	9-13	HIC	WL	CBT			10	NR	Teacher	F2F	Group
Sawyer 2010	66	C	D	Secondary	13.1	HIC	UC	CBT			30	900	Teacher	F2F	Group
Shatte 1997	67	I	D	Secondary	12-14	HIC	NI	AC	CBT		12	1440	Teachers & Psychologist	F2F	Group
Sheffield 2006a	68	C	D	Secondary	13-15	HIC	NI	CBT			8	380	Teachers & Psychologist	F2F	Group
Soffer 2003	69	I	D	Primary	10-11	HIC	NI	AC	BT		8	320	Psychologist	F2F	Group
Spence 2003	70	C	D	Secondary	12-14	HIC	UC	CBT			8	380	Teacher	F2F	Group
Stallard 2012a	71	C	D	Secondary	12-16	HIC	UC	AC	CBT+ IPT		9	495	Facilitator	F2F	Group
Stallard 2014	72	C	A	Primary	9-10	HIC	UC	CBT	CBT		9	540	Teacher & Facilitator	F2F	Group
Tak 2016	73	C	D	Secondary	12-14	HIC	UC	CBT			16	800	Teacher & Psychologist	F2F	Group
Tomba 2010	74	C	A+D	Secondary	11.41	HIC	CBT	CBT			6	720	Psychologist	F2F	Group
Velásquez 2015	75	I	A+D	Primary/ Secondary	NR	MIC	WL	M/R			24	2880	Yoga trainer	F2F	Group
Wong 2014	76	C	A+D	Secondary	14-16	HIC	UC	CBT	CBT		6	240	Teacher	MM	Group

*Study conducted in Chile. At the time of the trial Chile was considered to be a middle-income country. https://datahelpdesk.worldbank.org/knowledgebase/articles/906519-world-bank-country-and-lending-groups

Key: C: cluster randomised trial, I: individual randomised trial, D:Depression, A:Anxiety, A+D: anxiety and depression,

HIC: high income country, MIC: middle-income country, LIC: low-income country

UC: usual curriculum, WL: wait list, NI: no intervention, AC: attention control, CBT: cognitive behavioural therapy, IPT: interpersonal therapy, CBT + IPT: cognitive behavioural therapy+ interpersonal therapy, PS: psycho-support, BM: bias modification, 3rd: third wave, M/R: mindfulness/relaxation based interventions, BIO: biofeedback, OT: occupational therapy, BT: behavioural therapy, SH: self-help

F2F: face to face, MM: multi-media/computer-based

Study characteristics: Targeted interventions (selective and indicated)

Study	Ref	Design	N	Target	Type	Setting	Age	Country	Control	Int1	Int2	Int3	No sessions	Intensity	Delivered by	Format1	Format2
Arnarson 2009	77	I	171	D	Ind	Secondary	14-15	HIC	NI	CBT + IPT			14	NR	Psychologist	F2F	Group
Balle 2010	78	I	92	A	Sel	Secondary	11-17	HIC	WL	CBT			6	270	Psychologist	F2F	Group
Berry 2009	79	C	46	A	Ind	Secondary	12-15	HIC	WL	CBT			8	480	Psychologist	F2F	Group
Clarke 1995	80	I	150	D	Ind	Secondary	14-16	HIC	NI	CBT			15	675	School psychologist	F2F	Group
Congelton 1995	81	I	15	D	Sel	Secondary	12-14	HIC	WL	CBT			8	480	Psychologist	F2F	Group
Cooley-Strickland 2011	82	I	98	A	Ind	Primary	9-10	HIC	WL	CBT			13	780	Psychologist	F2F	Group
Cova 2011	83	I	209	D	Ind	Secondary	14-15	MIC*	NI	CBT			11	990	Psychologist	F2F	Group
Cowell 2009	84	C	302	D	Sel	Primary	10.4	HIC	NI	PS			6	NR	Nurse	F2F	Group
Cui 2016	85	I	180	D	Ind	University	19.42	MIC	WL	CBT	PS		8	960	Psychologist	F2F	Group
Dobson 2010	86	I	49	A+D	Ind	Secondary	13-18	HIC	AC	CBT			15	675	Psychologist	F2F	Group
Ellis 2011	87	I	39	D	Ind	University	18-25	HIC	NI	CBT	PS		3	300	NA	MM	Individual
Fitzgerald 2016	88	I	100	A	Ind	Secondary	15-18	HIC	AC	BM			4	NR	Researcher	MM	Group
Fung 2016	89	I	19	A+D	Ind	Secondary	12-14	HIC	WL	3RD			12	720	Psychologist	F2F	Group
Gaete 2016	90	I	342	D	Ind	Secondary	13-18	MIC*	UC	CBT			8	360	Psychologist	F2F	Group
Gillham 2012	91	I	408	D	Ind	Secondary	10-15	HIC	NI	CBT	CBT		10	900	Teacher and school counsellor	F2F	Group
Hiebert 1989a	92	I	38	A	Ind	Secondary	15-17	HIC	WL	M/R	BIO		8	320	Psychologist	F2F	Individual
Higgins 2006	93	I	78	A	Ind	University	17-19	HIC	NI	CBT			2	240	Psychologist	F2F	Group
Hunt 2009	94	C	260	A	Ind	Secondary	11-13	HIC	NI	CBT			10	500	Teacher and school counsellor	F2F	Group
Jaycox 1994	95	C	143	D	Ind	Primary	10-13	HIC	WL	CBT			12	1080	Psychologist	F2F	Group
Jordans 2010	96	C	325	A+D	Sel	Secondary	11-14	LIC	WL	MIXED			15	900	Researcher	F2F	Group
Kiselica 1994	97	I	48	A	Ind	Secondary	14-15	HIC	PE	CBT			8	480	Counsellors	F2F	Group
Liddle 2010	98	I	58	A	Sel	Primary/ Secondary	8-14	HIC	WL	CBT			10	NR	Psychologist	F2F	Group
Livheim 2014	99	I	58	D	Ind	Secondary	12-17	HIC	PS	3RD			8	NR	Psychologist	F2F	Group
Manassis 2010	100	I	148	A+D	Ind	Primary	8-11	HIC	AC	CBT			12	720	Psychologist	F2F	Group
McCarty 2011	101	I	67	D	Ind	Secondary	13	HIC	UC	CBT			12	NR	Not clear	F2F	Group

McCarty 2013	102	I	120	D	Ind	Secondary	11-15	HIC	PS	CBT			12	600	Therapists	F2F	Group
McLaughlin 2011	103	I	13	D	Ind	Primary/ Secondary	10-15	HIC	PS	CBT			10	500	Psychologist	F2F	Group
McLoone 2012	104	I	152	A	Ind	Primary	7-10	HIC	WL	CBT	CBT		10	600	School counsellors	F2F	Group
Mifsud 2005	105	C	91	A	Ind	Primary	8-11	HIC	WL	CBT			8	480	School counsellors	F2F	Group
Miller 2011b	106	C	191	A	Ind	Primary	7-12	HIC	AC	CBT			9	540	Teacher and school counsellor	F2F	Group
Noël 2013	107	I	32	D	Ind	Secondary	13-15	HIC	WL	CBT			12		Students	F2F	Group
Owen 1982	108	I	NR	A	Ind	Secondary	15-16	HIC	WL	M/R	CBT	CBT	6	180	Counsellors	F2F	Group
Peden 2000	109	I	NR	D	Ind	University	18-24	HIC	NI	CBT			NR	NR	NA	F2F	Group
Peng 2015	110	C	121	A+D	Sel	Secondary	14.2	MIC	NI	EX			24	NR	NR	F2F	Group
Poppelaars 2016	111	C	208	D	Ind	Secondary	11-16	HIC	WL	CBT	CBT	CBT	8	480	Psychologist	F2F	Individual
Puskar 2003	112	I	89	D	Ind	Secondary	14-18	HIC	NI	CBT			10	450	Nurse	F2F	Group
Rice 2008	113	I	28	A	Ind	Secondary	10-18	HIC	AC	CBT	M/R		16	560	Psychologist	F2F	Group
Rohde 2014	114	I	378	D	Ind	Secondary	13-19	HIC	PE	CBT	CBT SH		6	360	Psychologist or self-help	F2F	Group
Scholten 2016	115	I	138	A	Ind	Secondary	11-15	HIC	AC	BIO			6	360	Researcher	MM	Individual
Schoneveld 2016	116	I	136	A	Ind	Primary	8-13	HIC	AC	BIO			5	300	Researcher	MM	Group
Schoneveld 2018	117	I	174	A	Ind	Primary	7-12	HIC	CBT	BIO			6	360	Masters students and psychologist	MM	Group
Seligman 1999	118	I	225	A+D	Sel	University	19	HIC	NI	CBT			8	960	Psychologist	F2F	Group/ individual
Seligman 2007	119	I	240	A+D	Sel	University	19	HIC	NI	CBT			8	960	Psychologist	F2F/MM	Group
Sheffield 2006b	120	C		D	Ind	Secondary	13-15	HIC	NI	CBT	CBT	CBT	8	380	Teachers or school counsellor or both	F2F	Group
Simpson 2008	121	I	66	A+D	Ind	Primary	7-11	HIC	AC	CBT			12	1080	NR	F2F	Group
Siu 2007	122	I	47	A+D	Ind	Primary	7-10	HIC	WL	CBT			8	NR	Counsellors	F2F	Group
Sportel 2013	123	C	240	A	Ind	Secondary	12-15	HIC	NI	BM	CBT		20	900	NA	MM	Individual
Stallard 2012b	124	C	106 4	D	Ind	Secondary	12-16	HIC	UC	UC	CBT+ IPT		9	495	'Facilitator'	F2F	Group
Stice 2006	125	I	225	D	Ind	Secondary/ University	15-22	HIC	WL	CBT			4	240	Psychologist	F2F	Group
Stice 2008	126	I	341	D	Ind	Secondary	14-19	HIC	NI	CBT SH	PS	CBT	6	360	Self-help or Psychologist	F2F	Group
Stoppelbein 2003	127	C	59	D	Ind	Secondary	15	HIC	AC	CBT			10	500	Psychologist	F2F	Group

Takagaki 2016	128	I	118	D	Ind	University	18-19	HIC	NI	BT			5	300	Psychologist	F2F	Group
Tokolahi 2018	129	C	151	A+D	Sel	Primary	7-12	HIC	WL	OT			8	480	Occupational Therapist	F2F	Group
Topper 2017	130	I	167	A+D	Sel	Secondary	15-22	HIC	WL	CBT			6	540	Psychologist	F2F	Group
van Starrenburg 2017	131	I	141	A	Ind	Primary	7-13	HIC	WL	CBT			12	720	Psychologist	F2F	Group
Wijnhoven 2014	132	I	118	D	Ind	Secondary	11-15	HIC	WL	CBT			8	400	Therapist	F2F	Group
Woods 2011	133	I	56	D	Ind	Secondary	14	HIC	UC	CBT			8	720	School counsellors	F2F	Group
Young 2006	134	I	41	D	Ind	Secondary	11-16	HIC	PS	CBT			10	900	Psychologist/ Social worker	F2F	Group/individual
Young 2010	135	I	57	D	Ind	Secondary	13-17	HIC	PS	IPT			10	900	Psychologist	F2F	Group/ individual
Young 2016	136	I	186	D	Ind	Secondary	13.42	HIC	PS	IPT			11	450	Psychologist	F2F	Group/ individual
Yu 2002	137	I	220	D	Ind	Primary /Secondary	8-15	MIC	NI	CBT			10	1200	Teacher	F2F	Group

*Study conducted in Chile. At the time of the trial Chile was considered to be a middle-income country.
https://datahelpdesk.worldbank.org/knowledgebase/articles/906519-world-bank-country-and-lending-groups

Key: C: cluster randomised trial, I: individual randomised trial, D:Depression, A:Anxiety, A+D: anxiety and depression, Sel: selective intervention, Ind: indicated intervention

HIC: high income country, MIC: middle-income country, LIC: low-income country

UC: usual curriculum, WL: wait list, NI: no intervention, AC: attention control, CBT: cognitive behavioural therapy, IPT: interpersonal therapy, CBT + IPT: cognitive behavioural therapy+ interpersonal therapy, PS: psycho-support, BM: bias modification, 3rd: third wave, M/R: mindfulness/relaxation based interventions, BIO: biofeedback, OT: occupational therapy, BT: behavioural therapy, SH: self-help

F2F: face to face, MM: multi-media/computer-based

Attendance and Fidelity

The following tables report student attendance figures and facilitator fidelity to intervention, as reported by trial author.

Ref	Trial	Attendance
1	**Ahlen 2018**	School class medians of non-attendance ranged between 4.2 and 6.1% between classes'
3	**Araya 2013**	80.5% of students attended at least 6 sessions'
10	**Bonhauser 2005**	Eighty-seven percent (104 out of 120 sessions) of the designed sessions were completed.' page 118
13	**Burckhardt 2015**	Adherence was defined as the frequency with which students returned their completed workbook sections (ranging from 0-6): of 336 participants, 27 (8.0%) did not return any of their workbook sections, 52 participants (15.5%) returned 1-2 workbooks, 70 participants (20.8%) returned 3-4 workbooks, and 187 (55.6%) returned 5-6 workbooks' page 7
15	**Calear 2009**	The mean number of modules completed was 3.16 (SD 1.68), with 347 of the 559 (62%) intervention condition participants completing three or more modules of the MoodGYM program and 183 participants (32.7%) completing all five modules' page 1028
16	**Calear 2016**	Within the GAD school method condition, 78% of participants completed the first two weeks of the e-couch Anxiety and Worry program, while 43% completed at least four weeks of the program and 36%completed all six weeks of the intervention. In the e-GAD health service method, 87% of participants completed the first two weeks of the intervention, 65% completed at least four weeks of the e-couch Anxiety and Worry program and 50% completed all six weeks, with significantly greater program completion than the e-GAD school method' page 215
17	**Calear 2016b**	45% completed all six weeks of the intervention
19	**Chaplin 2006**	Girls in girls PRP attended a greater number of sessions than did girls in co-ed PRP, $t(61) = 2.04$, $p < .05$. Girls in girls groups attended an average of 7.03 sessions (SD = 4.15), whereas girls in co-ed groups attended an average of 5.04 sessions (SD = 3.56).
80	**Clarke 1995**	Attendance in the intervention groups averaged 72% (SO = 22%), with a range of 13% to 100%.
81	**Congelton 1995**	All subjects, with the exception of one, attended at least 75% of the sessions, 69% had perfect attendance during eight sessions. Data from one subject with 25% attendance was not included, which resulted in a total of 15 subjects in the data analysis' Page 55
82	**Cooley-Strickland 2011**	Including the make-up sessions, all participants attended at least 12 of the 13 sessions
83	**Cova 2011**	The mean sessions attended were 8.86 sessions for the universal modality of the program. A 76.5% of the participants attended 8 sessions or more. Only 4 participants attended less than 6 sessions (3.4%). In the indicated modality of the program, the mean sessions attended were 6 sessions. A 43% attended less than 6 sessions (A 8.9% of the participants did not attend any session).
84	**Cowell 2009**	'Children in the north side neighborhood received an average of eight classes and children on the south side neighborhood received an average of 4.72 classes ($t = -2.47$, $df = 109$, $p = 0.02$).

85	**Cui 2016**	Attendance was similar across groups: 56% of CB participants attended all eight sessions, compared with 53% of SG participants; 85% of CB participants and 82% of SG participants attended at least six of the eight sessions' Page 299
24	**Eather 2016**	Process evaluation results showed that all 16 session (60 min, 2 per week 8 weeks) were delivered with an attendance rate of 94%.' Page 18
25	**Essau 2012**	Children were allowed to miss a maximum of three sessions; however, they were required to complete an individual session with their trainer before they could join the subsequent group session. A total of 21 children missed one group session, 14 missed two group sessions, and another 6 missed three group sessions; all these children received an individual session before joining the next group session. Thus, effectively, all the children participated in all of the FRIENDS sessions. Parents of these children were also invited to participate in four parent evening sessions. About half (54%) of the parents, mostly mothers, participated in these sessions. Almost all parents who did not participate reported lacking the time due to a busy lifestyle as the main reason for their nonparticipation. Of those who participated, 20.6, 30.3, 31.5, and 17.6% finished one, two, three, and four group sessions, respectively. The modal number of sessions attended was three.' Page 454
89	**Fung 2016**	Youth on average attended 10.28 (85.63 %) out of 12 sessions among the intent-to-treat sample and 10.90 (90.86 %) sessions among the completers.' Pages 823-824
90	**Gaete 2016**	The average attendance rate per session was 55.5 % of the participants (SD = 5.9; range, 45.0–66.4 %)' Page 5
28	**Gillham 2006**	On average, parents of the 22 students assigned to the PRP condition attended 3.8 (or 63%) of the parent sessions. Parents of 20 (91%) students attended at least one session. Parents of nine students (41%) attended at least five of the six sessions. ''On average, the 22 students assigned to the PRP condition attended 5.5 (or 69%) of the eight PRP–CA sessions. Three students (14%), including the two students who dropped, attended two or fewer sessions. Ten students (45%) attended at least seven of the eight sessions' Page 338
29	**Gillham 2007**	On average, the 232 children assigned to PRP attended 6.71 (SD = 4.22) sessions, and the 231 children assigned to PEP attended 7.11 (SD = 4.43) sessions, t(461) = −1.01, ns. Thirty-seven children (16%) assigned to PRP and 35 (15%) assigned to PEP did not attend any sessions' Page 6
91	**Gillham 2012**	84% of students attended at least one session' '77% of students had parents who attended at least one session.' 'About half (44%) of the students attended the 5-month booster sessions' 'Parents of 27% of the students in PRP-AP attended the first parent booster session and parents of 21% of students in PRP-AP attended the second parent booster session' Page 7
35	**Johnson 2016**	With 87% attending at least six of the eight lessons' Page 6

36	**Johnson 2017**	Attendance at the pre-course information night for parents was low (8%), varying according to SES group (high, 29%; medium, 6% and low, 0%). Similarly, return rates of post course feedback forms were low (8%) with varying responses amongst SES brackets (high, 17%; medium, 7% and low, 4%). Given the low numbers of feedback forms returned, we used an alternative measure of the parental uptake of the weekly information i.e., the number of hits on the private YouTube channel per individual weekly lesson, interpreting one hit as one family/parent logging on. For the first two lessons, involvement was relatively high (40%) but dropped to 9% by the end of the course'. Page 40
37	**Johnstone 2014**	'Attendance rates indicated that each student completed an average of nine sessions (M = 9.03, SD = 2.143).
38	**Khalsa 2012**	the percentage of available sessions attended was 73.4% (SD=0.2%). Yoga session attendance was just over 80% at the beginning of the yoga program and declined to just under 70% by the end.' Page 84
99	**Livheim 2015**	Students that received the ACT intervention participated on average 5.8 sessions (same for boys and girls) out of 8 sessions.
101	**McCarty 2011**	One hundred percent of the parents of PTA youth received at least some of the parent intervention, and 94% received at least three of the four sessions.' Page 5
102	**McCarty 2013**	A total of 56 parents participated in home visits, with 85% (n=49) attending both home visit sessions. A total of 22 parents (38%) attended both parent workshop sessions, while 13 (22%) attended one session, and 23 parents (40%) did not attend any.' Page 6
42	**Mendelson 2010**	73.5% of students at one intervention school completed at least 75% of the intervention classes, with most absences the result of students missing school on that day. By contrast, slightly under 40% of students attended three quarters of the class sessions at the other intervention school. While school absence contributed to those missed classes, teacher focus group data indicated that some teachers at that school had prevented students from attending the intervention classes as a punishment for poor behavior in class.' Page 989
105	**Mifsud 2005**	'Children in the intervention attended a mean of 7.38 of the eight sessions (mean = Mean is missing. SD = 0.58). In contrast, parents averaged less than one session of the two offered (mean = 0.8, SD = 0.61)
49	**Perry 2017**	Indeed, the completion rate of 4 or more modules in the lifeSTYLE group (which did not encounter the same degree of difficulty due to a smaller load on IT systems) was substantially higher (88%).' Page 8
11	**Poppelaars 2016**	'Participants in the OVK and OVK&SPARX conditions who completed the program were present at an average of 6.77 (SD 1.17) out of 8 lessons with a minimum of 4 lessons received by all participants.
53	**Possel 2013**	Sessions attended M (SD): Cognitive behavioural prevention program: 8.5 (2.3); non-specific control: 8.6 (2.0)
112	**Puskar 2003**	'Students attended an average of nine sessions'

55	**Quayle 2001**	49% of the program content. Two students attended seven sessions (8.3%) and five students attended five or more sessions (21%). The rest of the students attended three or four sessions.' Page 198
59	**Roberts 2003**	Attendance rates were high, 87% to 99% over the 12 sessions. No child missed more than 2 sessions. Four children left the intervention group during the program, and 6 children could not be contacted at postintervention.' Page 623
60	**Roberts 2010**	Student attendance checklists indicated that only 5.2% of students were absent for more than 25% of the SLS lessons, while 31.4% of students missed up to 25% of the modules. Nine percent of students were absent for more than 25% of the OTS lessons, while 46.6% of the students missed up to 25% of the modules' Page 71
114	**Rohde 2014**	Participants in the CB group condition attended an average of 5.3 sessions (SD = 0.9); 48% attended all 6 sessions and none attended less than 3 sessions; 94% received an individual make-up session if they missed a session. The average number of make-up sessions in CB group participants was 0.7 (SD = 0.9).' Page 8
116	**Schoneveld 2016**	Most children attended all five game sessions (n ¼ 110; 80.9%); mean number of game sessions was 4.71 (SD ¼ 0.69). In most cases, both parents participated in the study (n ¼ 100).' Page 325
118	**Seligman 1999**	Attendance at the workshop averaged about 85%' Not page
119	**Seligman 2007**	Attendance at the workshop averaged 84%.' Page 1116
68	**Sheffield 2006a (Universal)**	Student attendance for the universal program was extremely high because the intervention was conducted within the school curriculum, with mean number of sessions attended in excess of 90%.' Page 76
120	**Sheffield 2006b (Indicated)**	With the indicated intervention, attendance records showed a mean attendance rate of 75% of the sessions.' Page 76
122	**Siu 2007**	Only 8 students missed one of their sessions for once'
123	**Sportel 2013**	A proportion of participants in the CBM condition (n = 16) did not start the CBM training, mostly due to technical difficulties.' Page 4
71	**Stallard 2012**	For all participants, the median percentage of sessions attended was 89% (quartiles 67–100) in the classroom-based CBT group and 100% (quartiles 88–100) in the attention control PSHE group, with 80% of those in the classroom-based CBT group and 95% of the attention control PSHE groups attending at least 60% of sessions. One year group (n = 199) was withdrawn from classroom-based CBT after four sessions because of school closures in adverse weather. When this year group was removed from analysis, the median percentage of classroom-based CBT sessions attended was 89% (quartiles 78–100), with 92.2% attending at least 60% of sessions.' Page 15

72	**Stallard 2014**	The percentage of participants attending more than 60% of sessions was 80% and 93% in the classroom-based CBT and attention control PSHE groups, respectively. Details were not collected on PSHE attendance in the usual PSHE arm.' Page 15
126	**Stice 2008**	Attendance was similar across groups, 44% of CB participants attended all 6 sessions compared to 45% of supportive-expressive participants; 86% percent of CB participants and 89% of supportive-expressive participants attended at least 3 of the 6 sessions.' Page 8
73	**Tak 2016**	Of the adolescents participating in the study, 67.8 % indicated that they were present at the booster session.' Page 954
128	**Takagaki 2016**	Participants could attend this program approximately once a week when they had time, because of the flexible time schedule. Each session was attended by up to three participants (49.4 % of sessions were conducted with one participant, 48.7 % with two participants, and 2.0 % with three participants).' Page 1173. '98.4 % of the participants completed all five treatment sessions. The adherence for homework assignment was also very high at 96.3 %.' Page 1175
130	**Topper 2017**	Participants in the group intervention attended significantly more sessions (M 4.59; SD 1.43) than starters in the internet condition (M 3.96; SD 1.65), p 0.02'
75	**Velásquez 2015**	'children who participated in the intervention were grouped into two categories: low-attendance (1 to 16 of the 24 sessions, n = 21) and high-attendance (17 or more of the 24 sessions, n = 47).'
134	**Young 2006**	One adolescent dropped out of treatment prior to the first group session but completed all assessments. Among the remaining 26 adolescents in IPT-AST, youth attended an average of 2.0 pre-group sessions (SD ¼ .2) and 6.9 group sessions (SD ¼ 1.0). The range was 1–2 pre-group sessions and 5–8 group sessions over 10– 12 weeks. One adolescent in IPT-AST attended a dropin community mental health center during the course of the intervention.' Page 1285
135	**Young 2010**	Attendance data were calculated for all adolescents assigned to intervention condition (including three adolescents who dropped out of IPTAST before the first group and one SC adolescent who left the school before she could begin counselling). IPT AST adolescents attended an average of 1.94 pre-group sessions (SD50.33) and 5.22 group sessions (SD5 2.55). School Counselling adolescents attended an average of 3.76 sessions (SD52.53).' 'One adolescent in School counselling dropped out of the study prior to receiving any treatment. Among the remaining 13 adolescents, participants had an average of 4.2 sessions (SD ¼ 2.2), with a range of 0–7 sessions over 10–12 weeks.' Page 430
136	**Young 2016**	Three adolescents in each condition attended no group sessions; all of them attended at least one individual session. On average, IPT-AST youth attended 6.80 (SD=1.85) group sessions and GC youth attended 6.18 (SD=1.85) group sessions, t(183)= −2.28, p<0.05. IPT-AST youth attended significantly more pre-group (M=2.00 vs. M=0.90), mid-group (M=0.98 vs. M=0.63), and booster sessions (M=3.64 vs. M=2.78) than GC youth.' Page 320

Facilitator fidelity/ integrity

Ref	Trial	Facilitator fidelity/ integrity
1	**Ahlen 2018**	During the intervention period, the first author regularly e-mailed and visited teachers at school to make sure they adhered to the intervention schedule. Seventeen teachers conducted all ten sessions in the program, two teachers only performed eight sessions, and one teacher six sessions. Unfortunately, only three teachers recorded sessions satisfactorily.
5	**Aune 2009**	The mean levels of adherence and competence were 5.33 and 5.67, respectively, which are very good to excellent ratings.
7	**Barrett 2001**	88-92% concordance
8	**Barrett 2005**	Group leaders were required to complete a checklist indicating compliance with the manual content for each session. 89% concordance between session and manual content for each session
14	**Burckhardt 2016**	Only ACT intervention was assessed. The adherence scale was scored on a 4-point Likert scale where 1 = minimal; 2 = satisfactory; 3=high; and 4=very high. The mean across all components was 3.0 (high)
21	**Clarke 1993b**	Compliance ratings ranged from 61% to 100% compliance, with a mean compliance rating of 86.2% across all 34 rated sessions, suggesting that teachers followed the structured intervention manual to a satisfactory degree'
80	**Clarke 1995**	'Protocol adherence for a tot al of 21 rated sessions was very high, averaging 93.9% compli ance (SO = 5.2) with a range of 77 .8% to 100%.'
22	**Collins 2014**	Mean fidelity ratings across facilitator groups by lesson: lesson 1, 6.63; lesson 2, 6.5; lesson 3, 5.88; lesson 4, 6.63; lesson 5, 6.38; lesson 6, 6.63; lesson 7, 6.63; lesson 8, 5.75; lesson 9, 5.88; lesson 10, 6.14
23	**Dadds 2008**	'Mean adherence by the facilitator to the manual was 96% averaged across the six sessions, with a range of 83–100%.'
86	**Dobson 2010**	Adherence was determined blindly, by whether the session could be identified in its correct condition and specific session or not. 'A total of 24 sessions were rated (3 sessions/ group and 4 groups/condition), and assignment to condition was 100%. In fact, the exact session was identified in almost every instance, suggesting strong adherence to treatment protocols'
25	**Essau 2012**	'Adherence to the intervention content ranged from 78 to 97%.'
26	**Gallegos 2008**	Average fidelity with group leader's skills: Classrooms 1 to 16=2.86; 2.36; 1.45; 1.57; 1.25; 1.56; 1.81; 2.28; 2.25; 1.61; 1.61; 1.75; 1.29; 1.71; 1.52; 1.52; 2.57; 1.62. Table 3 Page 76
29	**Gillham 2007**	'Average integrity scores for PRP groups were 4.9 (SD = 0.48) for degree covered and 80% (SD = 7.5%) for percentage covered. Average scores for PEP groups were 4.4 (SD = 0.36) for degree covered and 68% (SD = 5.7%) for percentage covered. Integrity scores were higher for PRP than PEP groups, likely reflecting the increased structure of the PRP. curriculum. Mean integrity scores for PRP and PEP groups did not differ significantly by school.'

91	**Gillham 2012**	On average, group leaders covered 68% of the integrity items to some degree (rated >= 2) and 47% of the items satisfactorily (rated >= 4)
94	**Hunt 2009**	'The majority of aims were rated as having been met extremely well (49.0% of ratings) or moderately well (44.8% of ratings). Forty per cent of schools provided rateable audiotapes, which showed that 55% of session aims were rated as having being met either moderately or extremely well.
36	**Johnson 2017**	'A score out of six was given for each of the three domains assessed, together with an overall average score for each lesson (Supplementary Table S1), with an average in the Proficient Band (5/6) across lessons.'
37	**Johnstone 2014**	88.46% of the teachers completed the checklist logbook and the mean percentage of the contents covered were 97,99,98, 96, 98,96,94,94,92,and 92 (M = 95.60%, SD 5.31%).
39	**Kindt 2014**	'Of the 28 teachers who provided the OVK lessons, 16 filled out adherence reports. On average 80.5% of 16 lessons were taught per class, with 95.3% of the first eight lessons and 65.5% of the last eight lessons being taught.'
102	**McCarty 2013**	'Coders achieved 'achieving 96% agreement' 'Overall mean intervention adherence across group leaders was excellent at 92%, (range 73% −100%). A comparable percent of audio-recorded ISP interviews (n = 10, 16%) were rated for adherence to core concepts of the intervention, with 99% agreement between coders. Individual adherence ratings ranged from 80% to 96%, with an overall mean of 91%.'
45	**Miller 2011a**	adherence to intervention content and objectives ranged from 96.4% (Session 3) to 83.3% (Session 6).
106	**Miller 2011b**	Adherence, using a Likert-scaled checklist of program objectives, ranged from 76.85% (Study 2) to 79.51% (Study1).' Page 317
46	**Miller 2011c**	Adherence, using a Likert-scaled checklist of program objectives, ranged from 76.85% (Study 2) to 79.51% (Study1).' Page 317
47	**Pahl 2010**	'Mean adherence by the facilitators to the manual was 94% (range = 90–98%) averaged across the nine sessions, across the two facilitators and the eight classrooms' Page 20
53	**Possel 2013**	'Group leaders' manual adherence was 91.6% in the CB condition (M 1.83, SD 0.09, range 1.60 –2.00) and 92.4% in the NSp condition (M 1.85, SD 0.12, range 1.51–2.00 [2 100% adherence]).'
59	**Roberts 2003**	The mean percentage of content covered as reported by facilitators for the 12 sessions was, 73, 81, 92, 97, 94, 41, 97, 96, and 93 (M 74.11%). With only one exception, facilitators achieved a high level of program integrity.' Page 623
60	**Roberts 2010**	The mean percentage of content covered for the Social Life Skills (SLS) lessons reported by teachers and cross checked against student workbooks ranged from 87.3% to 98.3% (M¼ 95.3%). For the Optimistic Thinking Skills (OTS) lessons, mean percentage of content covered per lesson was 97.5–100% (M ¼ 98.04%).
61	**Roberts 2018**	In 2003, 61 teachers in the training only condition implemented an average of 9.16 SLS modules (SD = 2.02) and 54 teachers in the training/coaching condition implemented an average of 9.24 modules (SD = 1.74). In 2004, 52 teachers in the training only condition implemented an average of 7.92 OTS modules (SD = 3.25) and 48 teachers in the training/coaching condition implemented an average of 8.06 modules (SD = 3.56).'
62	**Rodgers 2015**	'The protocol integrity checks showed concordance between session and manual content (89%)'

114	**Rohde 2014**	Regarding treatment adherence and facilitator competence, mean adherence was 7.0 (SD = 0.7) and mean competence was 7.1 (SD = 0.7) on the 1–10 point scales, which suggest that on average all key concepts of the various session sections were presented with good or very good therapist competence.
64	**Rose 2014**	No deviations from the manualized programs were observed'
65	**Ruttledge 2016**	'All teachers returned the fidelity checklist confirming that they had delivered all 10 sessions of the programme in sequence and covered the key components.'
66	**Sawyer 2010**	'Records of sessional completion were returned by 36% of teachers in Year 8, 41% in Year 9 and 44% in Year 10. Over the course of the programme teachers reported that they completed a mean of 70% of activities in Year 8 (range 17–100%), mean of 70% in Year 9 (range 21–100%) and mean of 74% in Year 10 (range 20–100%).
68	**Sheffield 2006a (Universal)**	Data from the Queensland teachers showed the mean number of program elements completed each session to be 85%
120	**Sheffield 2006b (Indicated)**	a mean number of program elements per session in excess of 92%.
69	**Soffer 2003**	All sessions met 100% adherence to the treatment manuals according to the scores by the two independent raters' Page 92
70	**Spence 2003**	'All teachers reported full implementation of Sessions 1, 2, 6, 7, and 8. However, around half the teachers did not have time to complete all the tasks set in Sessions 3, 4, and 5.'
71	**Stallard 2012**	'Of the 36 classroom-based CBT sessions observed to assess intervention fidelity, 31 covered all the core tasks, with at least 75% of core tasks being covered in the remaining five sessions.
72	**Stallard 2014**	All specified core tasks and home activities were delivered in the 24 health-led sessions assessed. In the 25 school-led sessions, 15 (60%) delivered all of the core tasks and the home activity, eight (32%) delivered all except the home activity and the remaining two (8%) did not deliver one core task and the home activity
126	**Stice 2008**	'With regard to fidelity, 96% of the CB components and 100% of the supportive-expressive components were rated as full adherence. With regard to therapist competence, 94% of the items in the CB sessions were rated at good competence (5% at partial and 1% at poor) and 94% of the items in the supportive-expressive sessions were rated at good competence (6% at partial, 0% at poor).
73	**Tak 2016**	Program fidelity was 80 %'
128	**Takagaki 2016**	'the therapist's adherence to the protocol was 100 %.'
130	**Topper 2017**	On average, 93% of the essential and required elements of the protocol were completed per session.'
136	**Young 2016**	'Across all sessions coded, 98.5 % of the techniques were delivered with fidelity and were given a rating of satisfactory (49.0%) or superior (49.5 %) for technique delivery. A global competency rating was given to each group leader at the end of the group: 8.8 % of the leaders received a rating of satisfactory, 41.2 % good, and 50.0 % excellent.'

Setting and population specific network plots: immediately post-intervention

K = no of studies contributing to each network

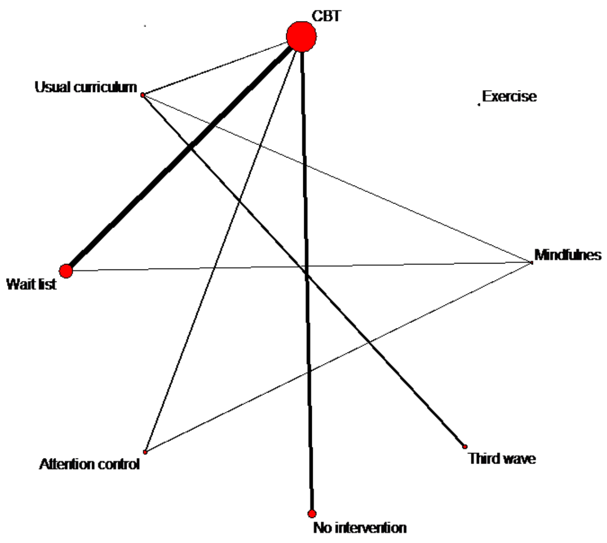

Universal Secondary Anxiety (k= 21)

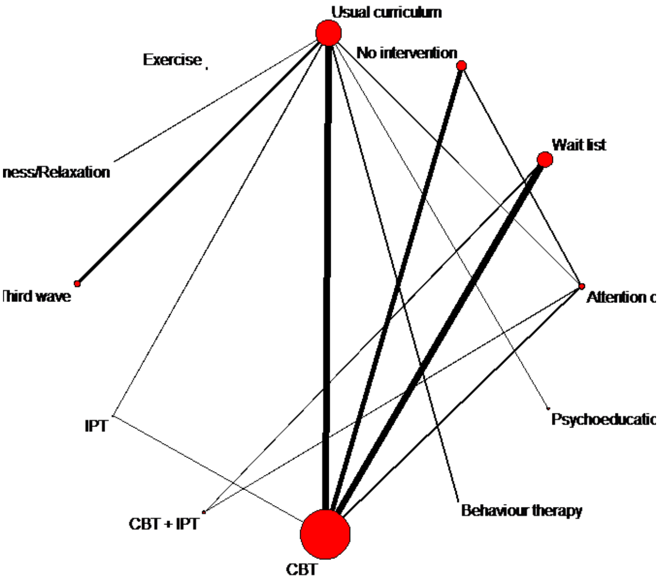

Universal Secondary Depression (k=34)

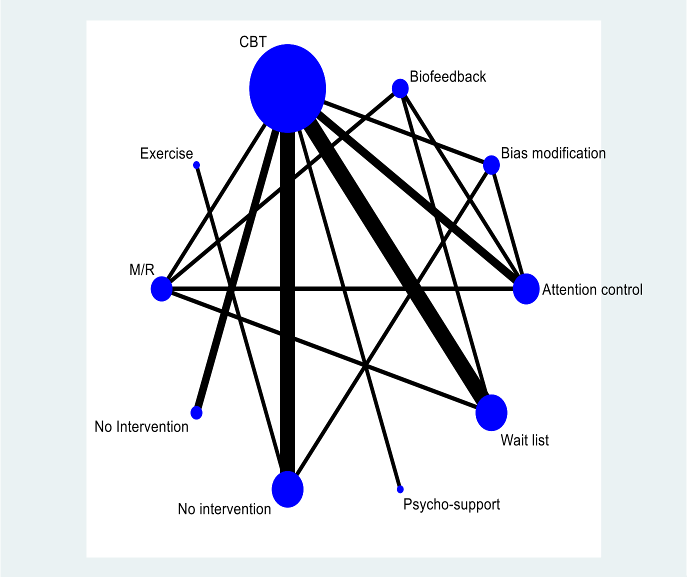

Targeted Secondary Anxiety (k= 15)

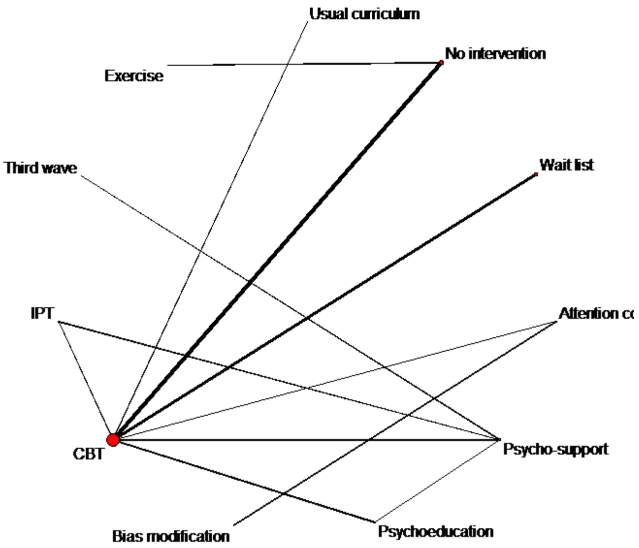

Targeted Secondary Depression (k= 24)

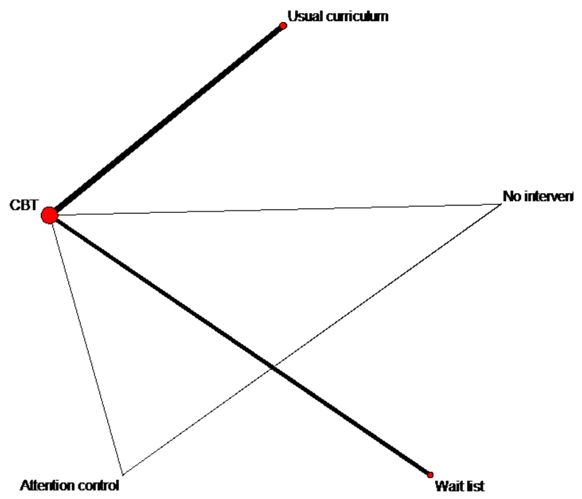

Universal Primary anxiety (k=15)

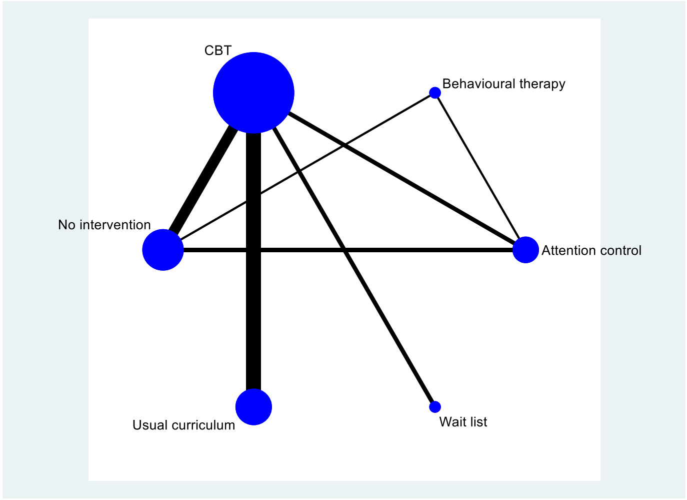

Universal Primary Depression (k = 12)

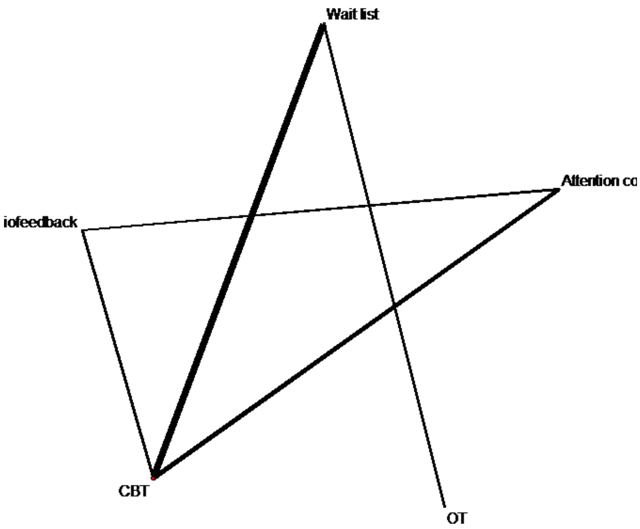

Targeted Primary anxiety (k=11)

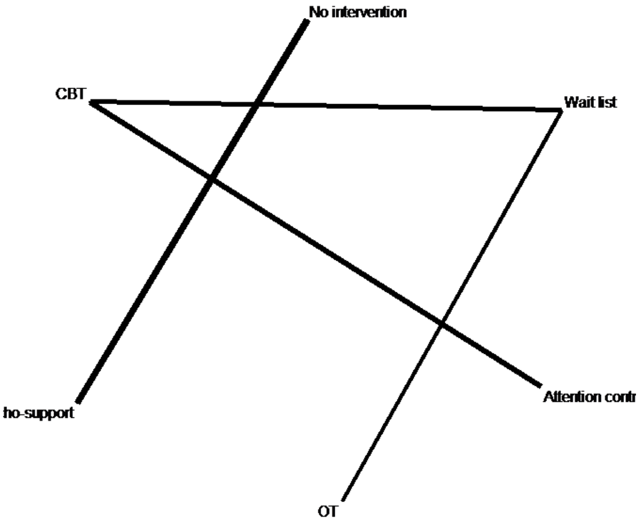

Targeted Primary Depression (k= 5)

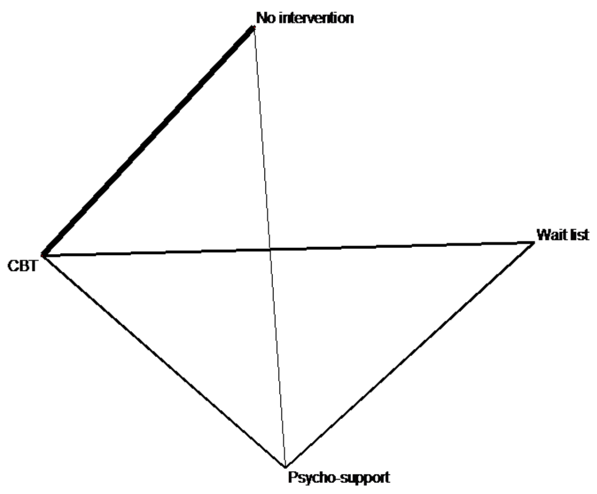

Targeted University Anxiety (k = 4)

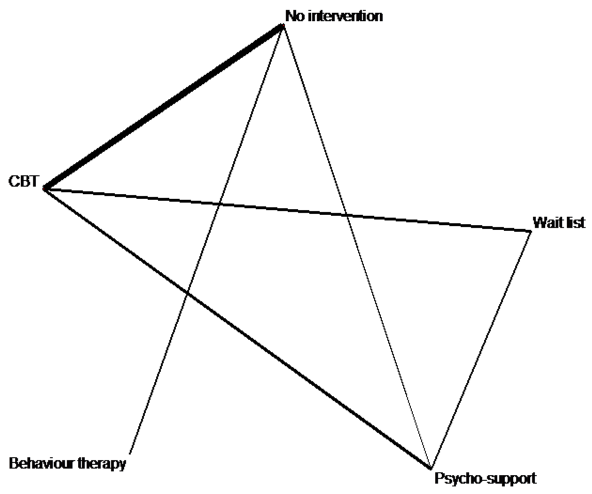

Targeted University Depression (k = 5)

Risk of bias assessment for included studies

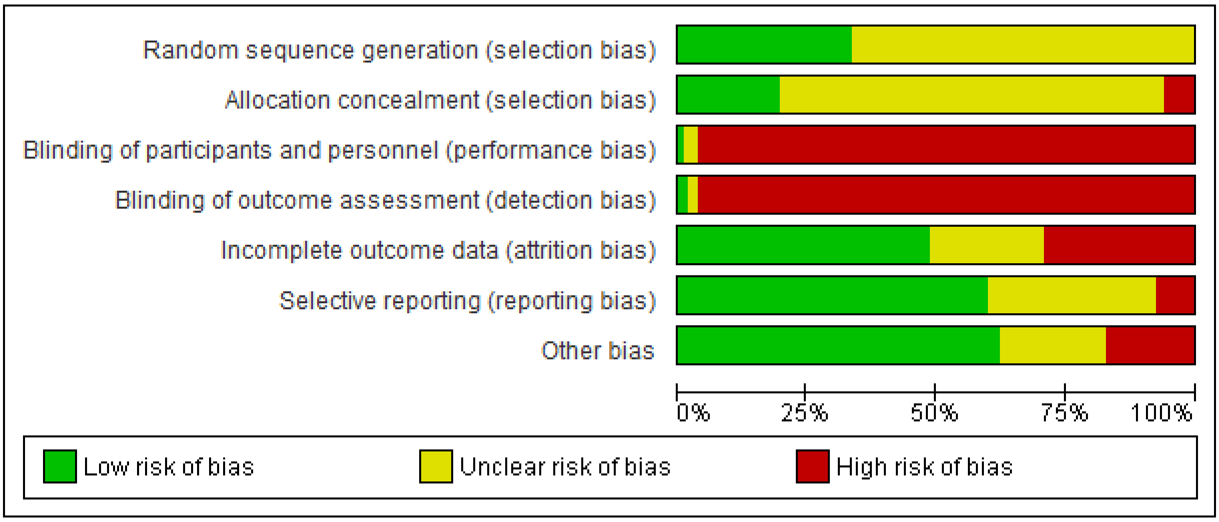

Judgements about each risk of bias item presented as percentages across all included studies. Figure generated using Review Manager (RevMan) [Computer program]. Version 5.3. Copenhagen: The Nordic Cochrane Centre, The Cochrane Collaboration, 2014.

Standard pairwise meta-analyses: results

Pairwise meta-analyses were conducted for all intervention and control comparisons for which direct head-to-head evidence was available. The method of estimation is similar to the NMA, except that the consistency assumption is removed such that intervention effects for separate comparisons are unrelated and separate intervention effects can be estimated. Estimates are reported for the immediate post-intervention main timepoint only and are from a random effects model which assumes the heterogeneity parameter is common across intervention comparisons. This better reflects the assumption made in the NMA and therefore allows a fair comparison of the intervention effect estimates obtained from both approaches. Vague prior distributions were used for all parameters, and convergence is reported in the model fit table above.

Key: UC = usual curriculum, WL = waiting list, NI = no intervention, AC = attention control, CBT = cognitive behavioural therapy, IPT = interpersonal therapy, MR = mindfulness/ relaxation, BT = behavioural therapy, EX = exercise, BM = bias modification, BIO = biofeedback, PS= psycho-support, PE= psycho-education, OT= occupational therapy.

Universal Secondary Depression

Comparator	Intervention	SMD	LCrI	UCrI	# trials
UC	AC	0.3125	-0.04722	0.6838	1
UC	CBT	-0.05349	-0.1692	0.05929	11
UC	Third Wave	-0.02293	-0.1849	0.1404	4
UC	IPT	-0.1006	-0.4696	0.2595	1
UC	PE	-0.1344	-0.4852	0.2159	1
UC	BT	-0.01573	-0.3981	0.3664	1
WL	CBT	-0.02373	-0.176	0.1287	5
WL	CBT+IPT	-0.2094	-0.4611	0.04737	2
NI	AC	-0.0516	-0.3155	0.2061	2
NI	CBT	-0.05342	-0.2025	0.08597	7
AC	CBT	-0.2216	-0.5547	0.1118	2
AC	CBT+IPT	-0.1802	-0.5403	0.1802	1
	sd	0.1477	0.08964	0.2255	-

Universal Secondary Anxiety

Comparator	Intervention	SMD	LCrI	UCrI	# trials
UC	CBT	-0.1462	-0.3281	0.02353	3
UC	Third wave	0.03855	-0.1048	0.1908	3
WL	CBT	-0.07206	-0.1943	0.04095	6
WL	MR	-1.081	-1.766	-0.3903	1
NI	CBT	-0.06886	-0.2521	0.09911	4
AC	CBT	-0.06025	-0.3156	0.1943	1
AC	MR	-0.2891	-0.7401	0.1634	1
	sd	0.09102	0.008562	0.2041	-

Universal Primary Depression

Comparator	Intervention	SMD	LCrI	UCrI	# trials
UC	CBT	-0.1092	-0.3727	0.156	6
WL	CBT	-0.06112	-0.5612	0.488	2
NI	AC	-0.1531	-0.7122	0.3923	2
NI	CBT	-0.2898	-0.6557	0.08007	3
NI	BT	-0.1246	-0.8799	0.6383	1
	sd	0.2832	0.1529	0.5222	

Universal Primary Anxiety

Comparator	Intervention	SMD	LCrI	UCrI	# trials
UC	CBT	-0.07589	-0.2357	0.04189	6
WL	CBT	-0.09352	-0.2537	0.04947	5
NI	AC	-0.3778	-0.9743	0.2158	1
NI	CBT	-0.3051	-0.6494	0.03352	2
AC	CBT	0.1069	-0.2684	0.4741	2
	sd	0.1261	0.07814	0.003294	0.2589

Targeted Secondary Depression

Comparator	Intervention	SMD	LCrI	UCrI	# trials
NI	CBT	-0.1594	-0.4713	0.1457	5
NI	EX	-0.2785	-1.121	0.5658	1
WL	CBT	-0.3992	-0.7088	-0.07846	7
UC	CBT	-0.2521	-0.943	0.4065	2
AC	CBT	0.5943	-0.3344	1.519	1
AC	BM	-0.0875	-0.9147	0.7406	1
PS	CBT	-0.2248	-0.708	0.257	1
PS	Third Wave	-3.76	-4.713	-2.809	1
PS	IPT	-0.6702	-1.202	-0.1622	3
PS	PE	0.2637	-0.5521	1.083	1
CBT	PE	0.2152	-0.5921	1.021	1
sd		0.3712	0.2367	0.5823	

Targeted Secondary Anxiety

Comparator	Intervention	SMD	LCrI	UCrI	# trials
NI	CBT	0.02904	-0.1004	0.159	4
NI	BM	-0.2085	-0.5436	0.1451	1
NI	EX	-0.4707	-0.8563	-0.08129	1
WL	CBT	-0.2728	-0.4588	-0.1017	4
WL	BIO	-0.5856	-1.175	0.01068	1
WL	MR	-0.2439	-0.8275	0.343	1
AC	CBT	-0.02747	-0.4357	0.3801	2
AC	BIO	-0.02919	-0.361	0.3018	1
AC	MR	-0.08843	-0.7604	0.5784	1
AC	BM	-0.01126	-0.3607	0.3387	1
PS	CBT	-1.049	-1.6	-0.5054	1
	sd	0.07376	0.06121	0.002894	0.229

Targeted Primary Depression

Comparator	Intervention	SMD	LCrI	UCrI	# trials
WL	CBT	-0.4787	-2.483	1.472	2
WL	OT	-0.1	-2.87	2.69	1
AC	CBT	0.2457	-1.734	2.21	2
	sd	0.9292	0.07467	3.788	

Targeted Primary Anxiety

Comparator	Intervention	SMD	LCrI	UCrI	# trials
WL	CBT	-0.3517	-0.7941	0.09184	5
WL	OT	0.1111	-0.9331	1.159	1
AC	CBT	-0.03032	-0.552	0.496	4
CBT	BIO	-8.56E-05	-1.035	1.03	1
	sd	0.465	0.212	0.9139	

Results at follow-up timepoints: 6-12 months and 13-24 months.

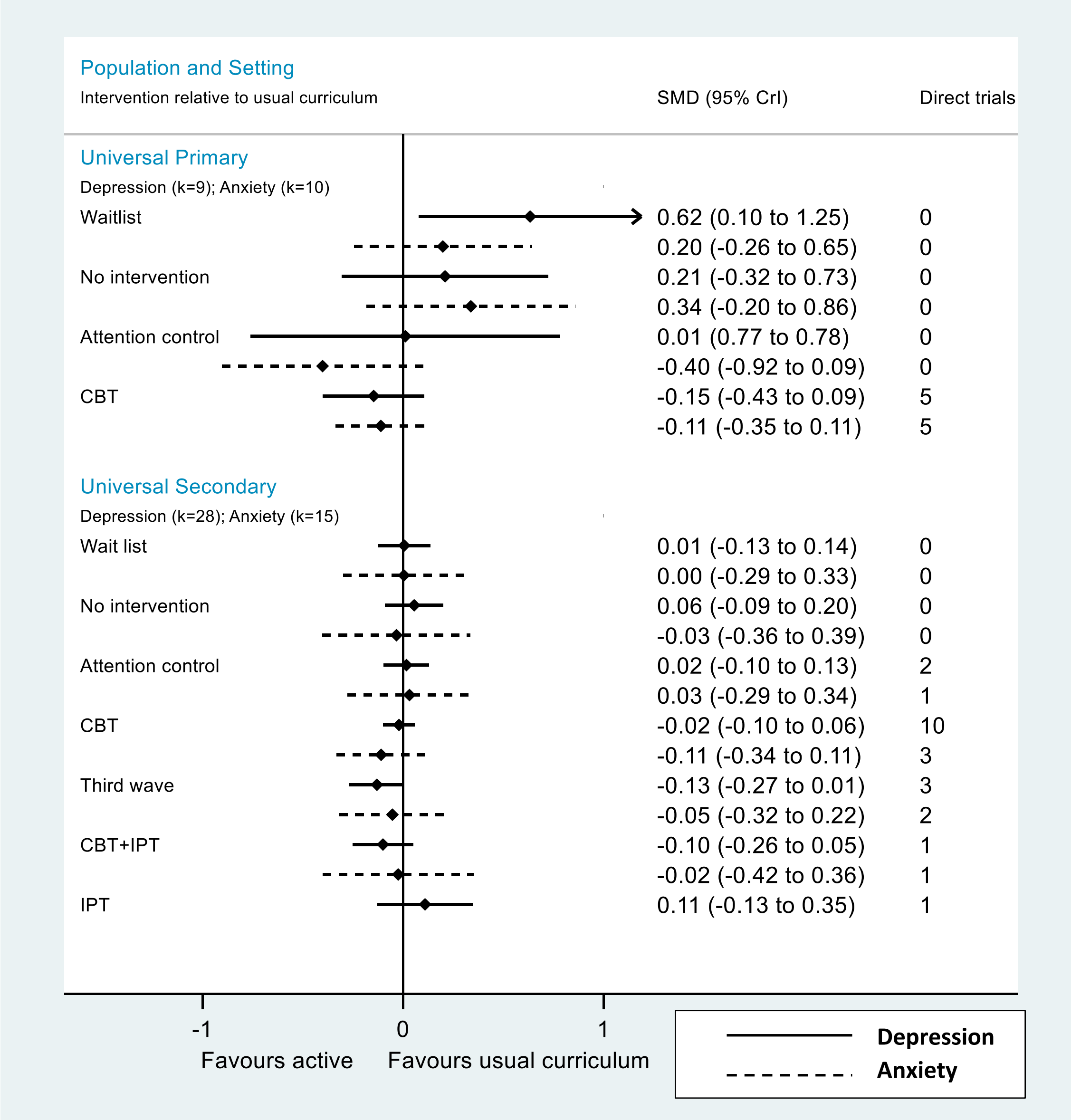

Figure: Self-report depression and anxiety for universal population 6 – 12 months follow up. Active intervention is displayed relative to the reference intervention Usual Curriculum. Effect estimates are based on combination of direct and indirect evidence from a random effects network meta-analysis. Direct trials: number of head-to-head trials in the network making that comparison.

K= number of studies included in NMA. SMD: standardised mean difference; CrI: credible interval; Att. Control: attention control, CBT: cognitive behavioural therapy, IPT: interpersonal therapy, CBT+IPT: cognitive behavioural therapy + interpersonal therapy.

Solid black line: self-report depression. Dotted black line: self-report anxiety

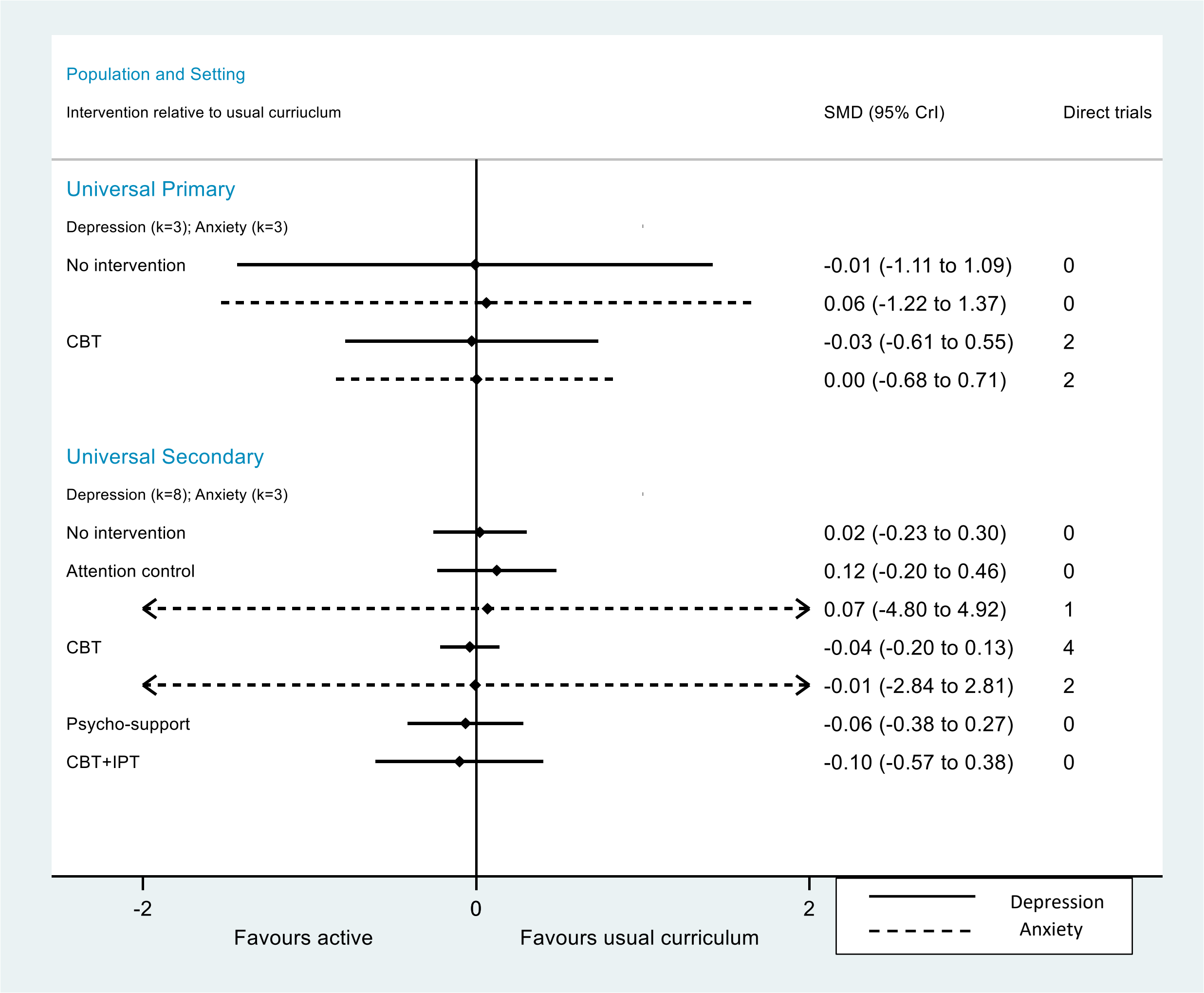

Figure: Self-report depression and anxiety for universal population 13 to 24 months follow up. Active intervention is displayed relative to the reference intervention Usual Curriculum. Effect estimates are based on combination of direct and indirect evidence from a random effects network meta-analysis. Direct trials: number of head-to-head trials in the network making that comparison.

K= number of studies included in NMA. SMD: standardised mean difference; CrI: credible interval; CBT: cognitive behavioural therapy.

Solid black line: self-report depression. Dotted black line: self-report anxiety

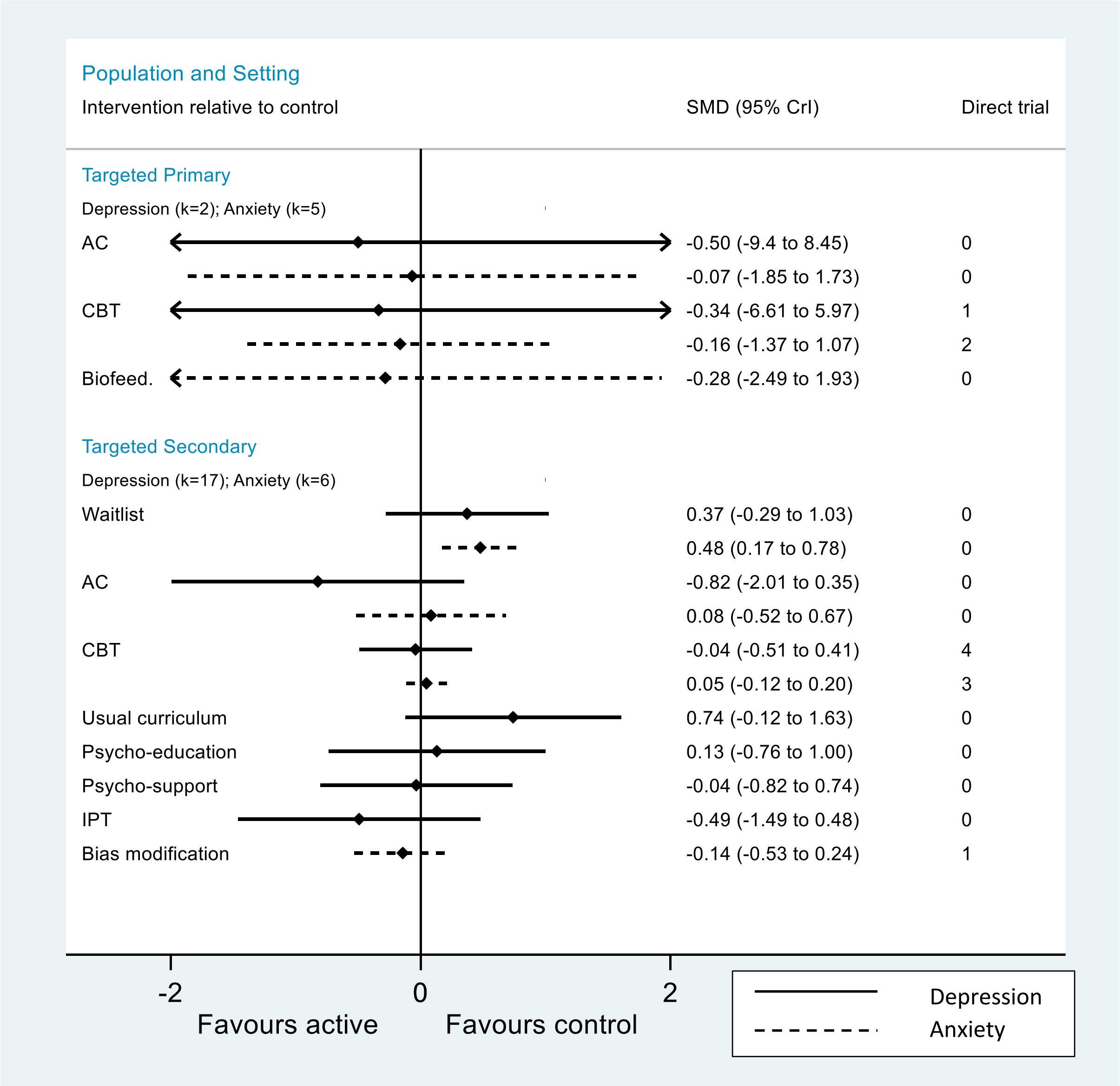

Figure: Self-report depression and anxiety for targeted population at 6-12 months follow-up. For Targeted Primary populations the intervention displayed is relative to waiting list (WL). For Targeted Secondary populations the reference intervention is no intervention (NI). Effect estimates are based on combination of direct and indirect evidence from a random effects network meta-analysis. *. Direct trials: number of head-to-head trials in the network making that comparison.

K= number of studies included in NMA. SMD: standardised mean difference; CrI: credible interval; AC: attention control, CBT: cognitive behavioural therapy, IPT: interpersonal therapy.

Solid black line: self-report depression. Dotted black line: self-report anxiety

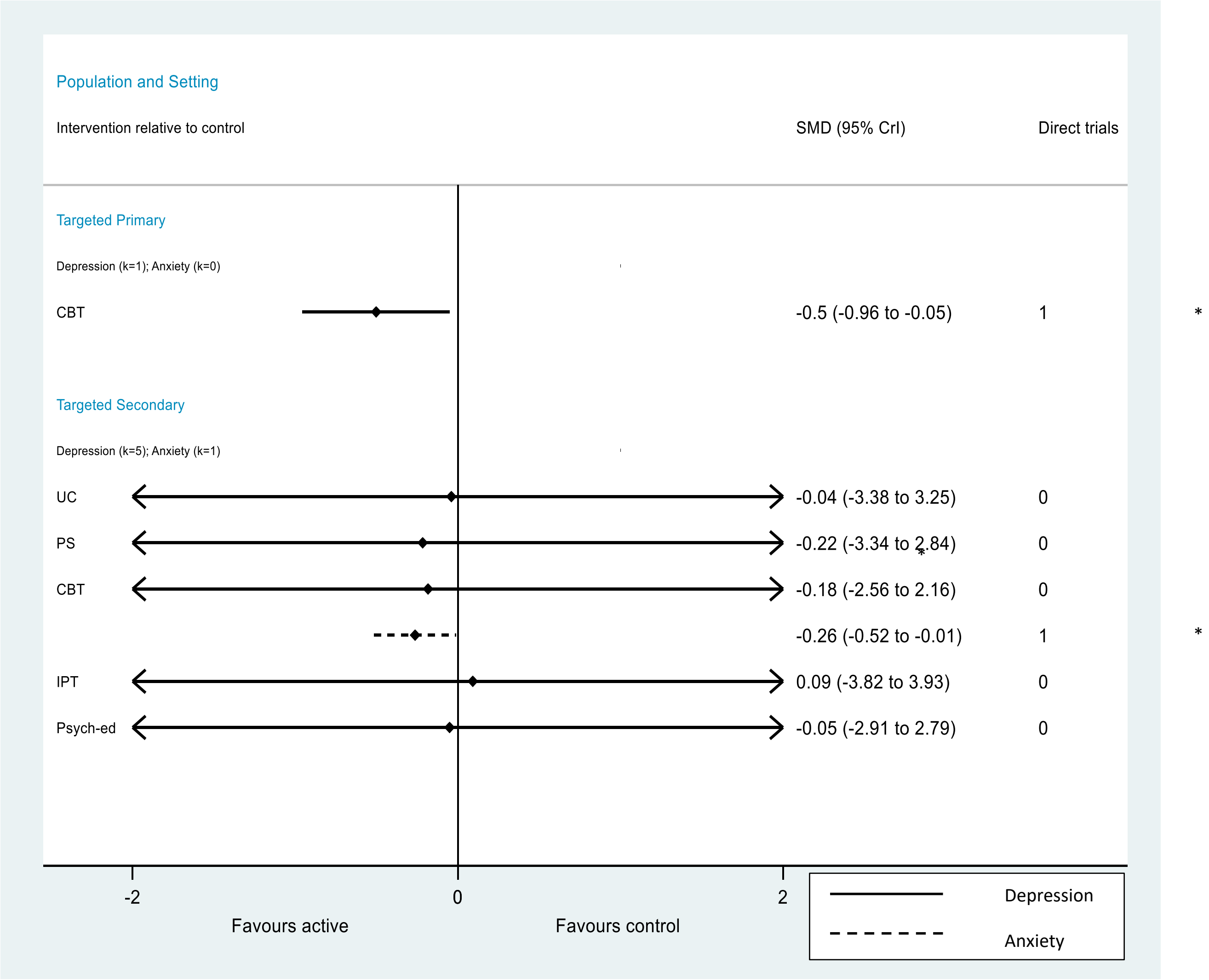

Figure: Self-report depression and anxiety for targeted population at 13-24 months follow-up. For Targeted Primary populations the intervention displayed is relative to waiting list (WL) and is based on a single trial. For Targeted Secondary populations the reference intervention is no intervention (NI). Effect estimates are based on combination of direct and indirect evidence from a random effects network meta-analysis apart from *. Direct trials: number of head-to-head trials in the network making that comparison. *estimate from a single trial

K= number of studies included in NMA. SMD: standardised mean difference; CrI: credible interval; CBT: cognitive behavioural therapy, UC: usual curriculum, PS: psycho-support, IPT: interpersonal therapy, Psych-ed: psycho-education.

Solid black line: self-report depression. Dotted black line: self-report anxiety

University based interventions: forest plot

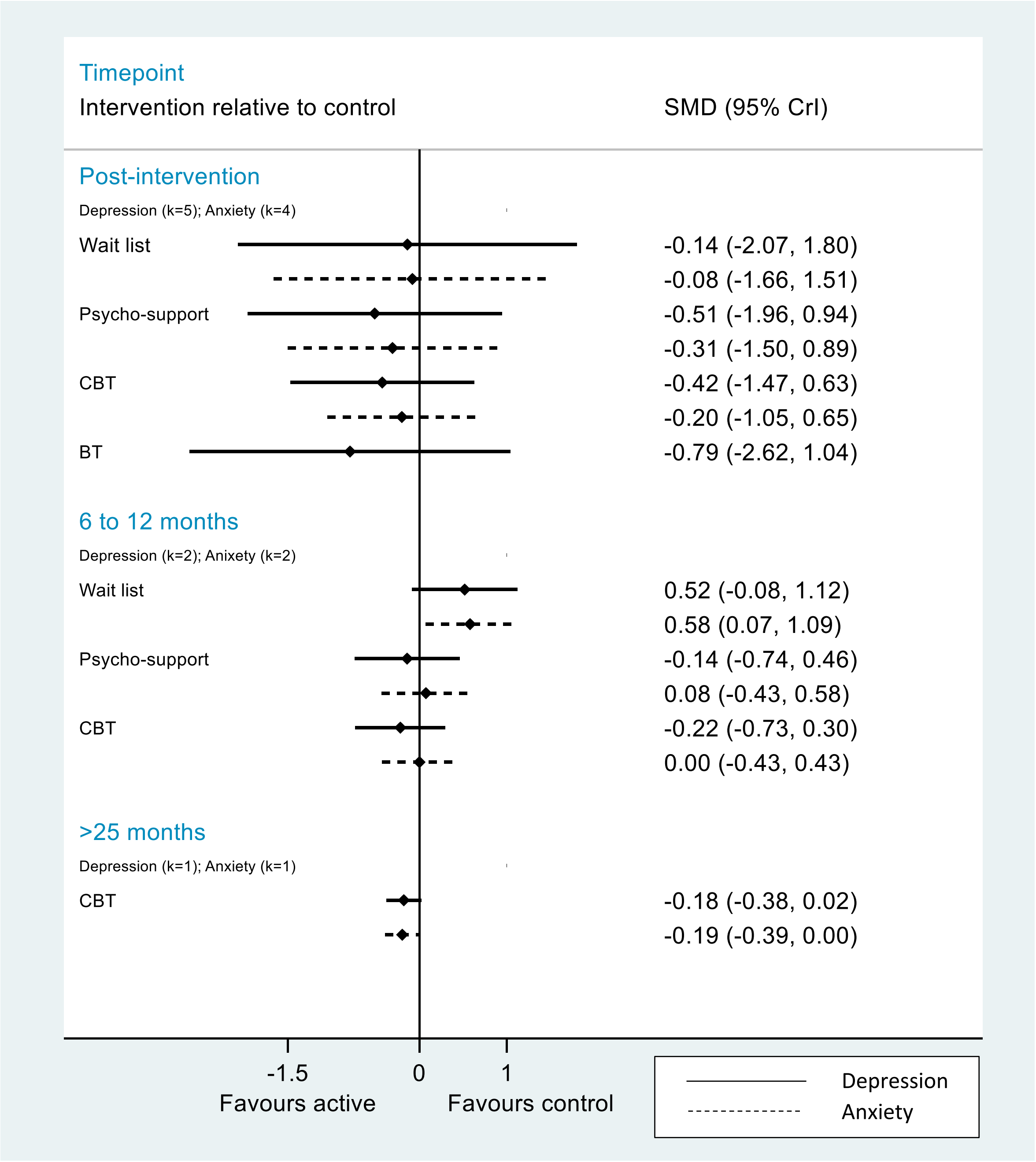

Self-report depression and anxiety for targeted university population (i) immediately post-intervention, (ii) 6-12 months and (iii) >25 post intervention. The intervention displayed is relative to no intervention (NI). Post intervention results from Random effects network meta-analysis. 6-12 months results from fixed effect network meta-analysis

K= number of studies included in NMA. SMD: standardised mean difference; CrI: credible interval; CBT: cognitive behavioural therapy. BT: behavioural therapy

Solid black line: self-report depression. Dotted black line: self-report anxiety

University based interventions: Mean Rank and 95% credible intervals

Intervention	Universal University		Targeted University	
	**Depression**	**Anxiety**	**Depression**	**Anxiety**
Usual curriculum	1.0 (1 to 2)	-	-	-
Waiting list	-	-	3.7 (1 to 5)	2.8 (1 to 4)
No intervention	-	-	4.2 (2 to 5)	3.1 (1 to 4)
Attention control	-	-	-	-
CBT	2.0 (1 to 2)	-	2.7 (1 to 5)	2.2 (1 to 4)
BT	-	-	1.9 (1 to 5)	-
3rd Wave	-	-	-	-
CBT+IPT	-	-	-	-
IPT	-	-	-	-
Mindfulness	-	-	-	-
Psychoeducation	-	-	-	-
Psycho-support	-	-	2.5 (1 to 5)	1.9 (1 to 4)
Occupational therapy	-	-	-	-
Biofeedback	-	-	-	-
Bias modification	-	-	-	-
Exercise	-	-	-	-

Mean Rank and 95% credible intervals by population and setting, for primary endpoint of immediately post-intervention.

Results for wellbeing outcome immediately post-intervention

Across all populations and settings, 14 studies reported a measure of psychological wellbeing, quality of life or life satisfaction. Five studies reported wellbeing using the Warwick-Edinburgh scale, eight studies report a measure of life satisfaction and two studies report a quality of life measure. One study reports a measure of social functioning and one reports the Ryff Psychological wellbeing scale.

Planned NMAs by population and setting groupings were not possible and data are reported by outcome measure and study in Appendix H. Two studies suggest that wellbeing was considerably improved in intervention participants; Calear (2016a) (n=1767) evaluated computerised-CBT and Livheim (2015) (n=58) examined acceptance and commitment-based therapy, a 'third-wave' intervention. Both were in secondary educational settings. However, for each of the remaining 12 studies summary intervention effects are compatible with both an increase and decrease in wellbeing/ life satisfaction.

Warwick-Edinburgh mental wellbeing scale

Study	Population	Setting	Comparison	Results: mean difference [95% CIs]
Calear 2016a	Universal	Secondary	CBT vs CBT vs waitlist	Post-intervention: -2.07 [-2.14, -2.00]<br>Post-intervention: -1.09 [-1.14, -1.04]<br>6m follow-up: -3.94 [-4.00, -3.88]<br>6m follow-up: -2.87 [-2.92, -2.82]<br>12m follow-up: -1.41 [-1.48, -1.34]<br>12m follow-up: -1.79 [-1.85, -1.73]
Calear 2016b	Universal	Secondary	CBT vs waitlist	Post-intervention: 1.85 [-0.35, 4.05]<br>3m follow-up: 4.35 [1.46, 7.24]
Johnson 2016	Universal	Secondary	3rd wave vs usual curriculum	Post-intervention: 0.01 [-0.12, 0.14]<br>3m follow-up: -0.05 [-0.19, 0.09]
Johnson 2017	Universal	Secondary	3rd wave vs 3rd wave vs usual curriculum	Post-intervention: 0.02 [-0.10, 0.14]<br>Post-intervention: -0.06 [-0.18, 0.06]<br>6m follow-up: -0.01 [-0.15, 0.13]<br>6m follow-up: -0.04 [-0.16, 0.08]<br>12m follow-up: 0.11 [-0.03, 0.25]<br>12m follow-up: -0.01 [-0.14, 0.12]
Burckhardt 2015	Universal	Secondary	Mindfulness/relaxation vs attention ctrl	Post-intervention data not reported

Life satisfaction scales

Hodas 2015	Universal	Secondary	CBT vs waitlist	Post-intervention: 1.74 [-1.28, 4.76]<br>6m follow-up: -0.51 [-3.01, 1.99]
Khalsa 2012	Universal	Secondary	Mindfulness/ relaxation vs usual curriculum	Post-intervention: 0.03 [-0.29, 0.35]
Rose 2014	Universal	Secondary	CBT+IPT vs CBT+IPT vs waitlist	Post-intervention: 0.13 [-0.12, 0.38]<br>Post-intervention -0.13 [-0.37, 0.11]<br>5m follow-up: -0.05 [-0.28, 0.18]<br>5m follow-up: -0.08 [-0.29, 0.13]<br>14m follow-up: 0.12 [-0.14, 0.38]<br>14m follow-up: -0.08 [-0.32, 0.16]
Burckhardt 2015	Universal	Secondary	Mindfulness/relaxation vs attention ctrl	Post-intervention data not reported
Stallard 2014	Universal	Primary	CBT vs CBT vs usual curriculum	12m follow-up: -0.58 [-1.26, 0.10]<br>12m follow-up: 0.03 [-0.66, 0.72]
Livheim 2015	Targeted	Secondary	3rd wave vs psycho-support	Post-intervention: 6.77 [5.64, 7.90]
Tokolahi 2018	Targeted	Primary	Occupational therapy vs waitlist	Post-intervention: -0.45 [-3.39, 2.49]
Seligman 2007	Targeted	Tertiary	CBT vs no intervention	Post-intervention: 0.10 [-1.06, 1.26]<br>1m follow-up: 0.40 [-0.78, 1.58]

Quality of Life

Guhct 2017	Universal	Secondary	3rd wave vs usual curriculum	Post-intervention: 0.09 [-0.26, 0.44]<br>12m follow-up: 0.27 [-0.14, 0.68]
Takagaki 2016	Targeted	University	Behaviour therapy vs no intervention	Post-intervention: 0.05 [0.02, 0.08]

Social Functioning

Spence 2003	Universal	Secondary	CBT vs usual curriculum	Post-intervention: 0.22 [-0.70, 1.14]

Ryff's psychological wellbeing scale

Tomba 2010	Universal	Secondary	CBT vs CBT	Multiple sub-scales reported: autonomy, environmental mastery, personal growth, positive relations, purpose in life and self-acceptance.

Suicidal ideation, behaviour and self-harm outcomes

34 studies provided information on suicidal ideation, suicide attempt or self-harm behaviours. Of these, the majority (n=21) report either excluding participants reporting suicidal thoughts or behaviours and/or removed questions asking about suicide and self-harm from the baseline questionnaires. Some studies report the removal of questions was requested by participating schools or education authorities. 11 studies report that suicidal thoughts and behaviours were measured at baseline and that participants were referred to further services where necessary (Stice 2007, Stice 2008). However, 9 do not provide details on whether these students continued in the study nor provide follow-up measures. 7 studies reported participants experiencing suicidal thoughts or self-harm at post-intervention. 3 studies were conducted in a universal secondary setting (Perry 2017, Stallard 2012, Britton 2014) and are connected via attention control. However, model fit was suggestive of inconsistency and so combined results are not reported. 1 study reported self-harm behaviours (Stallard). 1 study in a universal primary setting reported suicide risk. 2 studies in an indicated secondary setting (Poppelaars 2016, Cova 2011) and 1 in an indicated tertiary setting (Takagaki 2016). There is no evidence to suggest that educational-setting based interventions to prevent common mental disorders impact suicidal ideation or self-harm.

Network meta-analysis of suicidal ideation and thoughts of self-harm: universal secondary setting

Model fit consistency: DIC = 45.01 , posterior mean residual deviance = 6.10, datapoints = 7. SD = 4.96 [0.26 to 9.75]
Model fit inconsistency (independent effects): DIC = 45.1, posterior mean residual deviance = 6.23, datapoints = 7, SD = 1.99 [0.09 to 3.89]

Results reported by individual study, due to suspected inconsistency

Study	Population	Setting	Comparison	Results
Perry 2017	Universal	Secondary	Attention control vs CBT	OR 0.83 (95% CrI 0.28 to 2.40
Stallard 2012a	Universal	Secondary	Attention control vs usual curriculum<br>Attention control vs CBT+IPT	OR 0.87 (95% CrI 0.72 to 1.04)<br>OR 0.83 (95% CrI 0.70 to 1.00)
Britton 2012	Universal	Secondary	Attention control vs Mindfulness/relaxation	Not estimable

Study	Population	Setting	Comparison	Results
Takagaki 2016	Indicated	University	No intervention vs Behavioural activation	OR 0.39 (95% CrI 0.05 to 2.28)

Study	Population	Setting	Comparison	Results
Poppelaars 2016	Indicated	Secondary	Waitlist vs CBT	OR 2.20 (95% CrI 0.29 to 65.56)
Cova 2011	Indicated	Secondary	No intervention vs CBT	Results not presented due to missing SD

Study	Population	Setting	Comparison	Results
Roberts 2018	Universal	Primary	Usual curriculum vs CBT vs CBT	"For suicidal ideation, there was no significant group time interaction [F(2,198) = 2.84, p = 0.061]. There were, however, significant main effects for group [F(2,198) = 3.41,

				p = 0.035] and time [F(1,198) = 6.14, p = 0.014]. The main effect for group indicated that, at both post-test 2 and follow-up, the control group had significantly higher incidence rates than the training/coaching group (p = 0.017)."

Studies reporting participants with suicidal behaviours or thoughts were excluded

Study	Population	Setting	Quote/ details
McCarty 2011	Indicated	Secondary	Suicidal ideation was an exclusion criterion
Tokolahi 2018	Selective	Primary	Children were excluded if they reported para/ suicidal thoughts or behaviours
Young 2016	Indicated	Secondary	Youth were also excluded if they endorsed significant suicidal ideation or non-suicidal self-injury (n=11)
Kindt 2014	Universal	Secondary	"The item on suicide was removed in the current study to optimize collaboration with school officials and parents" CDI, item 9
McCarty 2013	Indicated	Secondary	Current suicidal ideation was an exclusion criterion
Peden 2000	Indicated	University	Participants included If "not suicidal as indicated by Beck Depression inventory (BDI) rating on question 9, (I don't have thoughts of killing myself)"
Rohde 2014	Indicated	Secondary	Acute suicidal ideation used as exclusion criteria. All participants completed assessments that allowed to monitor depression and suicidal ideation and contact parents and provide treatment referrals as necessary
Young 2010	Indicated	Secondary	10 Adolescents were excluded because of a current depression diagnosis, suicidal ideation or self-harm behaviours
Livheim 2015	Indicated	Secondary	Suicidality as an exclusion criterion: Counsellors/welfare coordinators were instructed not to nominate participants if they were experiencing severe symptoms, suicidality, or complete withdrawal from school
Cowell 2009	Selective	Primary	Suicidal ideation was an exclusion criterion: "Mothers and children with depression scores at the cut point for depression or suicidal ideation were referred for mental health assessment"
Wijnhoven 2014	Indicated	Secondary	Girls who already received mental health care (n=9) or had a clinical CDI-score and suicidal ideation (CDI score>19 and score 2 on item 9, n=3) were excluded from further participation. In the latter case, they were referred to a social worker from mental health care who provided further help when necessary.
Seligman 1999	Selective	University	Not meeting criteria for any of the following Axis I disorders: current major depression, past major depression with psychotic features (past major depression was not an exclusion criterion), past or current mania, current dysthymia, current cyclothymia, past or current psychosis, current suicide risk, past or current alcohol or substance dependence, current alcohol or substance abuse, current panic disorder, current panic disorder with agoraphobia, current agoraphobia without panic disorder, current obsessive–compulsive disorder, current somatization disorder, current hypochondriasis, current undifferentiated somatoform disorder, current anorexia or bulimia.

Studies reporting schools requested questions excluded

Hodas 2015	Universal	Secondary	The question which asks about suicide was removed from the CDI
Johnstone 2014	Universal	Primary	In this study, the suicide item was omitted from the CDI scale as school principals voiced concern about the use of this item with children as young as eight. National Health and Medical Research Council Risk Assessment measures suicidal ideation and behaviour. Children who endorsed any of the critical suicide items on the DICA-IV were administered this additional assessment which asks children to (i) rate their risk of harming themselves, (ii) state the strength of their desire to kill themselves, (iii) disclose recent events that may have led to these feelings, (iv) disclose any methods, plans, and previous attempts, (v) state what could stop them, (vi) list who they would like to support them, and (vii) state what they have to look forward to in the future. Authors do not report how this changed over the intervention. I.e. whether intervention reduced these feelings
Pophillat 2016	Universal	Primary	Item 9 of CDI (relating to suicidal ideation) was removed in accordance with the WA Department of Education's standards,
Soffer 2003	Universal	Primary	Page 82 This study originally proposed to use the CDI to assess depressive symptomatology. However, the administrator from the New York City Board of Education's Proposal Review Committee did not approve of the CDI due to its explicit assessment of suicidality
Tak 2016	Universal	Secondary	Due to ethical considerations, the CDI item concerning suicidal thoughts and ideation was omitted from the questionnaire after the baseline assessment
Chaplin 2006	Universal	Secondary	Item 9, which assesses suicidality, was removed from the questionnaire (Children's depression inventory CDI) at the request of school administrators
Gillham 2012	Indicated	Secondary	"At the request of school administrators, we removed items that ask about suicidal thoughts and self-injurious behaviours (questionnaires: Children's Depression Inventory (CDI item 9) and Reynolds Adolescent Depression Scale, 2nd Edition (RADS-2)) item 14
Horowitz 2007	Universal	Secondary	"The item about suicidal ideation was removed because of concerns of the participating schools" Questionnaire: Children's Depression Inventory CDI item 9
Pössel 2013	Universal	Secondary	Severity of depressive symptoms was assessed with the Children's Depression Inventory (CDI; Kovacs, 1981), a 26-item measure of cognitive, affective, and behavioural symptoms of depression (the suicide item was removed at the request of the school, as is common in school-based research)

Descriptions of socio-economic status, gender and ethnicity as extracted from authors reports: universal interventions

Trial	Setting	Gender	Ethnicity	SES
Ahlen 2018	Primary	Mixed	NC	Median of household income US$6000–7000/month
Anticich 2013	Primary	Mixed	Catholic Education preschools and primary schools in the greater metropolitan area of Brisbane	"White and working to middle class"
Araya 2013	Secondary	Mixed	NC	"Socially deprived" - School Social Deprivation Index, mean (SD) 0.85 (0.1) 0.85 (0.1)
Attwood 2012	Primary	Boys	NC	NC
Aune 2009	Secondary	Mixed	Norwegian - "less than 3% non-Caucasian"	NC
Baker 1984	Secondary	Mixed	White/ Caucasian	NC
Barrett 2001	Primary	Mixed	"predominantly from Anglo-Saxon families with English as their primary language"	"Children came from both dual-parent (75.35%) and single-parent (11.55%) families"… "Schools selected for approach were representative of varying levels of socio-economic advantage and religious affiliation"
Barrett 2005	Secondary	Mixed	"The majority of children attending these schools were white, Anglo-Saxon, Catholic or Protestant Christian,"	" schools represented diverse levels of socio-economic status"… "working to middle class"
Barry 2017	Secondary	Boys	20 participants described themselves as 'white or white Irish', two indicated they were of 'any other white background', and one ticked the box for 'other, including mixed ethnic background'.	NR
Bonhauser 2005	Secondary	Mixed	Chile	"a low socioeconomic area in Santiago."… "The percentage of the population living below the poverty level ... is 15%."
Bouchard 2013	Primary	Mixed	NC	"schools that were equally distributed among low-, average-, high-, and very high-income neighbourhoods."
Britton 2014	Secondary	Mixed	NC	"an independent Quaker school"
Burckhardt 2015	Secondary	Mixed	NC	"These four schools were among the highest in terms of socioeconomic status compared to other schools in Australia."
Burckhardt 2016	Secondary	Mixed	NC	"an independent Episcopalian high school"… "76% of the students were in the top quartile of socio-economic advantage"

Calear 2009	Secondary	Mixed	94% of participants indicated that English was their first language. Of those students who reported a language other than English as their first language, 15% spoke Chinese, 13% spoke Hindi, and 8% and 6% spoke Arabic and Indonesian respectively	"a mix of public, private, coeducational,single-sex, metropolitan, and rural schools from six Australian states"
Calear 2016	Secondary	Mixed	88% reported English as their first language. Of those students who reported a language other than English as their first language 38% spoke Chinese,10% spoke Vietnamese ,and 7% and 3% spoke Indian and Arabic respectively.	NC
Calear 2016b	Secondary	Mixed	97% of participants reported English as their first language	NC
Cardemil 2002	Primary	Mixed	school 1: 77.2% were Latino children predominately of Puerto Rican descent (n = 754), 11.7% were African American (n = 114), 7.8% were Caucasian (n = 76), and 2.8% were Asian (n = 27); School 2: 98.9% were African American (n = 819), 0.6% were Asian (n = 5), 0.2% were Latino (n =2), and 0.2% were Caucasian (n = 2);	95.3% of the students from School 1 come from low-income families… 89.8% of the students from School 2 come from low-income families
Chaplin 2006	Secondary	Girls	Mostly White (88.7%), with 4.1% African American, 1.5% Latino, 1% Asian American, and 4.6% more than one race or ethnicity	Median reported family income was $100,000 or more per year in 1997,
Clarke 1993a	Secondary	Mixed	"90% of enrolled students identified as White"	"Participating schools were located in predominantly middle-class neighborhoods"
Clarke 1993b	Secondary	Mixed	"90% of enrolled students identified as White"	"Participating schools were located in predominantly middle-class neighborhoods"
Collins 2014	Primary	Mixed	98% British white	"The low FSME percentage in the present study reflects that all schools were located in relatively affluent suburbs."
Dadds 2008	Primary	Mixed	The majority of participants (86.8%) were white Anglo-Saxon,	the vast majority were working to middle class
Eather 2016	Secondary	Mixed	NC	NC
Essau 2012	Primary	Mixed	Most of the youth indicated Catholic as their religious affiliation (63%), followed by Protestant (10.9%). Almost all the sample was of German origin (95%), with the remainder coming from other ethnic backgrounds, mostly from Southern and Eastern Europe	Parental education ranged from elementary school through university and college degrees, with most parents (72%) reporting a high school educational level
Gallegos 2008	Primary	Mixed	Northern Mexico	about 70% of the population that lives in the metropolitan area of Monterrey is considered to be of a medium SES, ranked as number 6.
Gillham 1994	Primary	Mixed	NC	NC

Gillham 2006	Secondary	Mixed	The majority of students were Caucasian. Two students were of African American descent, one of Asian descent, and one student identified her race as “other."	the suburban Philadelphia area. 15 (47%) reported household incomes above $100,000, 11 (34%) between $60,000 and $99,999, and the remaining 6 (19%) below $60,000.
Gillham 2007	Secondary	Mixed	The majority of students were Caucasian. Less than 10% were of African American descent, less than 2% of latino/a, less than 3% Asian descent,	Income differed by school. In school 1 39% reported income level above 100,000 and 72% above 60,000. In schools 2 and 3 84% and 66% reported family inocme < 60,000
Guhct 2017	Secondary	Mixed	Dutch-speaking region of Belgium	NC
Haden 2014	Primary	Mixed	"Most participants were... White"	estimated family income falling into either the $10,000 to $75,000 range or the over $125,000 range.
Hiebert 1989b	Secondary	Mixed	large suburban centre in Western Canada	NC
Hodas 2015	Secondary	Girls	School 1: Approximately 72% of the students enrolled in this school are Caucasian. School 2: Approximately 45% of the students enrolled in this school are African American and 43% are Caucasian.	School 1: A majority of students from this school come from affluent families who are able to afford the nearly $27,000 annual tuition. School 2: more economically diverse than the families from the other school
Horowitz 2007	Secondary	Mixed	The sample was 79% Caucasian, 13% African American, 2% Latino, 1% Asian American, 1% Native American, 3% mixed heritage	The schools served communities characterized as predominantly working (e.g., sales clerks, factory workers) to middle class (e.g., farmers, mechanics).
Johnson 2016	Secondary	Mixed	NC	16.2% of students were in the low SES band, 39% were in the medium category, and 44.8% were in the high SES category
Johnson 2017	Secondary	Mixed	NC	Participating schools represented a broad range of socioeconomic (SES) demographics
Johnstone 2014	Primary	Mixed	NC	these schools are within the largest (top50%) and poorest (bottom30%) in the WA Department of Education and Training School Database.
Khalsa 2012	Secondary	Mixed	The school had a 90% white student body demographic	a 17% low-income population
Kindt 2014	Secondary	Mixed	Roughly 50% of participants were classed as "ethnic minority"	Schools recruited if at least 30% of students came from Low income areas
Lock 2003	Secondary	Mixed	NC	socioeconomically diverse schools in the metropolitatian area of Brisbane,
Lowry-Webster 2001	Secondary	Mixed	NC	Catholic schools in the Brisbane metropolitan area
Mendelson 2010	Primary	Mixed	Eighty-one students (83.5%) selfidentified as African American, four (4.1%) self-identified as Latino, four (4.1%) as White, and seven (7.2%) as “mixed race”	Baltimore City public elementary schools

Merry 2004	Secondary	Mixed	The schools were selected on the basis of their ethnic mix, almost purely Maori and Pakeha,	One of these schools, school A, was from a lower socioeconomic urban area; the other, school B, was from a middle-class rural district
Miller 2010	Primary	Mixed	"... a population that spoke English in 88% of homes…"	"...an unemployment rate of 5.5%...."
Miller 2011a	Primary	Mixed	Mixed. 36% Canadian aborginal - First Nations, North American Indian, Metis, and Inuit)	NC
Miller 2011c	Primary	Mixed	18% spoke a language other than English in the home	NC
Pahl 2010	Primary	Mixed	NC	Approximately 19% of the sample had an annual income under $40,000, 38.7% between $40,001 and $80,000, and 28% between $80,001 and $100,000 and over.
Pattison 2001	Primary	Mixed	NC	"a rural town south of Adelaide"
Perry 2017	Secondary	Mixed	Roughly 43% spoke a language other than English at home	Selective and partially selective government secondary schools in metropolitan Sydney
Pophillat 2016	Primary	Mixed	NC	"… a very low socioeconomic status area in Perth…"
Possel 2004	Secondary	Mixed	NC	NC
Possel 2008	Secondary	Mixed	NC	a wide range of social classes is likely to be represented because students from schools in economically different regions of the area are represented
Possel 2013	Secondary	Mixed	The sample was 72.8% Caucasian, 14.7% African American, 5.4% Latino, 1.4% Asian/Pacific Islander, 0.8% Native American, 4.4% mixed geritage, and 0.6% "other	Predominantly working to middle class. According to county data, 29% of the students were eligible for free or reduced-price lunches.
Potek 2012	Secondary	Mixed	Total sample was 77.4% White; 16.1% Black; 3.2% Latino and 3.2% East Asian	NC
Quayle 2001	Primary	Girls	NC	A private girls school in a high socioeconomic suburb of Perth
Raes 2014	Secondary	Mixed	NC	NC
Reynolds 2011	University	Mixed	"Racial distribution was as follows: 57.7% White (not of Hispanic origin), 12.7% African American, 11.3% Hispanic, 8.5% Asian or Pacific Islander, 5.6% Asian Indian, and 4.2% self-identified as "other"."	NC
Rivet-Duval 2011	Secondary	Mixed	"The nationality of the majority of participants was Mauritian (97.5%) and the sample was representative of the ethnic (primarily Creole, Hindu and Muslim) and religious (primarily Christian, Hindu and Muslim) backgrounds of the Mauritian population."	Household income: 10% <Rs 5,000; 19% Btw Rs 5,000 and Rs 15,000; 33% Btw Rs 15,000 and Rs 25,000; 38% > Rs 25,000

Roberts 2003	Secondary	Mixed	Ethnic origin: 74% Australian; 3% Australian Aboriginal; 5% United Kingdom and Ireland; 3% European; 0.5% Other non-English speaking; 15% Not stated	Mother's and Father's education respectively: 9% and 10% less than grade 10; 52% and 39% grades 10-12; 18% and 17% grade 12; 20% and 16% vocational college; 5% and 6% university; 5% and 10% not stated
Roberts 2010	Secondary	Mixed	Ethnic origin: 44% Australian; 4% Other English speaking; 7% Other non-English speaking; 44% Not stated	"The schools were randomly sampled from the lowest decile of socio-economic status (SES) based on the Census Index of Relative Socio-economic Status." Annual family income (Aus$): 14% less than $20,000; 30% $20,000-$50,000; 13% greater than $50,000; 43% not stated
Roberts 2018	Primary	Mixed	Of the 2076 students who reported their ethnic origin, 80.7% (n = 1675) identified as Australian, 1.7% (n = 36) as Australian Aboriginal, 9.2% (n = 191) from other English speaking countries, 5% (n = 104) as Asian, 1.9% (n = 39) as European, and 1.5% (n = 31) from other non-English speaking countries.	NC
Rodgers 2015	Secondary	Mixed	By School 1, 2 and 3 respectively: 68%, 92% and 88% White Irish; 18%, 0% and 0% Irish travelling community; 14%, 8% and 12% Foreign nationals	"secondary schools in a socially disadvantaged catchment area in a major city in Ireland"
Rooney 2006	Primary	Mixed	NC	"Four state primary schools were randomly selected from low socioeconomic areas, as identified by the Australian Council for Educational Research"
Rose 2014	Secondary	Mixed	Mixed	Mixed
Ruttledge 2016	Primary	Mixed	NC	Mixed
Sawyer 2010	Secondary	Mixed	NC	"81% of participants had at least one parent in full-time employment, while 70% of participants' parents lived together, consistent with national Australian population estimates"
Shatte 1997	Secondary	Mixed	NC	NC
Sheffield 2006a	Secondary	Mixed	"Students came from a broad range of social and cultural backgrounds, consistent with the Australian population in general"	NC
Soffer 2003	Primary	Mixed	60% (n = 43) of the participants were Caucasian, 14% (n = 10) were African American, 8% (n = 6) Hispanic American, 8% (n = 6) Asian American, 4% (n = 3) were mixed ethnicity, and 6% (n = 4) were of other ethnicity	"The average SES rating from the Hollingshead Index for the sample was 35.37 (SD = 11.67). The majority of the participants' parents reported that they were employed within the skilled craftsmen, clerical, and sales worker domain, which is considered to fall in the middle-income range (scores from 30-39)."

Spence 2003	Secondary	Mixed	In the intervention condition, "90.1% of the students were born in Australia, with the remainder coming from a wide variety of ethnic backgrounds typical of the Australian population"	"The average SES rating for the intervention school students was 4.55 (SD 2.66), typical of the SES distribution of Australia in general. This value is indicative of lower middle SES, on average (e.g., trades occupations are coded as 4, clerical occupations as 5). The intervention condition included 523 students from six state schools, and 228 students from two private schools."
Stallard 2012a	Secondary	Mixed	"The eight schools were representative of schools in the United Kingdom for ethnicity"	"The eight schools were representative of schools in the United Kingdom for deprivation (eligibility for free school meals), pupil absence rates, and academic ability (examination results and proportion of children with identified special educational needs)"
Stallard 2014	Primary	Mixed	94% White british; 6% non-white	Family affluence: 2% Low; 29% Medium; 69% high. Eligibility for free school meals was lower (12·4% vs 18·2%) in the cohort than the national average.
Tak 2016	Secondary	Mixed	Intervention group 79% Dutch, 21% other. The percentage of adolescents included from ethnic minorities was somewhat lower compared to the overall population, 16.9 % compared to 20.3 % respectively.	NC
Tomba 2010	Secondary	Mixed	NC	NC
Velásquez 2015	Primary/Secondary	Mixed	NC	"a public school located in a socioeconomic disadvantaged area in the city of Bogotá, Colombia"
Wong 2014	Secondary	Mixed	NC	NC

Descriptions of socio-economic status, gender and ethnicity as extracted from authors reports: targeted interventions

NC= not clear. NR = not reported

Study	Setting	Gender	Ethnicity	Socio-Economic Status
Arnarson 2009	Secondary	Mixed	NC	NC
Balle 2010	Secondary	Mixed	NC	NC
Berry 2009	Secondary	Boys	"The predominant family ethnicity was Anglo-Saxon (74%, n ¼ 34), followed by Middle Eastern (17%, n=8) and Asian (9%, n=4)"	"The majority of parents (54%, n = 25) had not completed tertiary education and were from lower to middle class backgrounds (76%, n = 35) based on their annual household income."
Clarke 1995	Secondary	Mixed	"92.5% non-Hispanic white"	"Median parent education was 1 to 2 years of college"
Congelton 1995	Secondary	Mixed	"97% Caucasian"	"25% on free or reduced lunch"
Cooley-Strickland 2011	Primary	Mixed	92% African American, 8% biracial	"Both schools were located in economically disadvantaged urban communities (average of 90% of the student bodies received free or reduced lunch)"
Cova 2011	Secondary	Girls	NC	NC
Cowell 2009	Primary	Mixed	"Elementary schools in Chicago with enrollments of 30% or more Latinos were selected and randomly assigned to intervention or control conditions."	Over 80% of mothers reported incomes of less than $26,000 per year.
Cui 2016	University	Mixed	NC	NC
Dobson 2010	Secondary	Mixed	NC	NC
Ellis 2011	University	Mixed	NC	NC (although university students)
Fitzgerald 2016	Secondary	Mixed	93% White; 2% Black; 2% Asian; 0% Irish Traveller; 1% Other and 2% unknown	School disadvantages status (DEIS): Non-DEIS 82%; DEIS 18%
Fung 2016	Secondary	Mixed	"10 students (52.6 %) self-identified as Latino and 9 (47.4 %) as Asian-American. Fifteen (78.9 %) students were born in the USA while the remaining four foreign-born students have lived in the USA between 4 and 13 years"	"elementary schools in an urban public school district in the greater Los Angele area that serves a high proportion of ethnic minority and low-income immigrant families."
Gaete 2016	Secondary	Mixed	NC	"virtually all of them coming from low socio-economic families."
Gillham 2012	Secondary	Mixed	<1% Native American; 4% Asian; <1% Pacific Islander/Native Hawaiian; 12% African American; 77% European American; 3% Latino/a; 4% Other	Mothers' and Fathers' Education (respectively): 2% and 5% Some high school; 19% and 26% High school graduate; 19% and 19% Some college; 38% and 28% College Graduate; 22% and 22% advanced degree
Hiebert 1989a	Secondary	Mixed	NC	NC

Higgins 2006	University	Mixed	"Approximately 95% of the sample was Caucasian, while the remaining participants described themselves as Asian (2.6%), African American (1.3%), and Other (1.3%)."	NC
Hunt 2009	Secondary	Mixed	"Catholic secondary schools in the metropolitan area of Sydney"	NC
Jaycox 1994	Primary	Mixed	Treatment group: 80% Caucasian; 17% African American; 3% Other	Total family income (intervention group): 16% Less than $20,000; 44% $20,001-$40,000; 26% $40,001-$60,000; 7% $60,001-$80,000; 7% more than $80,000
Jordans 2010	Secondary	Mixed	Caste/Ethnicity (Nepal): 45% Brahmin/Chhetri/Thakuri; 25% Tharu; 16% Terai caste; 8% Dalit; 7% Other Jannajati	Setting is "Nepal, the poorest country in South Asia"
Kiselica 1994	Secondary	Mixed	White	"Although no formal assessment of SES occurred, the participants were all residents of an area described to consist primarily of middle-class anf lower middle-class families."
Liddle 2010	Primary/Secondary	Mixed	NC (Scottish setting)	NC
Livheim 2014	Secondary	Girls	NC	NC
Manassis 2010	Primary	Mixed	Ethnicity as reported by families was: 56.8% Caucasian, 12.8% Asian, 8.1% East Indian, 6.8% Hispanic, 5.4% Phillipino, 3.3% Black, and 6.8% Mixed/Other	"Our sample was ethnically and economically diverse"
McCarty 2011	Secondary	Mixed	Race in intervention group: 67% White; 3% African American; 6% Asian; 6% Native American; 19% Other. Ethnicity 3% Hispanic; 97% Non-Hispanic	Parental education in intervention group: 36% HS Diploma/GED/Some College; 50% Associates/Bachelor's degree; 14% Masters/Professional/Doctoral Degree
McCarty 2013	Secondary	Mixed	Race in intervention group: 63% White; 5% African American; 15% Asian; 8% Native American; 2% Native Hawaiian/Pacific Islander; 8% Other. Ethnicity 7% Hispanic; 94% Non-Hispanic	Parental education in intervention group: 52% HS Diploma/GED/Some College/AA; 23% Bachelor's degree; 26% Masters/Professional/Doctoral Degree. Annual Household Income (intervention group): 38% <50,000; 26% 50,000 to 100,000; 36% >100,000
McLaughlin 2011	Primary/Secondary	Mixed	The racial/ethnic composition included 94% Caucasian, 2% Hispanic, 2% African American, 2% Asian and/or Pacific Islander, and less than 1% American Indian.	"The school district had approximately 11% of students participating in free and reduced lunch services"
McLoone 2012	Primary	Mixed	NC	School based intervention group, mother's and Father's job level (respectively): 25% and 2% unemployed; 32% and 15% Trade/clerical; 44% and 84% Professional
Mifsud 2005	Primary	Mixed	Ethnicity of Treatment group: 78% Australian; 17% Other country; 5% Aboriginal	"To obtain a sample of children disadvantaged by socioeconomic factors, the schools involved were selected by their Priority Schools Funding Program categorization. This program identifies those schools in which the community has the highest concentrations of families disadvantaged by socioeconomic factors."

Miller 2011b	Primary	Mixed	"Notably, 48% of the sample reported speaking a language other than English in the home (predominately Chinese, 18%, with over twelve other languages represented)."	NC
Noël 2013	Secondary	Girls	"The racial-ethnic composition of the treatment group was 80% African American, 15% non-Hispanic white, and 5% Hispanic"	NC
Owen 1982	Secondary	Boys	NR	NR
Peden 2000	University	Girls	NC	NC
Peng 2015	Secondary	Mixed	NC	NC
Poppelaars 2016	Secondary	Girls	"The vast majority of participants were born in The Netherlands (94.7%)."	NC
Puskar 2003	Secondary	Mixed	NC	NC
Rice 2008	Secondary	Mixed	NC	NC
Rohde 2014	Secondary	Mixed	"The sample was composed of 6% Hispanics, 2% Asian Americans, 1% African Americans, 72% Caucasians, 1% Native American, and 18% who specified other or mixed heritage."	"Educational attainment of parents, a proxy for socioeconomic status, was 39% high school graduate or less; 26% some college; 22% college graduate; 13% graduate degree"
Scholten 2016	Secondary	Mixed	"Nearly all adolescents were born in the Netherlands (97.8%) and the remaining adolescents were born in Turkey, Indonesia, or South Africa (2.2%)."	"the majority of the sample came from high-streamed education tracks."
Schoneveld 2016	Primary	Mixed	"The majority of the children were of Dutch descent (89.7%)."	NC
Schoneveld 2018	Primary	Mixed	"The majority of the children were born in the Netherlands (91.4%)."	NC
Seligman 1999	University	Mixed	NC	NC
Seligman 2007	University	Mixed	NC	NC
Sheffield 2006b	Secondary	Mixed	"Students came from a broad range of social and cultural backgrounds, consistent with the Australian population in general"	NC
Simpson 2008	Primary	Mixed	"56% of the sample was Caucasian, 38% were of Asian/South Asian decent, and 6% were of mixed decent"	"Of the 45 participants (68%) who provided information regarding income, 35.2% reported an annual income over $80 000, 24.6% of the families had an annual income of less than $35 000, and 19.3% reported an income of $35 000 per year. Of the 52 mothers (79%) who provided information regarding their highest level of education, 68.5% completed some post-secondary education, 14% did not complete high school and 8.8% completed high school. Of the 46 fathers who provided regarding their highest level of education, 40% completed some post-

				secondary information education, 19.3% did not complete high school, and 7% completed high school"
Siu 2007	Primary	Mixed	NC	NC
Sportel 2013	Secondary	Mixed	NC	NC
Stallard 2012b	Secondary	Mixed	"The eight schools were representative of schools in the United Kingdom for ethnicity"	"The eight schools were representative of schools in the United Kingdom for deprivation (eligibility for free school meals), pupil absence rates, and academic ability (examination results and proportion of children with identified special educational needs)"
Stice 2006	Secondary/University	Mixed	"The sample was composed of 17% Asians, 6% Blacks, 55% Caucasians, 15% Hispanics, and 7% who specified other or mixed racial heritage"	"Educational attainment of parents, a proxy for socioeconomic status, in our sample (20% high school graduate or less; 20% some college; 34% college graduate; 26% graduate degree) was similar to census data for the county (34% high school graduate or less; 25% some college; 26% college graduate; 15% graduate degree)."
Stice 2008	Secondary	Mixed	"The sample was composed of 2% Asians, 9% African Americans, 46% Caucasians, 33% Hispanics, and 10% who specified other or mixed heritage"	"Educational attainment of parents, a proxy for socioeconomic status, was 26% high school graduate or less; 17% some college; 35% college graduate; 18% graduate degree."
Stoppelbein 2003	Secondary	Mixed	88% Caucasian; 10% African American; 2% Asian American	Socioeconomic status: 18% Lower; 22% Lower Middle; 51% Middle; 9% Upper Middle
Takagaki 2016	University	Mixed	NC	NC
Tokolahi 2018	Primary	Mixed	Ethnicities of total sample: 35% NZE; 16% Maori; 18% Pacific; 10% Asian; 20% Other	NC
Topper 2017	Secondary	Mixed	NC	NC
van Starrenburg 2017	Primary	Mixed	"The majority of children (92.9%) and mothers (90.8%) were born in The Netherlands."	"Most mothers (55%) finished a vocational education, and about 25% had college or higher education. Approximately 40% of the families had a low to average income."
Wijnhoven 2014	Secondary	Girls	"Most of the adolescents were of Dutch origin (98 %)."	NC
Woods 2011	Secondary	Mixed	"About 45% of the students in the present study identified as Maori and 55% as Pacific"	"Schools were selected from across the socioeconomic range using Ministry of Education decile rankings (Ministry of Education, 1997)."
Young 2006	Secondary	Mixed	"The majority of the adolescents were Hispanic (92.7%)"	"half reported a gross household income of $25,000 or less."
Young 2010	Secondary	Mixed	"A majority of the adolescents (73.7%) identified themselves as Hispanic" (39% were African American)	NC

Young 2016	Secondary	Mixed	"A third of the adolescents were racial minorities (19.9 % African American, 4.3 % Asian, and 8.1 % who specified other or mixed race); 38.2 % were Hispanic and 38.2 % were White non-minority, non-Hispanic"	"Regarding gross income, there was a wide range represented as follows: 17.3 % of families earned less than $25,000, 38.4 % earned between $25,000 and $90,000, and 44.3 % earned more than $90,000"
Yu 2002	Primary/Secondary	Mixed	NC	Education of father and mother respectively, in treatment group: 5% and 4% Elementary school graduate; 9% and 6% Junior school graduate; 29% and 34% Senior school graduate; 28% and 45% college graduate; 28% and 11% more than collage. Family monthly income (treatment group): 17% lower than 1,000 yuan; 25% 1,001-2,000 yuan; 24% 2,001-3,000 yuan; 17% 3,001-4,000 yuan; 17% more than 4,001 yuan.

Results from subgroup analysis for socio-economic status

11 studies reported they were conducted in lower SES settings (as defined by the study author), of which 3 were conducted in middle-income and 8 in high-income countries (HIC). For the primary timepoint of post-intervention, data were only available for the studies conducted in HIC. There was no evidence of a difference by SES for depression or anxiety in primary settings. However, for self-report anxiety the effect of CBT vs usual curriculum in higher SES settings was SMD -0.15 (95% CrI -0.37 to 0.02) and in lower SES settings was SMD 0.05 (95% CrI -0.08 to 0.78). In universal secondary school settings, results suggest that interventions delivered in lower SES settings were less effective than those in higher/mixed settings for reducing self-report anxiety symptoms (Higher SES: SMD -0.29 (95% CrI -0.50 to -0.07) and Low SES: SMD 0.09 (95% CrI -0.11 to 0.29)). This was not observed for depression. Due to insufficient data, sub-group analyses for gender or ethnicity were not conducted

Population/setting	Outcome	Comparison	Low SES		High SES	
Universal Secondary	Depression	CBT v UC	0.04	-0.06 to 0.15*	-0.07	-0.20 to 0.06
	Anxiety	CBT v UC	0.09	-0.11 to 0.29*	-0.29	-0.50 to -0.07
Universal primary	Depression	CBT v UC	-0.23	-0.60 to 1.13	-0.05	-0.55 to 0.45
	Anxiety	CBT v UC	0.05	-0.08 to 0.78*	-0.15	-0.37 to 0.02

*Results from fixed effect analysis. SES: socio-economic status. SMD and 95% Credible intervals reported by subgroup (low vs high SES)

Only CBT vs usual curriculum (UC) could be compared in each subgroup

Results from Risk of bias sensitivity analyses

Population/setting	Control	Intervention	Trials	Low RoB	All
Universal Secondary Depression	UC	CBT	5	0.02 (-0.77 to 0.80)	-0.04 (-0.16 to 0.07)
	UC	Third wave		-0.35 (-1.15 to 0.45)	-0.03 (-0.21 to 0.14)
Universal Secondary Anxiety	WL	CBT	3	-0.06 (-0.15 to 0.01)	-
Universal Primary Depression	UC	CBT	1	-0.10 (-0.29 to 0.09)*	-0.13 (-0.44 to 0.17)
Universal Primary Anxiety	UC	CBT	1	-0.01 (-0.18 to 0.17)*	-0.07 (-0.23 to 0.05)
Targeted Secondary Depression	NI	CBT	4	0.07 (-1.33 to 1.49)	-0.22 (-0.58 to 0.13)
Targeted Secondary Anxiety	NI	CBT	3	0.07 (-0.25 to 0.41)	0.03 (-0.11 to 0.16)
	NI	Bias Modification		-0.20 (-0.69 to 0.30)	-0.17 (-0.45 to 0.11)

Results listed by population, setting and outcome. Results from low risk of bias studies compared to the main analyses reported in the accompanying paper.

Results compared for immediate post-intervention timepoint. Comparisons listed are those remaining once studies at high/ unclear risk of bias for random sequence generation and allocation concealment had been removed from network.

*From fixed effect analysis

Sensitivity analysis to ICC values

Intra-cluster correlation coefficients were available from the following references: 3,16, 23, 49, 66, 70, 72, 94, 96, 111, 129, 132. However, few studies reported ICCs by outcome and instead reported ranges for all variables. ICCs reported ranged from 0.00 to 0.16. In larger studies, with clearly defined outcome specific ICCs, these ranged from 0.001 to 0.05.

Where studies were cluster randomised and extracted results were raw (unadjusted) means and standard deviations, we followed the Cochrane Handbook's guidance on calculating an approximate sample size (section 16.3). We followed this even where the authors also reported conducting an appropriate statistical analysis to account for clustering, since the reported raw means and standard deviations are not model based results.

**Intervention**	**Universal Secondary Depression (19 of 34 trials)**			
	**ICC= 0.01**		**ICC =0.06**	
Wait list	-0.02	95% CrI (-0.22 to 0.18)	-0.01	95% CrI (-0.2 to 0.18)
No intervention	0.03	95% CrI (-0.15 to 0.21)	0.04	95% CrI (-0.13 to 0.21)
Attention control	0.09	95% CrI (-0.10 to 0.27)	0.09	95% CrI (-0.08 to 0.26)
CBT	-0.04	95% CrI (-0.15 to 0.07)	-0.04	95% CrI (-0.15 to 0.07)
Third wave	-0.04	95% CrI (-0.21 to 0.14)	-0.03	95% CrI (-0.19 to 0.13)
IPT+CBT	-0.23	95% CrI (-0.57 to 0.10)	-0.25	95% CrI (-0.58 to 0.07)
IPT	-0.03	95% CrI (-0.36 to 0.30)	-0.03	95% CrI (-0.33 to 0.27)
Psychoeducation	-0.13	95% CrI (-0.48 to 0.21)	-0.13	95% CrI (-0.47 to 0.2)
Behaviour therapy	-0.02	95% CrI (-0.39 to 0.36)	-0.02	95% CrI (-0.4 to 0.36)
**SD**			0.13 (0.07, 0.20)	

Intervention	Universal Primary Depression (7 of 12 trials)			
	ICC= 0.01		ICC =0.06	
Wait list	-0.08	95% CrI (-0.77 to 0.55)	-0.09	95% CrI (-0.77 to 0.53)
No Intervention	0.14	95% CrI (-0.40 to 0.67)	0.12	95% CrI (-0.40 to 0.64)
Attention control	-0.06	95% CrI (-0.79 to 0.63)	-0.07	95% CrI (-0.78 to 0.61)
CBT	-0.13	95% CrI (-0.44 to 0.18)	-0.13	95% CrI (-0.44 to 0.17)
BT	-0.09	95% CrI (-1.04 to 0.82)	-0.1	95% CrI (-1.03 to 0.79)
**SD**	0.33 (0.19, 0.60)		0.31 (0.15, 0.58)	

Intervention	Targeted Secondary Depression (5 of 24 trials)			
	ICC= 0.01		ICC =0.06	
Wait list	0.2	95% CrI (-0.30 to 0.70)	0.2	95% CrI (-0.32 to 0.7)
Usual curriculum	0.05	95% CrI (-0.72 to 0.83)	0.03	95% CrI (-0.73 to 0.82)
Attention control	-0.81	95% CrI (-1.82 to 0.20)	-0.82	95% CrI (-1.84 to 0.18)
Psycho-support	0.03	95% CrI (-0.63 to 0.67)	0.02	95% CrI (-0.65 to 0.67)
CBT	-0.21	95% CrI (-0.58 to 0.14)	-0.22	95% CrI (-0.60 to 0.14)
Third wave	-3.74	95% CrI (-4.90 to -2.58)	-3.74	95% CrI (-4.92 to -2.58)
IPT	-0.65	95% CrI (-1.51 to 0.18)	-0.66	95% CrI (-1.52 to 0.18)
Bias modification	-0.9	95% CrI (-2.22 to 0.41)	-0.91	95% CrI (-2.24 to 0.40)
Exercise	-0.28	95% CrI (-1.14 to 0.56)	-0.28	95% CrI (-1.16 to 0.60)
Psychoeducation	0.12	95% CrI (-0.50 to 0.73)	0.11	95% CrI (-0.52 to 0.72)
**SD**	0.38 (0.25, 0.58)		0.38 (0.25, 0.59)	

Intervention	Targeted Primary Depression (1 of 5 trials)			
	ICC= 0.01		ICC =0.06	
Attention control	-0.71	95% CrI (-3.51 to 2.15)	-0.73	95% CrI (-3.56 to 2.07)
CBT	-0.47	95% CrI (-2.46 to 1.54)	-0.48	95% CrI (-2.48 to 1.50)
Occupational therapy	-0.1	95% CrI (-2.92 to 2.76)	-0.1	95% CrI (-2.90 to 2.72)
**SD**	0.60 (0.08, 3.82)		0.59 (0.06, 3.81)	

Intervention	Universal Secondary Anxiety (12 of 21 trials)			
	ICC= 0.01		ICC =0.06	
**Wait list**	-0.12	95% CrI (-0.38 to 0.14)	-0.1	95% CrI (-0.35 to 0.16)
**No intervention**	-0.14	95% CrI (-0.44 to 0.14)	-0.12	95% CrI (-0.42 to 0.18)
**Attention control**	-0.26	95% CrI (-0.67 to 0.12)	-0.2	95% CrI (-0.59 to 0.12)
**CBT**	-0.22	95% CrI (-0.44 to -0.01)	-0.2	95% CrI (-0.42 to 0.01)
**Third wave**	0.03	95% CrI (-0.17 to 0.24)	0.04	95% CrI (-0.14 to 0.22)
**Mindfulness/Relaxation**	-0.75	95% CrI (-1.30 to -0.23)	-0.7	95% CrI (-1.21 to -0.22)
SD	0.15 (0.08, 0.27)		0.11 (0.01, 0.24)	

Intervention	Universal Primary Anxiety (11 of 15 trials)			
	ICC= 0.01		ICC =0.06	
**Wait list**	0.01	95% CrI (-0.21 to 0.21)	0.02	95% CrI (-0.2 to 0.23)
**No Intervention**	0.22	95% CrI (-0.15 to 0.58)	0.23	95% CrI (-0.16 to 0.62)
**Attention control**	-0.19	95% CrI (-0.52 to 0.12)	-0.15	95% CrI (-0.51 to 0.22)
**CBT**	-0.08	95% CrI (-0.24 to 0.04)	-0.07	95% CrI (-0.23 to 0.06)
SD	0.11 (0.01, 0.27)		0.08 (0.00, 0.25)	

Intervention	Targeted Secondary Anxiety (5 of 15 trials)			
	ICC= 0.01		ICC =0.06	
**No Intervention**	0.31	95% CrI (0.11 to 0.53)	0.29	95% CrI (0.08 to 0.53)
**Attention control**	-0.09	95% CrI (-0.39 to 0.21)	-0.09	95% CrI (-0.39 to 0.22)
**Psycho-support**	1.09	95% CrI (0.53 to 1.63)	1.08	95% CrI (0.51 to 1.64)
**CBT**	0.03	95% CrI (-0.10 to 0.16)	0.03	95% CrI (-0.11 to 0.17)
**Biofeedback**	-0.17	95% CrI (-0.55 to 0.21)	-0.17	95% CrI (-0.55 to 0.21)
**Mindfulness/ relaxation**	0.04	95% CrI (-0.41 to 0.49)	0.03	95% CrI (-0.41 to 0.48)
**Bias modification**	-0.17	95% CrI (-0.44 to 0.10)	-0.17	95% CrI (-0.45 to 0.13)
**Exercise**	-0.47	95% CrI (-0.83 to -0.12)	-0.47	95% CrI (-0.89 to -0.04)
SD	0.06 (0.00, 0.21)		0.06 (0.00, 0.21)	

Intervention	Targeted Primary Anxiety (2 of 11 trials)			
	ICC= 0.01		ICC =0.06	
**Attention control**	-0.36	95% CrI (-1.05 to 0.06)	-0.35	95% CrI (-1.06 to 0.33)
**CBT**	-0.39	95% CrI (-0.85 to 1.14)	-0.38	95% CrI (-0.85 to 0.07)
**Occupational therapy**	0.11	95% CrI (-0.93 to 0.72)	0.11	95% CrI (-0.93 to 1.15)
**Biofeedback**	-0.39	95% CrI (-1.50 to 0.72)	-0.38	95% CrI (-1.51 to 0.73)
SD	0.42 (0.21, 0.90)		0.42 (0.21, 0.90)	

Comparison-adjusted funnel plots to explore small study effects

In standard meta-analysis a funnel plot is a scatter plot of the study specific treatment effect estimates and their standard errors (SE). By convention the vertical axis (SE) is reported in reverse, so that studies with smaller SEs would be seen at the top of the plot. Comparison-adjusted funnel plots follow this same convention but are modified to allow for multiple treatments and multiple comparisons from NMA. In the following graphs we plot active treatments versus inactive control only. The x axis reports the difference of each study's estimate (yiXY) from the direct summary effect for each comparison (yiXY −µXY), and the y axis reports the SE of yiXY. The red line represents the null hypothesis that the comparison-specific pooled effect estimates do not differ from the study-specific effect sizes. In the absence of small study effects all points should be symmetric around the null.

Following Chaimani A *et al* (2013) Graphical Tools for Network Meta-Analysis in STATA. PLOS ONE 8(10): e76654. https://doi.org/10.1371/journal.pone.0076654 the comparisons included in these funnel plots are for a control vs active intervention. Specific interventions are listed after each graph.

Targeted Primary Anxiety

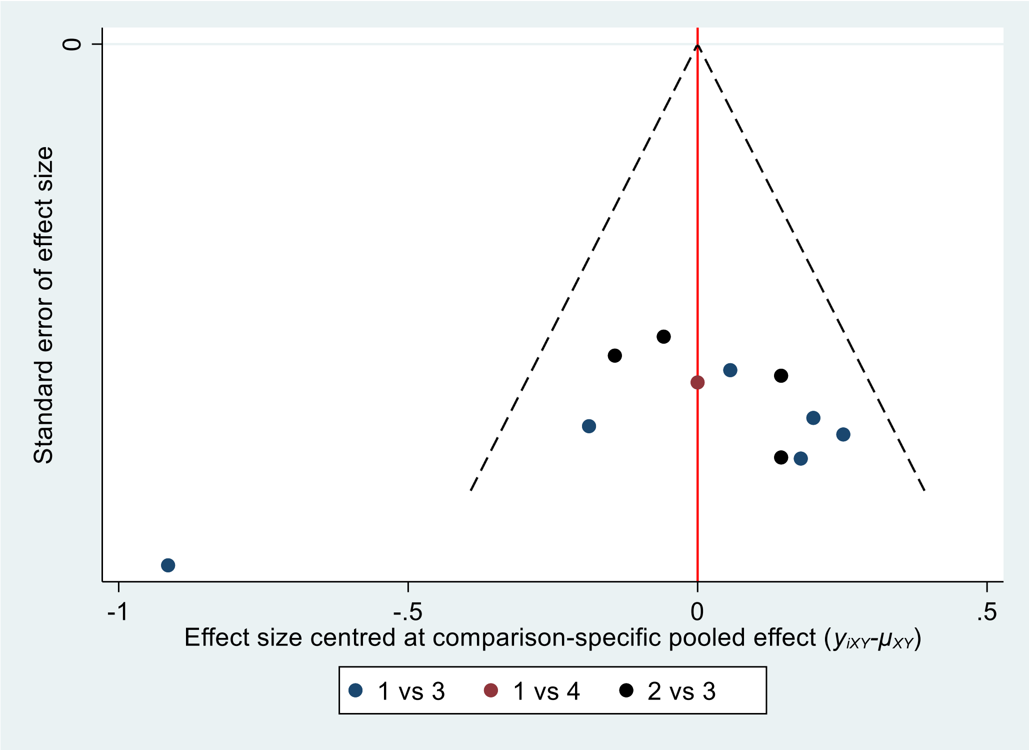

1= Waiting list 2= Attention control, 3= CBT

Targeted Primary Depression

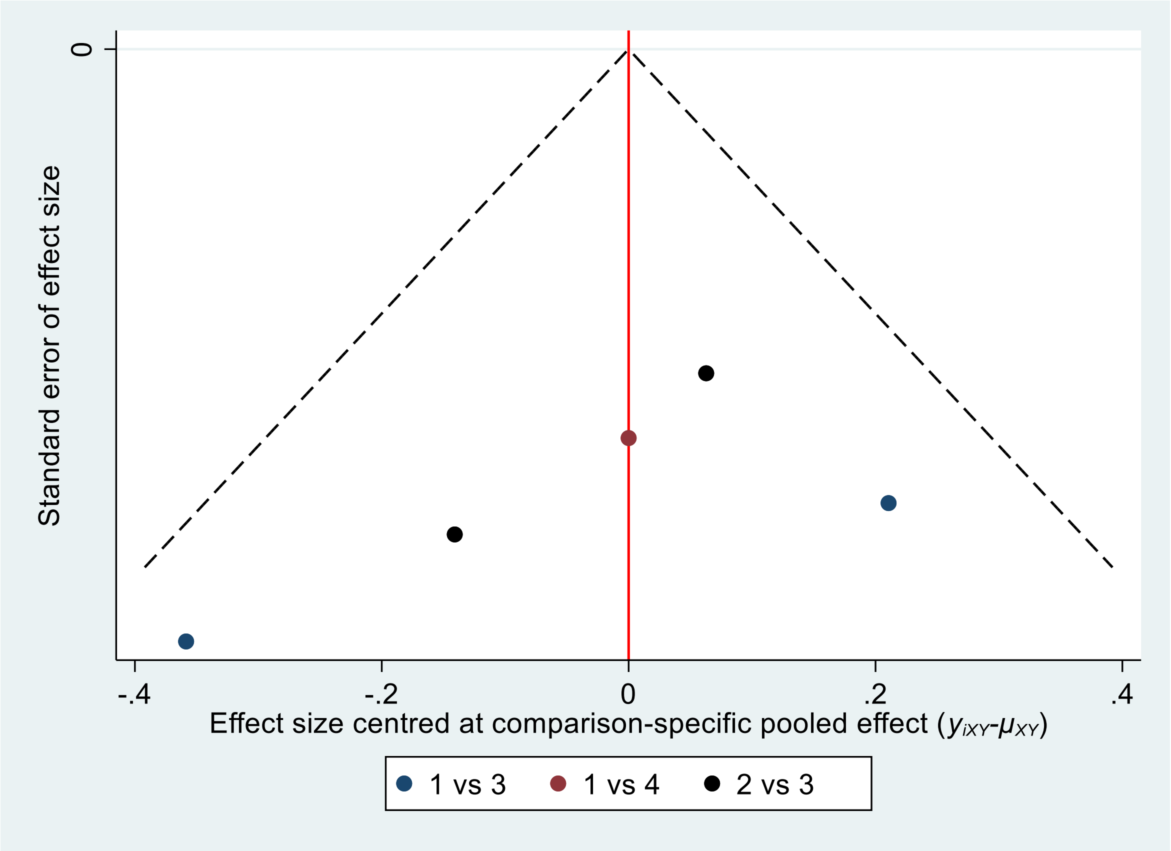

1= Waiting list, 2= Attention Control, 3= CBT, 4= Occupational Therapy

Targeted Secondary Anxiety

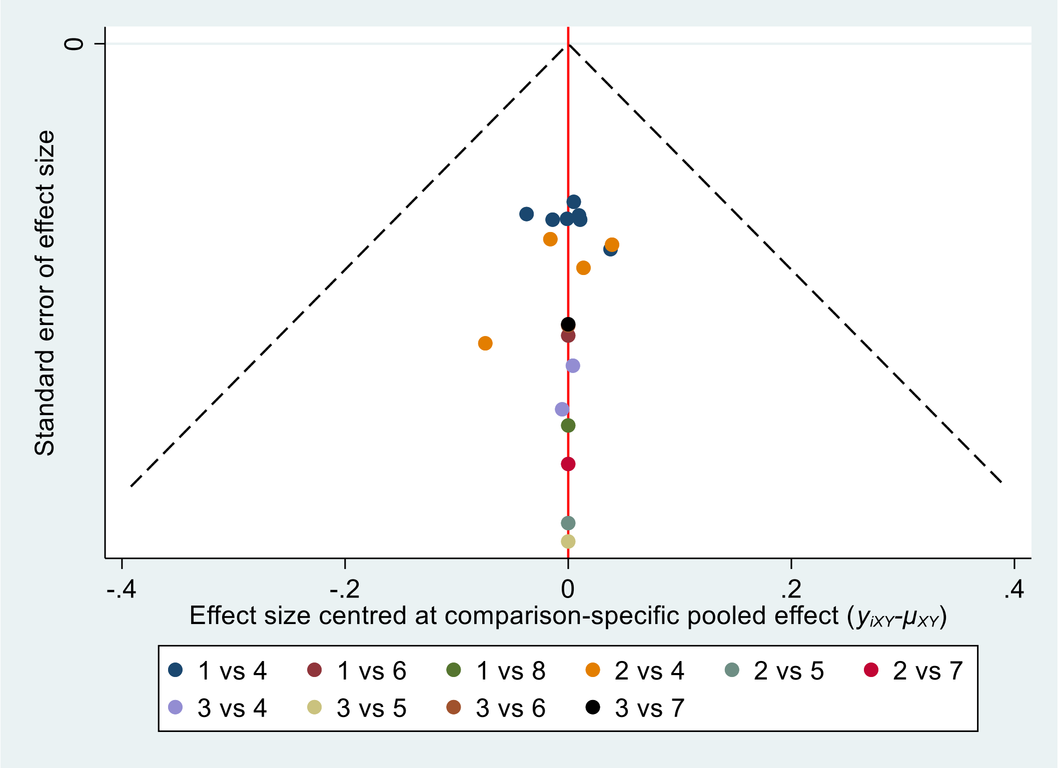

1 = No intervention, 2 = Waiting List, 3 = Attention control, 4= CBT, 5= Mindfulness/ relaxation, 6 =Bias modification, 7= Biofeedback, 8 = Exercise

Targeted Secondary Depression

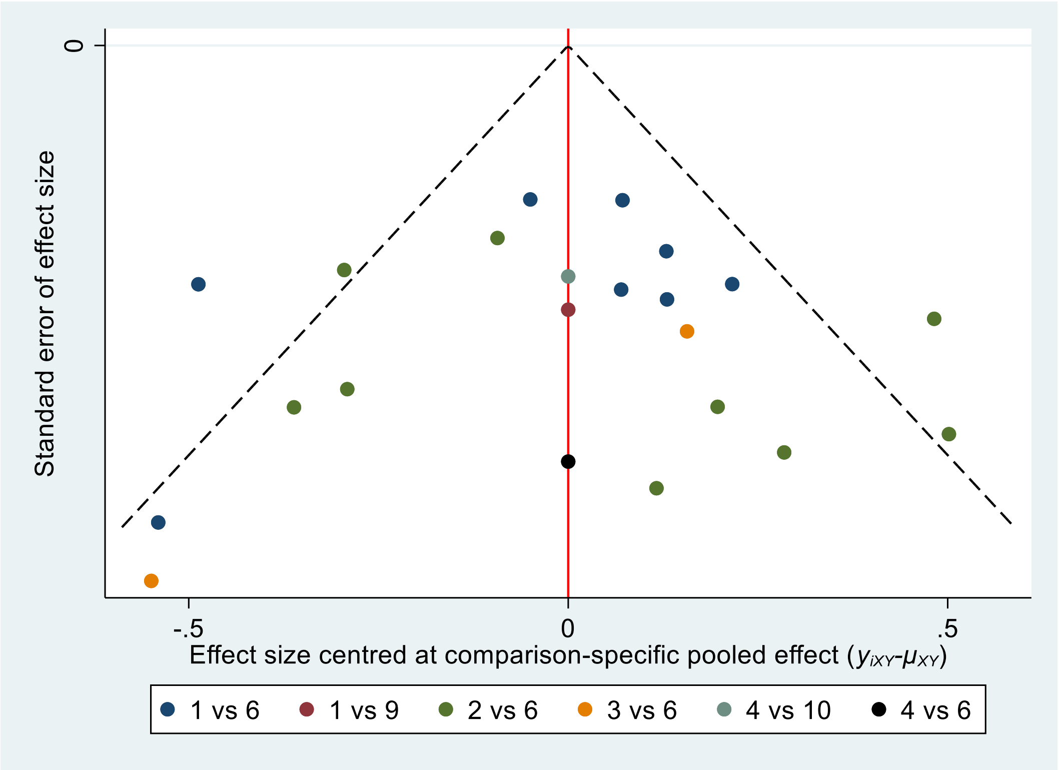

1 = No intervention, 2 = Waiting List, 3 = Usual curriculum, 4= Attention control, 6 =CBT
9 = Exercise, 10 = Bias modification

Universal Primary Anxiety

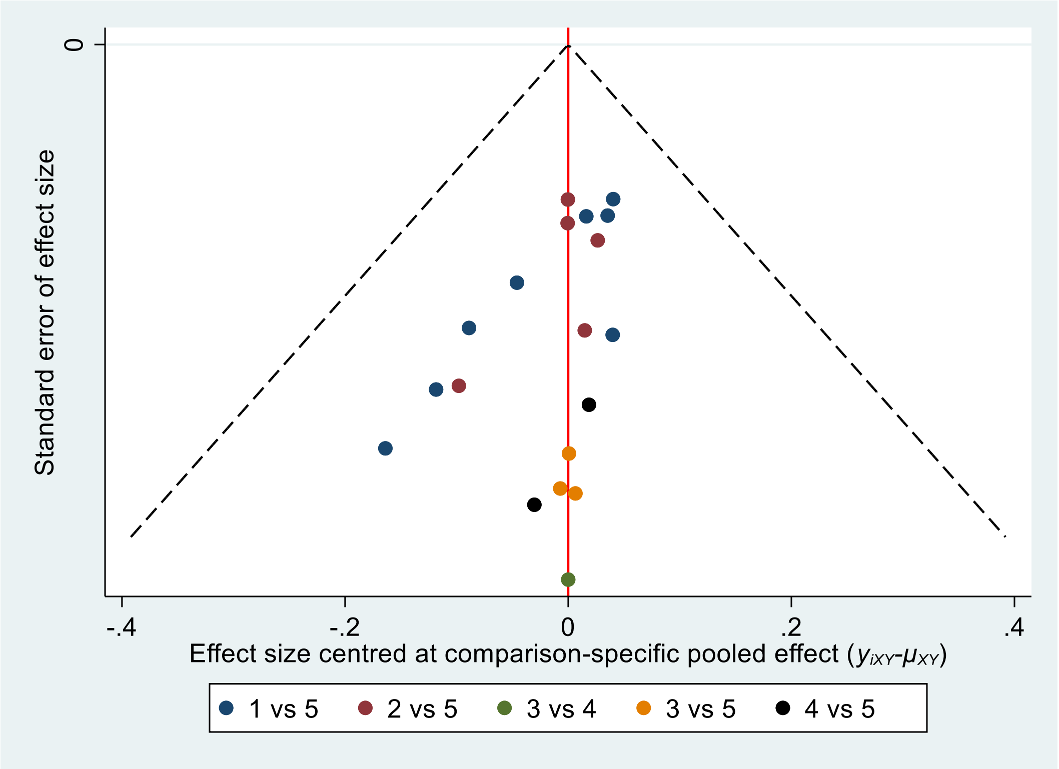

1 = Usual curriculum, 2 = Waiting List, 3 = No intervention, 4 = Attention control, 5= CBT

Universal Primary Depression

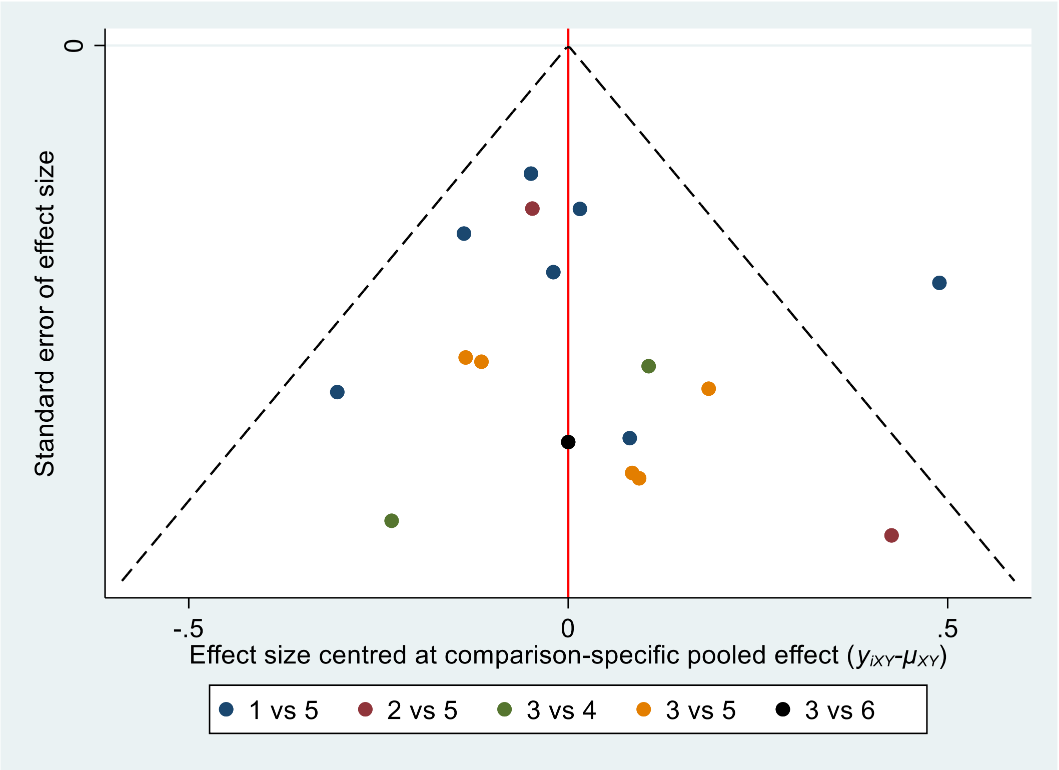

1 = Usual curriculum, 2 = Waiting List, 3 = No intervention, 4 = Attention control, 5= CBT, 6 = Behavioural

Universal Secondary Anxiety

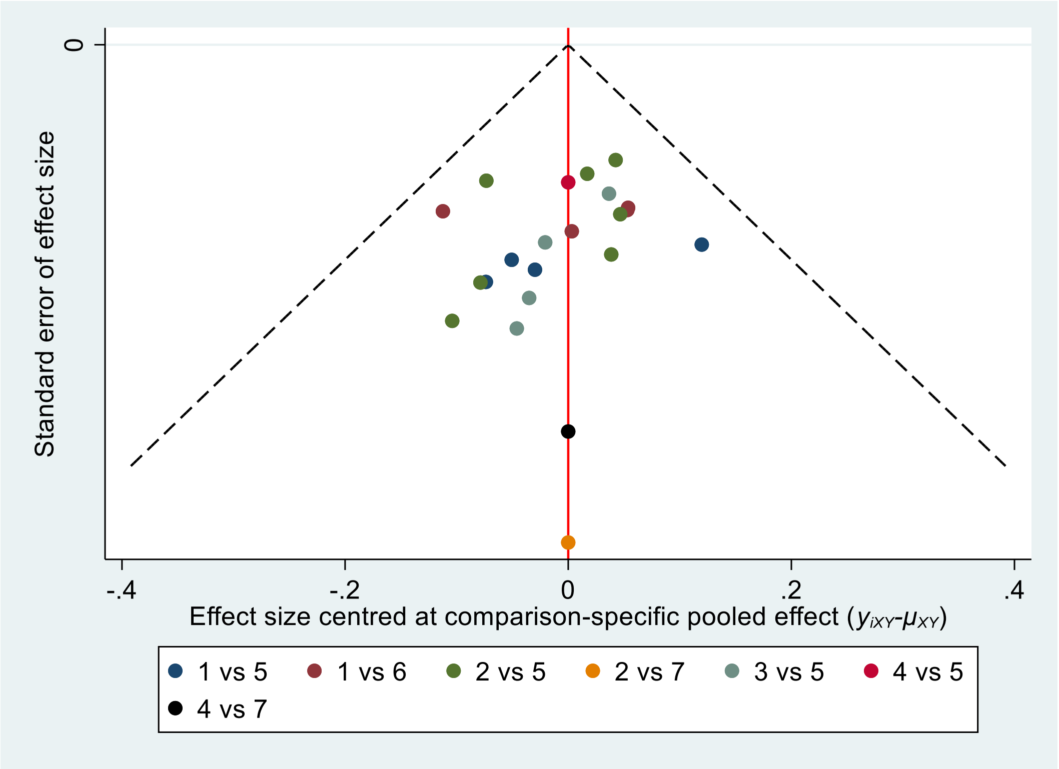

1 = Usual curriculum, 2 = Waiting List, 3 = No intervention, 4 = Attention control, 5= CBT, 6 = Third Wave, 7 = mindfulness/ relaxation

Universal Secondary Depression

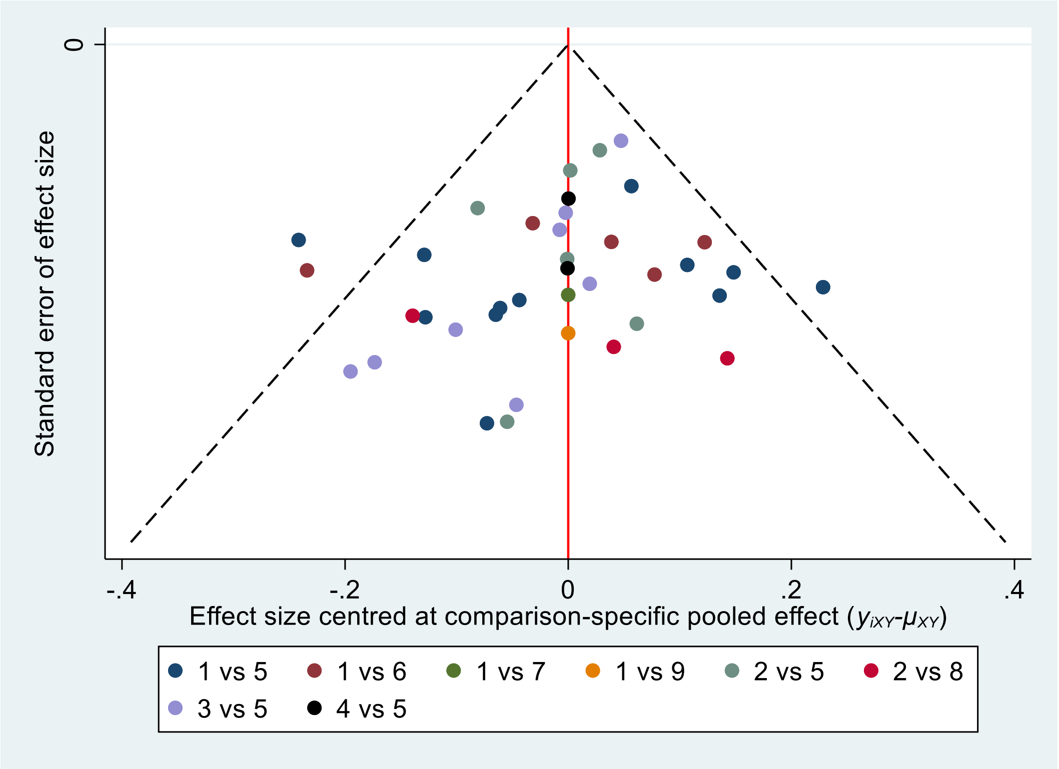

1 = Usual curriculum, 2 = Waiting List, 3 = No intervention, 4 = Attention control, 5= CBT, 6 = Third wave, 7 = IPT, 8 = IPT+CBT, 9 = Behavioural therapy.

Meta-regression and subgroup analyses

Mode of delivery

Interventions were categorised as being delivered face-to-face (in person) or via computer/ internet (multi-media). Across all networks the only intervention which varied by mode of delivery was CBT. To explore whether intervention effects were modified by mode of delivery we fitted a meta-regression model for CBT-F2F (covariate = 0) and CBT-MM (covariate = 1). This enables us to estimate the intervention effect for both CBT-F2F and CBT-MM. A random effects NMA model was fitted, but the regression coefficient for the covariate was assumed a fixed effect across studies. The between studies SD was assumed to be common for CBT-F2F and CBT-MM.

Results are reported for Universal Secondary settings only, as there was insufficient data available for meaningful analysis in other populations/settings.

Universal Secondary - depression

	SMD	95% CrIs	#studies
CBT F2F	-0.03	(-0.14 to 0.09)	18
CBT MM	-0.15	(-0.38 to 0.07)	5
SD:			0.15 (0.09 to 0.22)
Regression Coefficient:			-0.13 (-0.35 to 0.10)

Universal Secondary – anxiety

	SMD	95% CrIs	#studies
CBT F2F	-0.14	(-0.36 to 0.07)	11
CBT MM	-0.16	(-0.42 to 0.10)	5
SD:			0.12 (0.02 to 0.24)
Regression Coefficient			-0.02 (-0.26 to 0.23)

Facilitator delivering intervention

Interventions were categorised as being delivered by a teacher or a mental health professional (MHP). There was considerable variation within the classification of "mental health professional" and it should therefore be regarded as a simplification. Here MHP includes school counsellors, qualified psychotherapists, graduate and post-doctoral psychology students (which included general psychology, educational psychology or counselling psychology – if specified). In most studies, MHP were external to the educational setting, however this was not always the case.

Interventions which varied by person of delivery were CBT, third wave and mindfulness/ relaxation interventions. To explore whether intervention effects were modified by person delivering the intervention (teacher or mental health professional – MHP) we fitted a meta-regression model for Intervention-Teacher (0) and Intervention-MHP (1). This enables us to estimate the intervention effect at each value of the covariate, for each intervention, including multi-arm trials which compare the effect of both facilitators (e.g. Calear 2016) Where there were ≥2 interventions which were delivered by teacher or MHP, a random effects NMA model was fitted, and we assumed a hierarchical model for the regression coefficient across interventions (CBT, third wave and mindfulness/relaxation) where the regression coefficients were assumed to come from a normal distribution with mean (m.beta) and precision (tau.beta). The between studies SD was assumed to be common for each value of the covariate. We estimated a between intervention SD (sd.beta) for the covariate regression coefficients. Vague priors were specified.

Where only a single intervention varied by person delivering it, a fixed covariate effect (as for mode of delivery) was fitted.

Universal Secondary – Depression

	SMD	LCrI	UCrI	#studies
**CBT Teacher**	-0.07	-0.21	0.08	10
**CBT Mental**	-0.01	-0.15	0.14	8
**3rd Teacher**	-0.07	-0.37	0.23	1
**3rd Mental**	-0.02	-0.22	0.17	3
**CBT+IPT Teacher**	-0.21	-0.53	0.11	2
**CBT+IPT Mental**	-0.07	-0.43	0.31	1
**Between study SD**			0.14 (0.08 to 0.22)	
	**Mean**	**LCrI**	**UCrI**	
Regression Coefficient CBT	0.06	-0.10	0.22	
Regression Coefficient Third wave	0.05	-0.29	0.36	
Regression Coefficient CBT+IPT	0.13	-0.20	0.55	
m.beta	0.08	-0.53	0.72	
sd.beta	0.18	0.01	1.48	

Universal Secondary – Anxiety

	SMD	95% CrIs	#studies
**CBT Teacher**	-0.13	(-0.32 to 0.06)	9
**CBT MHP**	-0.18	(-0.42 to 0.03)	6
**3rd wave Teacher**	-0.10	(-0.37 to 0.19)	1
**3rd wave MHP**	0.10	(-0.11 to 0.29)	2
**MR Teacher**	-0.94	(-1.02 to 0.09)	1
**MR MHP**	-1.01	(-1.68 to -0.28)	1
**Between study SD**		0.10 (0.01 to 0.22)	
Regression Coefficient CBT		-0.05 (-0.24 to 0.10)	
Regression Coefficient 3rd wave		0.20 (-0.15 to 0.53)	
Regression Coefficient M/R		-0.48 (-1.39 to 0.22)	
m.beta		-0.11 (-1.30 to 0.94)	
sd.beta		0.58 (0.04 to 1.83)	

Universal Primary – Depression

	SMD	95% CrIs	#studies
**CBT Teacher**	-0.19	(-0.52 to 0.14)	4
**CBT MHP**	0.08	(-0.46 to 0.57)	5
Between study SD		0.30 (0.12 to 0.60)	
Regression Coefficient:		0.27 (-0.30 to 0.80)	

Universal Primary – Anxiety

	SMD	95% CrIs	#studies
**CBT Teacher**	-0.05	(-0.21 to 0.08)	6
**CBT MHP**	-0.18	(-0.42 to 0.00)	4
Between study SD		0.10 (0.01 to 0.26)	
Regression Coefficient:		-0.14 (-0.33 to 0.03)	

Targeted Secondary – Depression

	SMD	95% CrIs	#studies
**CBT Other**	-0.10	(-0.49 to 0.27)	14
**CBT MHP**	-0.30	(-0.67 to 0.06)	2
Between study SD:		0.35 (0.22 to 0.55)	
Regression Coefficient:		-0.20 (-0.52 to 0.13)	

Targeted Secondary – Anxiety

	SMD	95% CrIs	#studies
**CBT Other**	0.01	(-0.18 to 0.2)	2
**CBT MHP**	0.00	(-0.22 to 0.22)	7
**Bio Other**	-0.04	(-0.59 to 0.53)	1
**Bio MHP**	-0.27	(-0.88 to 0.32)	1
**Between study SD**:		0.08 (0.00 to 0.29)	
Regression Coefficient CBT		-0.01 (-0.23 to 0.2)	
Regression Coefficient Bio		-0.20 (-0.96 to 0.39)	
m.beta		-0.08 (-1.62 to 1.29)	
sd.beta		0.57 (0.00 to 1.89)	

Subgroup analysis: examining whether intervention effect is modified by the intended focus of the intervention

For each population, setting and outcome combination, intervention estimates are compared across three subgroups (1) interventions which aimed to prevent anxiety symptoms, (2) interventions which aimed to prevent only depressive symptoms and (3) interventions which aimed to prevent both anxiety and depressive symptoms. The interest here is whether interventions designed specifically to prevent one clinical disorder might still impact on the other. An intervention focussed on the prevention of anxiety may also report the effect on depressive symptoms, for example.

Key:

Focus: Anx = focus of intervention was prevention of anxiety, Dep = focus of intervention was prevention of depression, A+D = focus of intervention was prevention of both anxiety and depression

Comparison: where feasible the intervention effect estimate has been reported for the same intervention vs control comparison for each subgroup to allow for meaningful comparison.

Studies: number of studies per subgroup

SMD: standardised mean difference for each subgroup (and 95% credible intervals (CRIs)

SD: between study variation in effect for each subgroup (unless fixed effect analysis (FE))

Universal Secondary – Self-report depression

Focus	Comparison	#Studies	SMD	95% CrIs	SD	95% CrIs
ANX	CBT v NI	4	0.05	(-0.13 to 0.22)	0.04	(0.00 to 0.33)
DEP	CBT v NI	18	-0.16	(-0.37 to 0.05)	0.18	(0.10 to 0.30)
A+D	CBT v NI	10	0.05	(-0.33 to 0.47)	0.13	(0.01 to 0.32)

Universal Secondary – Self report anxiety

Focus	Comparison	#Studies	SMD	95% CrIs	SD	95% CrIs
ANX	CBT v NI	7	-0.11	(-0.4 to 0.16)	0.07	(0.00 to 0.39)
DEP	CBT v NI	4	0.00	(-5.26 to 5.25)	1.66	(0.12 to 4.74)
A+D	CBT v NI	10	-0.05	(-0.91 to 0.38)	0.16	(0.03 to 0.37)

Universal Primary - Self-report depression

Focus	Comparison	#Studies	SMD	95% CrIs	SD	95% CrIs
ANX	CBT v UC	2	0.18	(-0.06 to 0.41)	FE	
DEP	CBT v UC	6	-0.57	(-1.51 to 0.37)	0.34	(0.03 to 0.96)
A+D	CBT v UC	4	-0.16	(-0.42 to 0.13)	0.17	(0 to 0.78)

Universal Primary - Self report anxiety

Focus	Comparison	#Studies	SMD	95% CrIs	SD	95% CrIs
ANX	CBT v UC	9	-0.37	(-0.64 to -0.12)	0.08	(0.00 to 0.32)
DEP	CBT v NI	2	-0.31	(-0.61 to 0.00)	FE	
A+D	CBT v UC	4	0.04	(-0.16 to 0.27)	0.07	(0.00 to 0.61)

Targeted Secondary - Self-report depression

Focus	Comparison	#Studies	SMD	95% CrIs	SD	95% CrIs
ANX	CBT v WL	2	-0.21	(-0.49 to 0.08)	FE	
DEP	CBT v WL	17	-0.33	(-0.86 to 0.20)	0.38	(0.24 to 0.62)
A+D	CBT v WL	3	-0.67	(-3.65 to 2.33)	0.78	(0.00 to 4.56)

Targeted Secondary - Self report anxiety

Focus	Comparison	#Studies	SMD	95% CrIs	SD	95% CrIs
ANX	CBT v NI	8	0.13	(-0.95 to 1.18)	0.33	(0.03 to 1.34)
DEP	CBT v NI	3	0.00	(-0.15 to 0.16)	0.08	(0.00 to 0.26)
A+D	CBT v WL	3	-0.21	(-1.27 to 0.84)	0.45	(0.01 to 2.66)

Targeted Primary - Self report anxiety

Focus	Comparison	#Studies	SMD	95% CrIs	SD	95% CrIs
ANX	CBT v WL	7	-0.16	(-0.41 to 0.09)	0.14	(0.00 to 0.48)
A+D	CBT v WL	4	-1.43	(-5.47 to 2.60)	1.19	(0.02 to 4.52)

Comparison of results from recent meta-analyses and the results from the network meta-analysis (DeCoDA).

	**Universal Depression**	**Universal Anxiety**	**Targeted Depression^**	**Targeted Anxiety^**
DeCoDA NMA (UC vs CBT)	-0.04 (-0.16 to 0.07)	-0.14 (-0.34 to 0.04)	-0.21 (-0.58 to 0.13)	0.03 (-0.11 to 0.16)
DeCoDA NMA (lumped ctrl vs CBT only)*	-0.09 (-0.16 to -0.01)	-0.08 (-0.14 to -0.02)	-0.24 (-0.45 to -0.04)	-0.15 (-0.29 to -0.02)
Hetrick 2016	-0.11 (-0.17 to -0.05)	NA	-0.32 (-0.42 to -0.23)	NA
Stockings 2016	-0.11 (-0.16 to -0.05)	-0.16 (-0.27 to -0.06)	-0.33 (-0.46 to -0.20)	-0.01 (-0.27 to 0.26)
Werner-Seidler 2017	0.19 (0.14 to 0.24)	0.19 (0.13 to 0.26)	0.32 (0.23 to 0.41)	0.22 (0.09 to 0.34)
Johnstone 2018	0.17 (0.06 to 0.28)	0.09 (-0.07 to 0.26)	NA	NA
Rasing 2017	NA	NA	-0.25 (-0.38 to -0.12)	-0.19 (-0.36 to 0.03)

* Combined primary and secondary settings and 4 control groups 'lumped' to form a single comparator

^No intervention vs CBT

1. Hetrick SE, Cox GR, Witt KG, Bir JJ, Merry SN. Cognitive behavioural therapy (CBT), third-wave CBT and interpersonal therapy (IPT) based interventions for preventing depression in children and adolescents. The Cochrane database of systematic reviews. 2016(8):Cd003380.
2. Stockings EA, Degenhardt L, Dobbins T, Lee YY, Erskine HE, Whiteford HA, et al. Preventing depression and anxiety in young people: a review of the joint efficacy of universal, selective and indicated prevention. Psychological medicine. 2016;46(1):11-26.
3. Werner-Seidler A, Perry Y, Calear AL, Newby JM, Christensen H. School-based depression and anxiety prevention programs for young people: A systematic review and meta-analysis. Clinical psychology review. 2017;51:30-47.
4. Johnstone KM, Kemps E, Chen J. A Meta-Analysis of Universal School-Based Prevention Programs for Anxiety and Depression in Children. Clinical child and family psychology review. 2018;21(4):466-81.
5. Rasing SPA, Creemers DHM, Janssens JMAM, Scholte RHJ. Depression and Anxiety Prevention Based on Cognitive Behavioral Therapy for At-Risk Adolescents: A Meta-Analytic Review. Frontiers in Psychology. 2017;8(1066).

Post-hoc exploration of control groups and influence on findings

The findings reported in the main paper contrast with recent meta-analyses. Although our inclusion criteria differ slightly, an important difference is that we analysed four distinct control conditions - attention control, wait list, no intervention and usual curriculum – rather than a single 'lumped' control, as is necessary in standard meta-analyses. In standard meta-analysis the effect of different control groups might be investigated using meta-regression analyses. NMA can be thought of as a form of meta-regression but has the advantage that relative effects are estimated simultaneously across the network of comparisons. If there are no loops in a network of evidence, then splitting the different control interventions into separate nodes would be equivalent to a meta-regression with control types as covariates. However, if there are multiple active interventions in an NMA, the effect of control type is estimated jointly across all active interventions. Our decision to *a priori* split control groups was based on the psychotherapeutic literature, where it is established that control group choice contributes to differences in effect size estimate. In particular, it has been suggested that waiting list exaggerates the effectiveness of psychological treatments, and may be a 'nocebo'

To explore the impact of our decision to 'split' control type into four groups, we ran post-hoc analyses 'lumping' them into a single comparator. We also combined primary and secondary settings to emulate the approach taken in previously published reviews. When control conditions are combined, our results were consistent with previous reviews e.g. intervention effects for CBT vs 'control' now indicate a beneficial effect of CBT in every network at post-intervention (Appendix). This suggests that previously observed effects of school-based interventions may be driven by the differential effect of control groups being masked by 'lumping' and by combining primary and secondary school settings in one analysis. Our post-hoc findings provide some evidence that, especially for targeted interventions, lumping of control conditions exaggerates the beneficial effects of the active interventions, due to the control effect being brought down by the inclusion of waiting list. However, there is no evidence of a control group effect for universal interventions. In the appendix we also report SMDs for each separate control relative to CBT, mindfulness/relaxation and third wave interventions. Further research into the importance of control conditions for preventative interventions should be considered.

Intervention	Universal interventions. Outcome: Depression			
	(a) Secondary settings only	(b) Primary settings only	(c) Primary + secondary combined	(d) Primary + secondary settings (lumped ctrl)
**Usual curriculum**	Reference	Reference	Reference	Lumped control
**No intervention**	0.03 95% CrI (-0.15 to 0.21)	0.13 95% CrI (-0.40 to 0.65)	0.06 95% CrI (-0.10 to 0.23)	
**Waitlist**	0.00 95% CrI (-0.19 to 0.19)	-0.09 95% CrI (-0.77 to 0.54)	-0.01 95% CrI (-0.20 to 0.18)	
**Attention control**	0.07 95% CrI (-0.12 to 0.25)	-0.07 95% CrI (-0.79 to 0.62)	0.04 95% CrI (-0.15 to 0.23)	
**CBT**	-0.04 95% CrI (-0.16 to 0.07)	-0.13 95% CrI (-0.44 to 0.17)	-0.07 95% CrI (-0.17 to 0.03)	-0.09 95% CrI (-0.16 to -0.01)
**Third wave**	-0.03 95% CrI (-0.21 to 0.14)	*Not in network*	-0.04 95% CrI (-0.23 to 0.16)	-0.04 95% CrI (-0.22 to 0.15)
**CBT+IPT**	-0.19 95% CrI (-0.46 to -0.08)	*Not in network*	-0.20 95% CrI (-0.49 to 0.08)	-0.21 95% CrI (-0.44 to 0.02)
**IPT**	-0.03 95% CrI (-0.36 to 0.29)	*Not in network*	-0.05 95% CrI (-0.41 to 0.32)	-0.06 95% CrI (-0.41 to 0.29)
**Psycho-education**	-0.13 95% CrI (-0.49 to 0.22)	*Not in network*	-0.13 95% CrI (-0.54 to 0.27)	-0.13 95% CrI (-0.52 to 0.25)
**Behaviour therapy**	-0.02 95% CrI (-0.40 to 0.37)	-0.10 95% CrI (-1.04 to 0.80)	-0.04 95% CrI (-0.38 to 0.29)	-0.06 95% CrI (-0.39 to 0.26)
**SD**	0.15 [95% CrI (0.10 to 0.22)]	0.32 [95% CrI (0.18 to 0.59)]	0.18 (95% CrI [0.13 to 0.24])	0.17 (95% CrI [0.12 to 0.23])
**Residual deviance (DIC)**	78.3 (172.3)	28.87 (98.72)	107.3 (268.2)	108.5 (267.4)

Comparison of intervention effect estimates across secondary (a) and primary (b) settings, versus combining both settings (c) and conflating all controls to form a single control comparator (d). 'split' control model refers to the standard NMA model here, where four separate control groups are used.

Highlighting shows interventions attaining conventional statistical significance

Intervention	Universal interventions. Outcome: Anxiety

	(a) Secondary settings only		(b) Primary settings only		(c) Primary + secondary settings		(d) Primary + secondary settings (lumped ctrl)	
**Usual curriculum**	Reference		Reference		Reference		Lumped control	
**No intervention**	-0.07	95% CrI (-0.34 to 0.20)	0.23	95% CrI (-0.15 to 0.60)	0.02	95% CrI (-0.16 to 0.21)		
**Waitlist**	-0.05	95% CrI (-0.28 to 0.18)	0.02	95% CrI (-0.20 to 0.22)	0.00	95% CrI (-0.14 to 0.13)		
**Attention control**	-0.15	95% CrI (-0.34 to 0.04)	-0.17	95% CrI (-0.51 to 0.17)	-0.13	95% CrI (-0.36 to 0.07)		
**CBT**	-0.15	95% CrI (-0.34 to 0.04)	-0.07	95% CrI (-0.23 to 0.05)	-0.09	95% CrI (-0.20 to 0.00)	-0.08	95% CrI (-0.14 to -0.03)
**Third wave**	0.03	95% CrI (-0.14 to 0.20)	*Not in network*		0.03	95% CrI (-0.12 to 0.00)	0.03	95% CrI (-0.10 to 0.16)
**Mindfulness/ relaxation**	-0.65	95% CrI (-1.14 to -0.19)	*Not in network*		-0.62	95% CrI (-1.04 to -0.22)	-0.52	95% CrI (-0.89 to -0.16)
**SD**	0.11 (95% CrI [0.02 to 0.23])		0.10	95% CrI (0.01 to 0.26)	0.10 (95% CrI [0.02 to 0.17])		0.10 (95% CrI [0.02 to 0.18])	
**Residual deviance (DIC)**	49.52 (102.0)		37.42 (145.8)		86.75 (243.1)		91.97 (242.6)	

iii. Comparison of intervention effect estimates across secondary (a) and primary (b) settings, versus combining both settings (c) and conflating all controls to form a single control comparator (d). Highlighting shows interventions attaining conventional statistical significance

**Intervention**	**Targeted interventions. Outcome: Depression**

	(a) Secondary settings only		(b) Primary settings only		(c) Primary + secondary settings		(d) Primary + secondary settings (lumped ctrl)	
**Usual curriculum**	0.04	95% CrI (-0.72 to 0.81)	*Not in network*		0.03	95% CrI (-0.69 to 0.76)	Lumped control	
**No intervention**	Reference		*Not in network*		Reference			
**Wait list**	0.22	95% CrI (-0.28 to 0.70)	Reference		0.23	95% CrI (-0.21 to 0.66)		
**Attention control**	-0.81	95% CrI (-1.82 to 0.18)	-0.72	95% CrI (-3.56 to 2.10)	-0.56	95% CrI (-1.15 to 0.01)		
**CBT**	-0.22	95% CrI (-0.58 to 0.13)	-0.48	95% CrI (-2.49 to 1.50)	-0.21	95% CrI (-0.56 to 0.12)	-0.24	95% CrI (-0.45 to -0.04)
**Interpersonal Therapy**	-0.65	95% CrI (-1.50 to 0.16)	*Not in network*		-0.64	95% CrI (-1.44 to 0.13)	-0.68	95% CrI (-1.49 to 0.12)
**Third wave**	-3.74	95% CrI (-4.90 to -2.59)	*Not in network*		-3.73	95% CrI (-4.84 to -2.65)	-3.76	95% CrI (-4.92 to -2.61)
**Psycho-support**	-0.02	95% CrI (-0.63 to 0.66)	*Not in network*		0.03	95% CrI (-0.58 to 0.62)	0.00	95% CrI (-0.60 to 0.59)
**Bias modification**	-0.90	95% CrI (-2.21 to 0.40)	*Not in network*		-0.65	95% CrI (-1.63 to 0.32)	-0.09	95% CrI (-0.96 to 0.79)
**Exercise**	-0.28	95% CrI (-1.13 to 0.58)	*Not in network*		-0.28	95% CrI (-1.07 to 0.52)	-0.28	95% CrI (-1.17 to 0.61)
**Occupational therapy**	*Not in network*		-0.10	95% CrI (-2.94 to 2.71)	0.14	95% CrI (-0.77 to 1.03)	-0.10	95% CrI (-0.98 to 0.78)
**Psycho-education**	0.12	95% CrI (-0.50 to 0.72)	*Not in network*		0.12	95% CrI (-0.46 to 0.69)	0.09	95% CrI (-0.47 to 0.64)
**SD**	0.38 (0.25 to 0.58)		0.60 (0.08 to 3.80)		0.35 (95% CrI [0.24 to 0.53])		0.40 (95% CrI [0.29 to 0.57])	
**Residual deviance (DIC)**	57.62 (176.6)		10.29 (38.1)		67.9 (213.3)		67.3 (213.0)	

Comparison of intervention effect estimates across secondary (a) and primary (b) settings, versus combining both settings (c) and conflating all controls to form a single control comparator (d). Highlighting shows interventions attaining conventional statistical significance

**Intervention**	**Targeted interventions. Outcome: Anxiety**

	(a) Secondary settings only		(b) Primary settings only		(c) Primary + secondary settings		(d) Primary + secondary settings (lumped control)	
**No intervention**	Reference		*Not in network*		Reference		Lumped control	
**Waitlist**	0.30	95% CrI (0.09 to 0.53)	Reference		0.37	95% CrI (0.11 to 0.65)		
**Attention control**	-0.09	95% CrI (-0.39 to 0.22)	-0.35	95% CrI (-1.05 to 0.33)	0.04	95% CrI (-0.24 to 0.32)		
**CBT**	0.03	95% CrI (-0.11 to 0.16)	-0.38	95% CrI (-1.50 to 0.72)	0.03	95% CrI (-0.18 to 0.24)	-0.15	95% CrI (-0.29 to -0.02)
**Biofeedback**	-0.18	95% CrI (-0.55 to 0.21)	-0.23	95% CrI (-1.30 to 0.84)	-0.03	95% CrI (-0.41 to 0.33)	-0.20	95% CrI (-0.57 to 0.15)
**Psycho-support**	1.08	95% CrI (0.52 to 1.64)	*Not in network*		1.08	95% CrI (0.40 to 1.76)	0.90	95% CrI (0.16 to 1.64)
**Mindfulness/ relaxation**	0.03	95% CrI (-0.42 to 0.48)	*Not in network*		0.11	95% CrI (-0.39 to 0.62)	-0.08	95% CrI (-0.6 to 0.42)
**Bias modification**	-0.17	95% CrI (-0.45 to 0.11)	*Not in network*		-0.11	95% CrI (-0.48 to 0.26)	-0.18	95% CrI (-0.58 to 0.22)
**Exercise**	-0.47	95% CrI (-0.86 to -0.09)	*Not in network*		-0.47	95% CrI (-0.99 to 0.05)	-0.47	95% CrI (-1.09 to 0.15)
**Occupational therapy**	*Not in network*		0.11	95% CrI (-0.91 to 1.14)	0.48	95% CrI (-0.08 to 1.06)	0.11	95% CrI (-0.49 to 0.71)
**SD**	0.06 (0.00 to 0.21)		0.42 (0.21 to 0.89)		0.19 (95% CrI [0.06 to 0.33])		0.25 (95% CrI [0.14 to 0.39])	
**Residual deviance (DIC)**	36.29 (105.8)		23.86 (61.54)		65.32 (173.4)		63.55 (173.9)	

Comparison of intervention effect estimates across secondary (a) and primary (b) settings, versus combining both settings (c) and conflating all controls to form a single control comparator (d). Highlighting shows interventions attaining conventional statistical significance

Relative effects of active interventions compared to separate controls for each of the 4 depression and anxiety networks: NMA

Universal Secondary Depression

		AC		UC		NI		WL
**CBT**	-0.11	95% CrI (-0.27 to 0.01)	-0.04	95% CrI (-0.16 to 0.07)	-0.07	95% CrI (-0.21 to 0.07)	-0.04	95% CrI (-0.20 to 0.11)
**CBT+IPT**	-0.25	95% CrI (-0.51 to 0.00)	-0.19	95% CrI (-0.46 to 0.08)	-0.22	95% CrI (-0.50 to 0.06)	-0.19	95% CrI (-0.41 to 0.04)

Universal Primary Depression

		AC		UC		NI		WL
**CBT**	-0.06	95% CrI (-0.68 to 0.59)	-0.13	95% CrI (-0.44 to 0.17)	-0.26	95% CrI (-0.69 to 0.17)	-0.04	95% CrI (-0.60 to 0.56)

Targeted Secondary Depression

		AC		UC		NI		WL
**CBT**	0.59	95% CrI (-0.34 to 1.53)	-0.26	95% CrI (-0.95 to 0.41)	-0.22	95% CrI (-0.58 to 0.13)	-0.44	95% CrI (-0.77 to -0.10)
**Third wave**	-2.93	95% CrI (-4.37 to -1.49)	-3.78	95% CrI (-5.08 to -2.50)	-3.74	95% CrI (-4.90 to -2.59)	-3.96	95% CrI (-5.11 to -2.81)

Targeted Primary Depression

		AC		WL
**CBT**	0.25	95% CrI (-1.76 to 2.21)	-0.48	95% CrI (-2.49 to 1.50)

Universal and targeted populations: depression outcome. Comparisons from random effects network meta-analysis, assuming consistency.

SMDs and 95% credible intervals by population, setting and outcome.

AC attention control; UC usual curriculum; NI no intervention; WL wait list. CBT cognitive behavioural therapy;

Universal Secondary Anxiety

		AC		UC		NI		WL
**CBT**	0.01	95% CrI (-0.25 to 0.30)	-0.15	95% CrI (-0.34 to 0.04)	-0.07	95% CrI (-0.27 to 0.11)	-0.10	95% CrI (-0.24 to 0.03)

**Third wave**	0.18	95% CrI (-0.17 to 0.59)	0.03	95% CrI (-0.14 to 0.20)	0.10	95% CrI (-0.22 to 0.42)	0.08	95% CrI (-0.21 to 0.37)
**M/R**	-0.50	95% CrI (-0.90 to -0.10)	-0.65	95% CrI (-1.14 to -0.19)	-0.58	95% CrI (-1.07 to -0.11)	-0.65	95% CrI (-1.14 to -0.19)

Universal Primary Anxiety

		**AC**		**UC**		**NI**		**WL**
**CBT**	0.10	95% CrI (-0.22 to 0.40)	-0.07	95% CrI (-0.23 to 0.05)	-0.30	95% CrI (-0.65 to 0.05)	-0.10	95% CrI (-0.26 to 0.06)

Targeted Secondary Anxiety

		**AC**		**NI**		**WL**
**CBT**	0.12	95% CrI (-0.17 to 0.40)	0.03	95% CrI (-0.11 to 0.16)	-0.28	95% CrI (-0.45 to -0.11)
**M/R**	0.12	95% CrI (-0.31 to 0.55)	0.03	95% CrI (-0.42 to 0.48)	-0.28	95% CrI (-0.71 to 0.15)

Targeted Primary Anxiety

		**AC**		**WL**
**CBT**	-0.03	95% CrI (-0.54 to 0.49)	-0.38	95% CrI (-0.84 to 0.07)

Relative effects of active interventions relative to distinct controls for each of the 4 anxiety networks.

SMDs and 95% credible intervals by population, setting and outcome. Comparisons from random effects network meta-analysis, assuming consistency.

AC attention control; UC usual curriculum; NI no intervention; WL wait list
CBT cognitive behavioural therapy; M/R mindfulness/relaxation-based interventions.

References to studies awaiting screening

Unable to locate full text

Boogar Isaac, Rahimian (2012) Effectiveness of the Teasdale Cognitive Therapy on depression reduction in guidance and high school students. [Farsi (Iranian)]. *Psychological research* 14; 25-40

Dadsetan, Parirokh Anari, and Asieh Sedghpour Bahrain, Saleh. (2008) "Social anxiety disorders and drama-therapy". *Journal of Iranian psychologists* 4(14) 115-123

Diner, Md (1978) “The differential effects of meditation and systematic desensitization on specific and general anxiety” *Dissertation abstracts international* (found on CENTRAL database).

Kahn, RHC (1989) “The effect of a group support intervention program on depression, social adjustment, and self-esteem of adolescents in an overseas American International School” *CENTRAL database*, The Catholic University of America

Ma Hui-xia Liu, Mei-ting Zhang, Fei-yi (2012) “Improving the academic emotions of high-school students by rational-emotive educational mode”. *Chinese journal of clinical psychology.* 20 (1) 116-119

Mirzamani SM, Azvar F, Dolatshahi B, Askari A. (2012) “Efficacy of life skills training on reduce depressive symptoms in student population” [اثربخشي آموزش مهارت هاي زندگي بر كاهش علايم افسردگي دانش آموزان]. *Journal of Research in Behavioural Sciences* ;10(2):124-32.

Moharreri F, & Yazdi A. (2017) “Evaluation of the Effectiveness of the Friends for Life Program on Children’s Anxiety and Depression”. *Iran J Psychiatry* 2017; 12: 4: 272-280

Short CA (1993) “Universal prevention program for anxiety symptoms in school aged children: Taming Worry Dragons” University of British Colombia. Dissertation abstracts International

Zou, Min and Han, Ren-Sheng (2008) “Attributive training in junior school students with high-level anxiety” *Chinese mental health journal.* 3(22) 358-371 Abstract available from http://en.cnki.com.cn /Article_en/ CJFDTOTAL-ZXWS200805016.htm

No author details. (2012) “Effectiveness of group cognitive-emotional skills training on improvement of anxiety management in primary school children” *Iranian journal of psychiatry*. 7 (4). (Found in CENTRAL database)

Awaiting classification

St Onge J, Stephenson, R and Kumar, BS (2016) “Validation of the FRIENDS anxiety prevention program for children in Canada” *Canadian Journal of Community Mental Health* 35 (3) 25-40

Silvestri L, Dantonio, M and Eason, S (1996). “The effects of a self-development program and relaxation/imagery training on the anxiety levels of at-risk fourth grade students” *Journal of instructional psychology* 23; 167-173

Warren R, Smith G and Velten E. (1984) Rational-emotive therapy and the reduction of interpersonal anxiety in junior high school students. *Adolescence* 19: 893-902

Eimecke S, Pauschardt J, and Mattejat F. (2010) “Prevention of childhood anxiety and depression: Efficacy of an additional parent training program”. [German] Verhaltenstherapie. 20 (3) 193-200

Petersen A, Leffert A, Graham B, Alwin J, Ding S. (1997) Promoting mental health during the transition into adolescence. In: Schulenberg J, Maggs JL, Hierrelmann AK editor(s). Health Risks and Developmental Transitions During Adolescence. New York, NY: Cambridge University Press, 1997:471-97.

Vazquez FL, Torres A, Blanco V, Diaz O, Otero P, and Hermida E. (2012) “Comparison of relaxation training with a cognitive-behavioural intervention for indicated prevention of depression in university students: a randomized controlled trial” *Journal of Psychiatric Research.* 46, Issue 11, 1456-1463

Kim, K. B. and Cohen, S. M. and Oh, H. K. and Sok, S. R. (2004) “The effects of meridian exercise on anxiety, depression, and self-esteem of female college students in Korea” *Holistic Nursing Practice* 18 (5): 230-4 (Not RCT)

Tomyn, Justin D. et al. “A Comprehensive Evaluation of a Universal School-Based Depression Prevention Program for Adolescents.” Journal of abnormal child psychology 44 8 (2016): 1621-1633 . (not RCT)

Conference abstracts

Rezaei Ghalechi, E. and Sadeghi Movahhed, F. (2013) “Teaching coping skills affects on decreasing mental disorders symptoms of students”. *European psychiatry Conference: 21st European Congress of Psychiatry, EPA 2013. Nice France.* Conference Publication: (var.pagings). 28

Tze-Chun, T. and Shih-Yin, H. (2010) “Efficacy of school-based interpersonal psychotherapy to adolescents of early detected depressive and suicide ideations: Randomized control study” [conference abstract] *Early intervention in psychiatry [abstracts of the 7th international conference on early psychosis - early psychoses: A lifetime perspective.* 29 Nov - 1 Dec 2010; Amsterdam, Netherlands]

Davis, H. (1996) “Youth Clubs: outcome of a community-based intervention for prevention of mental health disorders in adolescence” European child psychiatry research group - invitational meeting; 5-7 September; Oslo (found on CENTRAL database).

Awaiting translation

Tsutsumi, A. (2015) Effects of a psycho-educational program for preventing depression in junior high and high school students. *Japanese journal of educational psychology.* 63 (3) 323-337

Papers possibly linked to included studies

Briere, F. N. and Rohde, P. and Stice, E. and Morizot, J. (2016) “Group-Based Symptom Trajectories in Indicated Prevention of Adolescent Depression” *Depression & Anxiety;* 33 (5); 444-51 Linked to Rohde 2014?

Brunwasser, S. M. and Freres, D. R. and Gillham, J. E. (2018) “Youth Cognitive-Behavioral Depression Prevention: Testing Theory in a Randomized Controlled Trial” *Cognitive Therapy and Research*. Linked to Gillham 2007?

Benas, J. S., McCarthy, A. E., Haimm, C. A., Huang M, Gallop R, and Young, JF (2016) “The Depression Prevention Initiative: Impact on Adolescent Internalizing and Externalizing Symptoms in a Randomized Trial” *Journal of Clinical Child & Adolescent Psychology.* Linked to Young 2016?

Marchand E, Ng J, Rohde P., and Stice E. (2010) “Effects of an indicated cognitive-behavioral depression prevention program are similar for Asian American, Latino, and European American adolescents” *Behaviour Research & Therapy* 48(8) 821-5, Linked to Stice 2006?

Muller, S. and Rohde, P. and Gau, J. M. and Stice, E. (2015) “Moderators of the effects of indicated group and bibliotherapy cognitive behavioral depression prevention programs on adolescents' depressive symptoms and depressive disorder onset” *Behaviour Research & Therapy*. Linked to Stice 2006?

Gau, J. M. and Stice, E. and Rohde, P. and Seeley, J. R. (2012) “Negative life events and substance use moderate cognitive behavioral adolescent depression prevention intervention” *Cognitive Behaviour Therapy*. 41 (3); 241-50. Linked to Stice 2006 or Rohde 2014?

Changes from protocol

1. The protocol stated that the relevant age range for inclusion was 5-25. Due to variation in international school starting ages the lower age limit was changed to age 4. This was operationalised as follows: studies were included in which most children were aged 5 or greater, or where the mean age was ~5 with a "small" SD. Studies in which the majority of children are <4 were excluded. The original upper age limit was selected to allow sufficient time for multiple follow ups in tertiary settings but was difficult to operationalise during pilot extraction, as studies had a wide age range at baseline spanning the upper age limit. For example, Rohde 2016 included a range from 18 to 28 years old at baseline. As such, this was modified to include studies in which the majority of participants were <19 at baseline. To a lesser degree there is also some variation in average starting age of tertiary education, typically varying between 17 and 19 years of age.

2. We planned to analyse 'inequality' as a main outcome, however this was not possible. As such we planned post hoc subgroup analyses by socio-economic status, gender and ethnicity.
3. We planned to conduct meta-regression by intervention length and/ or intensity, where intensity is defined as total session time (number of sessions * length in minutes). However, we determined that this would not be meaningful in a NMA with differing classes of intervention.
4. We conducted a post-hoc exploration of the effect of control groups on the overall results from the NMA

5. Protocol Clarification: Educational settings were divided into Primary, secondary and tertiary for the purposes of analysis. This was not made explicit in the original protocol which implied that the intervention should be delivered in one of these settings.

References to included studies

1. Ahlen J, Lenhard F, Ghaderi A. Universal Prevention for Anxiety and Depressive Symptoms in Children: A Meta-analysis of Randomized and Cluster-Randomized Trials. The journal of primary prevention 2015; 36(6): 387-403.
2. Anticich Sarah AJBP, M. Silverman, Wendy Lacherez, Philippe Gillies, Robyn. The prevention of childhood anxiety and promotion of resilience among preschool-aged children: A universal school based trial. Advances in school mental health promotion 2013; 6(2): 93-121.
3. Araya R, Fritsch R, Spears M, et al. School intervention to improve mental health of students in Santiago, Chile: a randomized clinical trial. Jama, Pediatr 2013; 167(11): 1004-10.
4. Attwood M, Meadows S, Stallard P, Richardson T. Universal and targeted computerised cognitive behavioural therapy (Think, Feel, Do) for emotional health in schools: Results from two exploratory studies. Child and adolescent mental health 2012; 17(3): 173-8.
5. Aune T, Stiles TC. Universal-based prevention of syndromal and subsyndromal social anxiety: A randomized controlled study. J Consult Clin Psychol 2009; 77(5): 867-79.
6. Baker SB, Butler JN. Effects of preventive cognitive self-instruction training on adolescent attitudes, experiences, and state anxiety. J Prim Prev 1984; 5(1): 17-26.
7. Barrett P, Turner C. Prevention of anxiety symptoms in primary school children: preliminary results from a universal school-based trial. Br J Clin Psychol 2001; 40(Pt 4): 399-410.
8. Barrett P, Lock S, Farrell L. Developmental differences in universal preventive intervention for child anxiety. Clinical child psychology and psychiatry 2005; 10(4): 539-55.
9. Barry M, Murphy M, O'Donovan H. Assessing the effectiveness of a cognitive behavioural group coaching intervention in reducing symptoms of depression among adolescent males in a school setting. International Coaching Psychology Review 2017; 12(2): 101-9.
10. Bonhauser M, Fernandez G, Puschel K, et al. Improving physical fitness and emotional well-being in adolescents of low socioeconomic status in Chile: results of a school-based controlled trial. Health Promot Internation 2005; 20(2): 113-22.
11. Bouchard S, Gervais J, Gagnier N, Loranger C. Evaluation of a primary prevention program for anxiety disorders using story books with children aged 9-12 years. J Prim Prev 2013; 34(5): 345-58.
12. Britton Willoughby BLN, E. Niles Halsey, F. Rocha, Tomas Fisher Nathan, E. Gold Jonathan, S. A randomized controlled pilot trial of classroom-based mindfulness meditation compared to an active control condition in sixth-grade children. J Sch Psychol 2014; 52(3): 263-78.
13. Burckhardt R, Manicavasagar V, Batterham PJ, Miller LM, Talbot E, Lum A. A Web-Based Adolescent Positive Psychology Program in Schools: Randomized Controlled Trial. J Med Internet Res 2015; 17(7): e187.
14. Burckhardt R, Manicavasagar V, Batterham PJ, Hadzi-Pavlovic D. A randomized controlled trial of strong minds: A school-based mental health program combining acceptance and commitment therapy and positive psychology. J Sch Psychol 2016; 57: 41-52.
15. Calear AL, Christensen H, Mackinnon A, Griffiths KM, O'Kearney R. The YouthMood Project: a cluster randomized controlled trial of an online cognitive behavioral program with adolescents. J Consult Clin Psychol 2009; 77(6): 1021-32.
16. Calear AL, Batterham PJ, Poyser CT, Mackinnon AJ, Griffiths KM, Christensen H. Cluster randomised controlled trial of the e-couch Anxiety and Worry program in schools. J Affect Disord 2016; 196: 210-7.
17. Calear AL, Christensen H, Brewer J, Mackinnon A, Griffiths KM. A pilot randomized controlled trial of the e-couch anxiety and worry program in schools. Internet Interventions 2016; 6: 1-5.
18. Cardemil EV, Reivich KJ, Beevers CG, Seligman ME, James J. The prevention of depressive symptoms in low-income, minority children: two-year follow-up. Behav Res Ther 2007; 45(2): 313-27.
19. Chaplin TM, Gillham JE, Reivich K, et al. Depression Prevention for Early Adolescent Girls: A Pilot Study of All Girls Versus Co-Ed Groups. J Early Adolesc 2006; 26(1): 110-26.
20. Clarke Gregory NH, Wesley Murphy, Mary Sheeber, Lisa. School-based primary prevention of depressive symptomatology in adolescents: Findings from two studies. Journal of adolescent research 1993; 8(2): 183-204.
21. Clarke Gregory NH, Wesley Murphy, Mary Sheeber, Lisa. School-based primary prevention of depressive symptomatology in adolescents: Findings from two studies. Journal of adolescent research 1993; 8(2): 183-204.
22. Collins SWL, Marks Durkin, Kevin. Effects on coping skills and anxiety of a universal school-based mental health intervention delivered in Scottish primary schools. School Psychology International 2014; 35(1): 85-100.
23. Dadds M, H. Roth J. Prevention of Anxiety Disorders: Results of a Universal Trial with Young Children. J. Fam. Stud. 2008; 12 10.1007/s10826-007-9144-3
24. Eather NMP, J. Lubans David, R. Effects of exercise on mental health outcomes in adolescents: Findings from the CrossFitTM teens randomized controlled trial. Psychology of sport and exercise 2016; 26: 14-23.
25. Essau CA, Conradt J, Sasagawa S, Ollendick TH. Prevention of anxiety symptoms in children: results from a universal school-based trial. Behav 2012; 43(2): 450-64.

26. Gallegos, J. (2008). Preventing childhood anxiety and depression: Testing the effectiveness of a school-based program in méxico (Order No. 3341564). Available from ProQuest Dissertations & Theses Global. (304487266).
27. Gillham Jane E. Preventing depressive symptoms in school children. Dissertation abstracts international: section b: the sciences and engineering 1995; 55(9-B): 4119.
28. Gillham Jane ERK, J. Freres Derek, R. Lascher, Marisa Litzinger, Samantha Shatte, Andrew Seligman Martin, E. P. School-based prevention of depression and anxiety symptoms in early adolescence: A pilot of a parent intervention component. School psychology quarterly 2006; 21(3): 323-48.
29. Gillham JE, Reivich KJ, Freres DR, et al. School-based prevention of depressive symptoms: A randomized controlled study of the effectiveness and specificity of the Penn Resiliency Program. J Consult Clin Psychol 2007; 75(1): 9-19.
30. Gucht K, Griffith JW, Hellemans R, Bockstaele M, Pascal-Claes F, Raes F. Acceptance and Commitment Therapy (ACT) for adolescents: Outcomes of a large-sample, school-based, cluster-randomized controlled trial. Mindfulness 2017; 8(2): 408-16.
31. Haden SC, Daly L, Hagins M. A randomised controlled trial comparing the impact of yoga and physical education on the emotional and behavioural functioning of middle school children. Focus on alternative and complementary therapies 2014; 19(3): 148-55.
32. Hiebert BK, Boelle Jaknavorian, Armine. School-based relaxation: Attempting primary prevention. Canadian journal of counselling 1989; 23(3): 273-87.
33. Hodas R. An investigation of the relationship between positive and negative mental health factors and academic performance among early adolescent girls. Dissertation abstracts international: section b: the sciences and engineering 2016; 76(12-B(E)): No-Specified.
34. Horowitz JL, Garber J, Ciesla JA, Young JF, Mufson L. Prevention of depressive symptoms in adolescents: a randomized trial of cognitive-behavioral and interpersonal prevention programs. J Consult Clin Psychol 2007; 75(5): 693-706.
35. Johnson C, Burke C, Brinkman S, Wade T. Effectiveness of a school-based mindfulness program for transdiagnostic prevention in young adolescents. Behav Res Ther 2016; 81: 1-11.
36. Johnson C, Burke C, Brinkman S, Wade T. A randomized controlled evaluation of a secondary school mindfulness program for early adolescents: Do we have the recipe right yet? Behav Res Ther 2017; 99: 37-46.
37. Johnstone J, Rooney RM, Hassan S, Kane RT. Prevention of depression and anxiety symptoms in adolescents: 42 and 54 months follow-up of the Aussie Optimism Program-Positive Thinking Skills. Front Psychol 2014; 5: 364.
38. Khalsa SB, Hickey-Schultz L, Cohen D, Steiner N, Cope S. Evaluation of the mental health benefits of yoga in a secondary school: a preliminary randomized controlled trial. J Behav Health Serv Res 2012; 39(1): 80-90.
39. Kindt KC, Kleinjan M, Janssens JM, Scholte RH. Evaluation of a school-based depression prevention program among adolescents from low-income areas: a randomized controlled effectiveness trial. Int J Environ Res Public Health 2014; 11(5): 5273-93.
40. Lock S, Barrett PM. A longitudinal study of developmental differences in universal preventive intervention for child anxiety. Behaviour Change 2003; 20(4): 183-99.
41. Lowry-Webster Hayley MBP, M. Dadds Mark, R. A universal prevention trial of anxiety and depressive symptomatology in childhood: Preliminary data from an Australian study. Behaviour change 2001; 18(1): 36-50.
42. Mendelson T, Greenberg MT, Dariotis JK, Gould LF, Rhoades BL, Leaf PJ. Feasibility and preliminary outcomes of a school-based mindfulness intervention for urban youth. J Abnorm Child Psychol 2010; 38(7): 985-94.
43. Merry S, McDowell H, Wild CJ, Bir J, Cunliffe R. A randomized placebo-controlled trial of a school-based depression prevention program. J Am Acad Child Adolesc Psychiatry 2004; 43(5): 538-47.
44. Miller Lynn DS, Christina Garland, E. Jane Clark, Sandra. The ABCs of CBT (cognitive behavior therapy): Evidence-based approaches to child anxiety in public school settings. Journal of counseling and development 2010; 88(4): 432-9.
45. Miller LD, Laye-Gindhu A, Liu Y, March JS, Thordarson DS, Garland EJ. Evaluation of a preventive intervention for child anxiety in two randomized attention-control school trials. Behav Res Ther 2011; 49(5): 315-23.
46. Miller LD, Laye-Gindhu A, Liu Y, March JS, Thordarson DS, Garland EJ. Evaluation of a preventive intervention for child anxiety in two randomized attention-control school trials. Behav Res Ther 2011; 49(5): 315-23.
47. Pahl Kristine MBP, M. Preventing anxiety and promoting social and emotional strength in preschool children: A universal evaluation of the Fun FRIENDS program. Advances in school mental health promotion 2010; 3(3): 14-25.
48. Pattison C, Lynd-Stevenson R. The prevention of depressive symptoms in children: The immediate and long-term outcomes of a school-based program. Behaviour change 2001; 18(2): 92-102.
49. Perry Y, Werner-Seidler A, Calear A, et al. Preventing Depression in Final Year Secondary Students: School-Based Randomized Controlled Trial. J Med Internet Res 2017; 19(11): e369.
50. Pophillat E, Rooney RM, Nesa M, et al. Preventing Internalizing Problems in 6-8 Year Old Children: A Universal School-Based Program. Front Psychol 2016; 7: 1928.
51. Possel P, Horn AB, Groen G, Hautzinger M. School-based prevention of depressive symptoms in adolescents: a 6-month follow-up. J Am Acad Child Adolesc Psychiatry 2004; 43(8): 1003-10.

52. Possel P, Adelson JL, Hautzinger M. A randomized trial to evaluate the course of effects of a program to prevent adolescent depressive symptoms over 12 months. Behav Res Ther 2011; 49(12): 838-51.
53. Possel P, Martin NC, Garber J, Hautzinger M. A randomized controlled trial of a cognitive-behavioral program for the prevention of depression in adolescents compared with nonspecific and no-intervention control conditions. J Couns Psychol 2013; 60(3): 432-8.
54. Potek R. Mindfulness as a school-based prevention program and its effect on adolescent stress, anxiety and emotion regulation. Dissertation abstracts international: section b: the sciences and engineering 2012; 73(5-B): 3272.
55. Quayle DD, Suzanne Roberts, Claire Kane, Robert Ebsworthy, Greg. The effect of an optimism and lifeskills program on depressive symptoms in preadolescence. Behaviour change 2001; 18(4): 194-203.
56. Raes FGJ, W. Van der Gucht, Katleen Williams, J. Mark G. School-based prevention and reduction of depression in adolescents: A cluster-randomized controlled trial of a mindfulness group program. Mindfulness 2014; 5(5):477-86.
57. Reynolds EK, Macpherson L, Tull MT, Baruch DE, Lejuez CW. Integration of the brief behavioral activation treatment for depression (BATD) into a college orientation program: depression and alcohol outcomes. J Couns Psychol 2011; 58(4): 555-64.
58. Rivet-Duval EH, Sandra Hunt, Caroline. Preventing adolescent depression in Mauritius: A universal school-based program. Child and adolescent mental health 2011; 16(2): 86-91.
59. Roberts C, Kane R, Thomson H, Bishop B, Hart B. The prevention of depressive symptoms in rural school children: a randomized controlled trial. J Consult Clin Psychol 2003; 71(3): 622-8.
60. Roberts CM, Kane R, Bishop B, Cross D, Fenton J, Hart B. The prevention of anxiety and depression in children from disadvantaged schools. Behav Res Ther 2010; 48(1): 68-73.
61. Roberts CM, Kane RT, Rooney RM, et al. Efficacy of the Aussie Optimism Program: Promoting Pro-social Behavior and Preventing Suicidality in Primary School Students. A Randomised-Controlled Trial. Front Psychol 2018; 8(1392).
62. Rodgers AD, Sandra. A controlled evaluation of the 'FRIENDS for life' emotional resiliency programme on overall anxiety levels, anxiety subtype levels and school adjustment. Child and adolescent mental health 2015; 20(1): 13-9.
63. Rooney RR, Clare Kane, Robert Pike, Lisbeth Winsor, Amber White, Julia Brown, Annette. The Prevention of Depression in 8- to 9-Year-Old Children: A Pilot Study. Australian journal of guidance and counselling 2006; 16(1): 76-90.
64. Rose K, Hawes DJ, Hunt CJ. Randomized controlled trial of a friendship skills intervention on adolescent depressive symptoms. J Consult Clin Psychol 2014; 82(3): 510-20.
65. Ruttledge RD, Eileen Greene, Gabrielle Mullany, Mary Charles, Elizabeth Frehill, Joanne Moriarty, Maura. A randomised controlled trial of the FRIENDSfor Life emotional resilience programme delivered by teachers in Irish primary schools. Educational and child psychology 2016; 33(2): 69-89.
66. Sawyer MG, Pfeiffer S, Spence SH, et al. School-based prevention of depression: a randomised controlled study of the beyondblue schools research initiative. J Child Psychol Psychiatry 2010; 51(2): 199-209.
67. Shatte Andrew J. Prevention of depressive symptoms in adolescents: Issues of dissemination and mechanisms of change. Dissertation abstracts international: section b: the sciences and engineering 1997; 57(11-B): 7236.
68. Sheffield JK, Spence SH, Rapee RM, et al. Evaluation of universal, indicated, and combined cognitive-behavioral approaches to the prevention of depression among adolescents. J Consult Clin Psychol 2006; 74(1): 66-79.
69. Soffer, A. G. (2003). School -based social skills training to reduce children's depressive symptomatology (Order No. 3074683). Available from ProQuest Dissertations & Theses Global. (305330756).
70. Spence SH, Sheffield JK, Donovan CL. Preventing adolescent depression: An evaluation of the problem solving for life program. Journal of Consulting and Clinical Psychology 2003; 71(1): 3-13.
71. Stallard P, Phillips R, Montgomery AA, et al. A cluster randomised controlled trial to determine the clinical effectiveness and cost-effectiveness of classroom-based cognitive-behavioural therapy (CBT) in reducing symptoms of depression in high-risk adolescents. Health Technol Assess 2013; 17(47): vii-xvii, 1-109.
72. Stallard P, Skryabina E, Taylor G, et al. Classroom-based cognitive behaviour therapy (FRIENDS): a cluster randomised controlled trial to Prevent Anxiety in Children through Education in Schools (PACES). Lancet Psychiatry 2014; 1(3): 185-92.
73. Tak YR, Lichtwarck-Aschoff A, Gillham JE, Van Zundert RM, Engels RC. Universal School-Based Depression Prevention 'Op Volle Kracht': a Longitudinal Cluster Randomized Controlled Trial. J Abnorm Child Psychol 2016; 44(5): 949-61.
74. Tomba E, Belaise C, Ottolini F, et al. Differential effects of well-being promoting and anxiety-management strategies in a non-clinical school setting. J Anxiety Disord 2010; 24(3): 326-33.
75. Velasquez Ana MLM, Adelaida Quinonez, Natalia Paba Diana, Patricia. Yoga for the prevention of depression, anxiety, and aggression and the promotion of socio-emotional competencies in school-aged children. Educational research and evaluation 2015; 21(5-6): 407-21.
76. Wong N, Kady L, Mewton L, Sunderland M, Andrews G. Preventing anxiety and depression in adolescents: A randomised controlled trial of two school-based Internet-delivered cognitive behavioural therapy programmes. Internet interventions 2014; 1(2): 90-4.

77. Arnarson EO, Craighead WE. Prevention of depression among Icelandic adolescents. Behav Res Ther 2009; 47(7): 577-85.
78. Balle M, Tortella-Feliu M. Efficacy of a brief school-based program for selective prevention of childhood anxiety. Anxiety stress coping 2010; 23(1): 71-85.
79. Berry K, Hunt CJ. Evaluation of an intervention program for anxious adolescent boys who are bullied at school. J Adolesc Health 2009; 45(4): 376-82.
80. Clarke GN, Hawkins W, Murphy M, Sheeber LB, Lewinsohn PM, Seeley JR. Targeted prevention of unipolar depressive disorder in an at-risk sample of high school adolescents: a randomized trial of a group cognitive intervention. J Am Acad Child Adolesc Psychiatry 1995; 34(3): 312-21.
81. Congleton, Baker A. The effect of a cognitive-behavioral group intervention on the locus of control, attributional style, and depressive symptoms of middle school students; 2019. Thesis. University of Kentucky, USA.
82. Cooley-Strickland MR, Griffin RS, Darney D, Otte K, Ko J. Urban African American youth exposed to community violence: a school-based anxiety preventive intervention efficacy study. J 2011; 39(2): 149-66.
83. Cova FR, Paulina Melipillan, Roberto. Evaluation of the efficacy of a prevention program for depression in female adolescents. Terapia psicologica 2011; 29(2): 245-50.
84. Cowell JM, McNaughton D, Ailey S, Gross D, Fogg L. Clinical Trail Outcomes of the Mexican American Problem Solving Program (MAPS). Hispanic Health Care International : The Official Journal of The National Association of Hispanic Nurses 2009; 7(4): 179-89.
85. Cui L, He F, Han Z, Yang R, Xiao J, Oei TP. A brief group cognitive-behavioral program for the prevention of depressive symptoms in Chinese college students. International Journal of Group Psychotherapy 2016; 66(2): 291-307.
86. Dobson Keith SHJ, Ahnberg Fata, Ladan Scherrer, Martin Allan Lauren, C. The prevention of depression and anxiety in a sample of high-risk adolescents: A randomized controlled trial. Canadian journal of school psychology 2010; 25(4): 291-310.
87. Ellis Louise ACA, J. Sethi, Suvena O'Dea Bridianne, M. Comparative randomized trial of an online cognitive-behavioral therapy program and an online support group for depression and anxiety. Journal of cyber therapy and rehabilitation 2011; 4(4): 461-7.
88. Fitzgerald A, Rawdon C, Dooley B. A randomized controlled trial of attention bias modification training for socially anxious adolescents. Behav Res Ther 2016; 84: 1-8..
89. Fung J, Guo S, Jin J, Bear L, Lau A. A pilot randomized trial evaluating a school-based mindfulness intervention for ethnic minority youth. Mindfulness 2016; 7(4): 819-28.
90. Gaete J, Martinez V, Fritsch R, Rojas G, Montgomery AA, Araya R. Indicated school-based intervention to improve depressive symptoms among at risk Chilean adolescents: a randomized controlled trial. BMC Psychiatry 2016; 16: 276.
91. Gillham JE, Reivich KJ, Brunwasser SM, et al. Evaluation of a group cognitive-behavioral depression prevention program for young adolescents: a randomized effectiveness trial. J Clin Child Adolesc Psychol 2012; 41(5): 621-39.
92. Hiebert BK, Boelle Jaknavorian, Armine. School-based relaxation: Attempting primary prevention. Canadian journal of counselling 1989; 23(3): 273-87.
93. Higgins Diana M. Preventing generalized anxiety disorder in an at-risk sample of college students: A brief cognitive-behavioral approach. Dissertation abstracts international: section b: the sciences and engineering 2007; 67(9-B): 5406.
94. Hunt C, Andrews G, Crino R, Erskine A, Sakashita C. Randomized controlled trial of an early intervention programme for adolescent anxiety disorders. Aust N Z J Psychiatry 2009; 43(4): 300-4.
95. Jaycox LH, Reivich KJ, Gillham J, Seligman ME. Prevention of depressive symptoms in school children. Behav Res Ther 1994; 32(8): 801-16.
96. Jordans MJ, Komproe IH, Tol WA, et al. Evaluation of a classroom-based psychosocial intervention in conflict-affected Nepal: a cluster randomized controlled trial. J Child Psychol Psychiatry 2010; 51(7): 818-26.
97. Kiselica Mark SBS, B. Thomas Ronald, N. Reedy, Susan. Effects of stress inoculation training on anxiety, stress, and academic performance among adolescents. J Couns Psychol 1994; 41(3): 335-42.
98. Liddle I, Macmillan S. Evaluating the FRIENDS programme in a Scottish setting. Educational Psychology in Practice 2010; 26(1): 53-67.
99. Livheim FH, Louise Ghaderi, Ata Magnusdottir, Thora Hogfeldt, Anna Rowse, Julie Turner, Simone Hayes Steven, C. Tengstrom, Anders. The effectiveness of Acceptance and Commitment Therapy for adolescent mental health: Swedish and Australian pilot outcomes. Journal of child and family studies 2015; 24(4): 1016-30.
100. Manassis K, Wilansky-Traynor P, Farzan N, Kleiman V, Parker K, Sanford M. The feelings club: randomized controlled evaluation of school-based CBT for anxious or depressive symptoms. Depress Anxiety 2010; 27(10): 945-52.
101. McCarty CA, Violette HD, McCauley E. Feasibility of the positive thoughts and actions prevention program for middle schoolers at risk for depression. Depress Res Treat 2011; 2011: 241386.

102. McCarty CA, Violette HD, Duong MT, Cruz RA, McCauley E. A randomized trial of the Positive Thoughts and Action program for depression among early adolescents. J Clin Child Adolesc Psychol 2013; 42(4): 554-63.
103. McLaughlin C. Evaluating the effect of an empirically-supported group intervention for students at-risk for depression in a rural school district [thesis]. Dissertation abstracts international: section b: the sciences and engineering 2011; 71(9-b): 5820.
104. McLoone Jordana KRR, M. Comparison of an anxiety management program for children implemented at home and school: Lessons learned. Sch 2012; 4(4): 231-42.
105. Mifsud C, Rapee RM. Early intervention for childhood anxiety in a school setting: outcomes for an economically disadvantaged population. J Am Acad Child Adolesc Psychiatry 2005; 44(10): 996-1004.
106. Miller LD, Laye-Gindhu A, Bennett JL, et al. An effectiveness study of a culturally enriched school-based CBT anxiety prevention program. J Clin Child Adolesc Psychol 2011; 40(4): 618-29.
107. Noel La TR, Kathryn Gromer, Jill. Depression prevention among rural preadolescent girls: A randomized controlled trial. School social work journal 2013; 38(1): 1-18.
108. Owen HL, Wayne. The effects of three treatment methods upon anxiety and inappropriate attentional style among high school athletes. International journal of sport psychology 1982; 13(3): 154-62.
109. Peden AR, Hall LA, Rayens MK, Beebe LL. Reducing negative thinking and depressive symptoms in college women. J Nurs Scholarsh 2000; 32(2): 145-51.
110. Peng SQ, Aili Yuan, Fang. Experimental study on the effects of exercise prescription on the mental health of left-behind school children in rural areas. Revista argentina de clinica psicologica 2015; 24(3): 267-76.
111. Poppelaars M, Tak YR, Lichtwarck-Aschoff A, et al. A randomized controlled trial comparing two cognitive-behavioral programs for adolescent girls with subclinical depression: A school-based program (Op Volle Kracht) and a computerized program (SPARX). Behav Res Ther 2016; 80: 33-42.
112. Puskar K, Sereika S, Tusaie-Mumford K. Effect of the Teaching Kids to Cope (TKC) program on outcomes of depression and coping among rural adolescents. J Child Adolesc Psychiatr Nurs 2003; 16(2): 71-80.
113. Rice Cristy L. Reducing anxiety in middle school and high school students: A comparison of cognitive-behavioral therapy and relaxation training approaches. Dissertation abstracts international section a: humanities and social sciences 2009; 69(7-A): 2607.
114. Rohde P, Stice E, Shaw H, Briere FN. Indicated cognitive behavioral group depression prevention compared to bibliotherapy and brochure control: acute effects of an effectiveness trial with adolescents. J Consult Clin Psychol 2014; 82(1): 65-74.
115. Scholten H, Malmberg M, Lobel A, Engels RC, Granic I. A Randomized Controlled Trial to Test the Effectiveness of an Immersive 3D Video Game for Anxiety Prevention among Adolescents. PLoS ONE 2016; 11(1): e0147763.
116. Schoneveld EA, Malmberg M, Lichtwarck-Aschoff A, Verheijen GP, Engels RC, Granic I. A neurofeedback video game (MindLight) to prevent anxiety in children: A randomized controlled trial. Computers in Human Behavior 2016; 63: 321-33.
117. Schoneveld EA, Lichtwarck-Aschoff A, Granic I. Preventing Childhood Anxiety Disorders: Is an Applied Game as Effective as a Cognitive Behavioral Therapy-Based Program? Prev Sci 2018; 19(2): 220-32.
118. Seligman Martin EPS, Peter DeRubeis Robert, J. Hollon Steven, D. The prevention of depression and anxiety. Prevention & treatment 1999; 2(1): No-Specified.
119. Seligman ME, Schulman P, Tryon AM. Group prevention of depression and anxiety symptoms. Behav Res Ther 2007; 45(6): 1111-26.
120. Sheffield JK, Spence SH, Rapee RM, et al. Evaluation of universal, indicated, and combined cognitive-behavioral approaches to the prevention of depression among adolescents. J Consult Clin Psychol 2006; 74(1): 66-79.
121. Simpson Anna T. The roles of self-regulation and coping in a preventative cognitive-behavioural intervention for school-age children at-risk for internalizing disorders. Dissertation abstracts international: section b: the sciences and engineering 2008; 69(6-B): 3862.
122. Siu Fung Ying A. Internalizing problems among primary school children in Hong Kong: Prevalence and treatment. Dissertation abstracts international section a: humanities and social sciences 2008; 69(1-A): 115.
123. Sportel BE, de Hullu E, de Jong PJ, Nauta MH. Cognitive bias modification versus CBT in reducing adolescent social anxiety: a randomized controlled trial. PLoS ONE 2013; 8(5): e64355.
124. Stallard P, Phillips R, Montgomery AA, et al. A cluster randomised controlled trial to determine the clinical effectiveness and cost-effectiveness of classroom-based cognitive-behavioural therapy (CBT) in reducing symptoms of depression in high-risk adolescents. Health Technol Assess 2013; 17(47): vii-xvii, 1-109.
125. Stice E, Burton E, Bearman SK, Rohde P. Randomized trial of a brief depression prevention program: an elusive search for a psychosocial placebo control condition. Behav Res Ther 2007; 45(5): 863-76.
126. Stice E, Rohde P, Seeley JR, Gau JM. Brief cognitive-behavioral depression prevention program for high-risk adolescents outperforms two alternative interventions: a randomized efficacy trial. J Consult Clin Psychol 2008; 76(4): 595-606.
127. Stoppelbein Laura A. Primary prevention: An evaluation of a high-school based cognitive-behavioral program. Dissertation abstracts international: section b: the sciences and engineering 2004; 64(8-B): 4066.

128. Takagaki KO, Yasumasa Jinnin, Ran Mori, Asako Nishiyama, Yoshiko Yamamura, Takanao Yokoyama, Satoshi Shiota, Syouichi Okamoto, Yuri Miyake, Yoshie Ogata, Akiko Kunisato, Yoshihiko Shimoda, Haruki Kawakami, Norito Furukawa Toshi, A. Yamawaki, Shigeto. Behavioral activation for late adolescents with subthreshold depression: A randomized controlled trial. European child & adolescent psychiatry 2016: No-Specified.
129. Tokolahi E, Vandal AC, Kersten P, Pearson J, Hocking C. Cluster-randomised controlled trial of an occupational therapy intervention for children aged 11–13 years, designed to increase participation to prevent symptoms of mental illness. Child and Adolescent Mental Health 2018; 23(4): 313-27..
130. Topper M, Emmelkamp PM, Watkins E, Ehring T. Prevention of anxiety disorders and depression by targeting excessive worry and rumination in adolescents and young adults: A randomized controlled trial. Behav Res Ther 2017; 90: 123-36.
131. van Starrenburg ML, Kuijpers RC, Kleinjan M, Hutschemaekers GJ, Engels RC. Effectiveness of a Cognitive Behavioral Therapy-Based Indicated Prevention Program for Children with Elevated Anxiety Levels: a Randomized Controlled Trial. Prev Sci 2017; 18(1): 31-9.
132. Wijnhoven LA, Creemers DH, Vermulst AA, Scholte RH, Engels RC. Randomized controlled trial testing the effectiveness of a depression prevention program ('Op Volle Kracht') among adolescent girls with elevated depressive symptoms. J Abnorm Child Psychol 2014; 42(2): 217-28.
133. Woods BJP, E. Effectiveness of a school-based indicated early intervention program for Maori and Pacific adolescents. Journal of pacific rim psychology 2011; 5(1): 40-50.
134. Young JF, Mufson L, Davies M. Efficacy of Interpersonal Psychotherapy-Adolescent Skills Training: an indicated preventive intervention for depression. J Child Psychol Psychiatry 2006; 47(12): 1254-62.
135. Young JF, Mufson L, Gallop R. Preventing depression: a randomized trial of interpersonal psychotherapy-adolescent skills training. Depress Anxiety 2010; 27(5): 426-33.
136. Young JF, Benas JS, Schueler CM, Gallop R, Gillham JE, Mufson L. A Randomized Depression Prevention Trial Comparing Interpersonal Psychotherapy--Adolescent Skills Training to Group Counseling in Schools. Prev Sci 2016; 17(3): 314-24.
137. Yu L. Preventing depressive symptoms in Chinese children. Dissertation abstracts international: section b: the sciences and engineering 2000; 60(12-B): 6389.

Complete results of network meta-analysis by population, setting and outcome: post-intervention

SMD: standardised mean difference. Lower CrI: lower credible interval and Upper CrI: upper credible interval from a 95% Credible Interval

#Direct trials: number of head to head trials available for that comparison

Negative values favour intervention in “active intervention” column.

Universal Secondary Depression

Reference	Active intervention	#Direct trials	SMD	Lower CrI	Upper CrI
Usual curriculum	Wait list	0	0.00	-0.19	0.19
Usual curriculum	No intervention	0	0.03	-0.15	0.21
Usual curriculum	Attention control	1	0.07	-0.12	0.25
Usual curriculum	CBT	11	-0.04	-0.16	0.07
Usual curriculum	Third wave	1	-0.03	-0.21	0.14
Usual curriculum	IPT+CBT	0	-0.19	-0.46	0.08
Usual curriculum	IPT	1	-0.03	-0.36	0.29
Usual curriculum	Psychoeducation	1	-0.13	-0.49	0.22
Usual curriculum	Behaviour therapy	1	-0.02	-0.40	0.37
Wait list	No intervention	0	0.03	-0.18	0.24
Wait list	Attention control	0	0.07	-0.14	0.27
Wait list	CBT	5	-0.04	-0.20	0.11
Wait list	Third wave	0	-0.04	-0.29	0.22
Wait list	IPT+CBT	2	-0.19	-0.41	0.04
Wait list	IPT	0	-0.03	-0.39	0.33
Wait list	Psychoeducation	0	-0.14	-0.53	0.27
Wait list	Behaviour therapy	0	-0.02	-0.44	0.41
No intervention	Attention control	2	0.04	-0.16	0.22
No intervention	CBT	7	-0.07	-0.21	0.07
No intervention	Third wave	0	-0.06	-0.32	0.18
No intervention	IPT+CBT	0	-0.22	-0.50	0.06
No intervention	IPT	0	-0.06	-0.41	0.29
No intervention	Psychoeducation	0	-0.16	-0.56	0.23
No intervention	Behaviour therapy	0	-0.05	-0.47	0.38
Attention control	CBT	2	-0.11	-0.27	0.05
Attention control	Third wave	0	-0.10	-0.35	0.15
Attention control	IPT+CBT	1	-0.25	-0.51	0.00
Attention control	IPT	0	-0.10	-0.45	0.26
Attention control	Psychoeducation	0	-0.20	-0.60	0.20
Attention control	Behaviour therapy	0	-0.08	-0.51	0.34
CBT	Third wave	0	0.01	-0.20	0.21
CBT	IPT+CBT	0	-0.15	-0.39	0.10
CBT	IPT	0	0.01	-0.31	0.33
CBT	Psychoeducation	0	-0.09	-0.46	0.28
CBT	Behaviour therapy	0	0.02	-0.37	0.43
Third wave	IPT+CBT	0	-0.15	-0.47	0.17
Third wave	IPT	0	0.00	-0.36	0.37
Third wave	Psychoeducation	0	-0.10	-0.49	0.29
Third wave	Behaviour therapy	0	0.02	-0.40	0.44
IPT+CBT	IPT	0	0.15	-0.25	0.56
IPT+CBT	Psychoeducation	0	0.05	-0.39	0.49

IPT+CBT	Behaviour therapy	0	0.17	-0.30	0.64
IPT	Psychoeducation	0	-0.10	-0.58	0.37
IPT	Behaviour therapy	0	0.02	-0.49	0.51
Psychoeducation	Behaviour therapy	0	0.12	-0.40	0.64

Universal Secondary Anxiety

Reference	Active intervention	Direct trials #	SMD	Lower CrI	Upper CrI
Usual curriculum	Wait list	0	-0.05	-0.28	0.18
Usual curriculum	No intervention	0	-0.07	-0.34	0.20
Usual curriculum	Attention control	0	-0.15	-0.51	0.16
Usual curriculum	CBT	3	-0.15	-0.34	0.04
Usual curriculum	Third wave	3	0.03	-0.14	0.20
Usual curriculum	Mindfulness/Relaxation	0	-0.65	-1.14	-0.19
Wait list	No intervention	0	-0.02	-0.25	0.21
Wait list	Attention control	0	-0.10	-0.43	0.17
Wait list	CBT	6	-0.10	-0.24	0.03
Wait list	Third wave	0	0.08	-0.21	0.37
Wait list	Mindfulness/Relaxation	1	-0.60	-1.06	-0.17
No intervention	Attention control	0	-0.08	-0.44	0.23
No intervention	CBT	4	-0.07	-0.27	0.11
No intervention	Third wave	0	0.10	-0.22	0.42
No intervention	Mindfulness/Relaxation	0	-0.58	-1.07	-0.11
Attention control	CBT	1	0.01	-0.25	0.30
Attention control	Third wave	0	0.18	-0.17	0.59
Attention control	Mindfulness/Relaxation	1	-0.50	-0.90	-0.10
CBT	Third wave	0	0.18	-0.07	0.44
CBT	Mindfulness/Relaxation	0	-0.50	-0.95	-0.08
Third wave CBT	Mindfulness/Relaxation	0	-0.68	-1.21	-0.19

Universal Primary Depression

Reference	Active intervention	Direct trials #	SMD	Lower CrI	Upper CrI
Usual curriculum	Wait list	0	-0.09	-0.77	0.54
Usual curriculum	No Intervention	0	0.13	-0.40	0.65
Usual curriculum	Attention control	0	-0.07	-0.79	0.62
Usual curriculum	CBT	6	-0.13	-0.44	0.17
Usual curriculum	BT	0	-0.10	-1.04	0.80
Wait list	No Intervention	0	0.22	-0.48	0.96
Wait list	Attention control	0	0.02	-0.83	0.88
Wait list	CBT	2	-0.04	-0.60	0.56
Wait list	BT	0	-0.01	-1.05	1.04
No Intervention	Attention control	2	-0.20	-0.79	0.37
No Intervention	CBT	3	-0.26	-0.69	0.17

No Intervention	BT	1	-0.23	-1.03	0.55
Attention control	CBT	0	-0.06	-0.68	0.59
Attention control	BT	0	-0.03	-0.84	0.78
CBT	BT	0	0.03	-0.85	0.88

Universal Primary Anxiety

Reference	Active intervention	Direct trials #	SMD	Lower CrI	Upper CrI
Usual curriculum	Wait list	0	0.02	-0.20	0.22
Usual curriculum	No Intervention	0	0.23	-0.15	0.60
Usual curriculum	Attention control	0	-0.17	-0.51	0.17
Usual curriculum	CBT	6	-0.07	-0.23	0.05
Wait list	No Intervention	0	0.20	-0.18	0.58
Wait list	Attention control	0	-0.19	-0.54	0.16
Wait list	CBT	5	-0.10	-0.26	0.06
No Intervention	Attention control	1	-0.39	-0.83	0.04
No Intervention	CBT	2	-0.30	-0.65	0.05
Attention control	CBT	2	0.09	-0.22	0.40

Targeted Secondary Depression

Reference	Active intervention	Direct trials #	SMD	Lower CrI	Upper CrI
No intervention	Wait list	0	0.22	-0.28	0.70
No intervention	Usual curriculum	0	0.04	-0.72	0.81
No intervention	Attention control	0	-0.81	-1.82	0.18
No intervention	Psycho-supprt	0	0.02	-0.63	0.66
No intervention	CBT	5	-0.22	-0.58	0.13
No intervention	Third wave	0	-3.74	-4.90	-2.59
No intervention	IPT	0	-0.65	-1.50	0.16
No intervention	Bias modification	0	-0.90	-2.21	0.40
No intervention	Exercise	1	-0.28	-1.13	0.58
No intervention	Psychoeducation	0	0.12	-0.50	0.72
Wait list	Usual curriculum	0	-0.18	-0.92	0.59
Wait list	Attention control	0	-1.03	-2.02	-0.04
Wait list	Psycho-support	0	-0.20	-0.82	0.43
Wait list	CBT	7	-0.44	-0.77	-0.10
Wait list	Third wave	0	-3.96	-5.11	-2.81
Wait list	IPT	0	-0.87	-1.70	-0.06
Wait list	Bias modification	0	-1.12	-2.41	0.18
Wait list	Exercise	0	-0.50	-1.47	0.49
Wait list	Psychoeducation	0	-0.10	-0.70	0.49
Usual curriculum	Attention control	0	-0.85	-2.02	0.29

Usual curriculum	Psycho-support	0	-0.02	-0.90	0.83
Usual curriculum	CBT	2	-0.26	-0.95	0.41
Usual curriculum	Third wave	0	-3.78	-5.08	-2.50
Usual curriculum	IPT	0	-0.69	-1.73	0.30
Usual curriculum	Bias modification	0	-0.94	-2.38	0.48
Usual curriculum	Exercise	0	-0.32	-1.47	0.82
Usual curriculum	Psychoeducation	0	0.08	-0.78	0.90
Attention control	Psycho-supprt	0	0.83	-0.24	1.91
Attention control	CBT	1	0.59	-0.34	1.53
Attention control	Third wave	0	-2.93	-4.37	-1.49
Attention control	IPT	0	0.16	-1.04	1.35
Attention control	Bias modification	1	-0.09	-0.92	0.75
Attention control	Exercise	0	0.53	-0.77	1.86
Attention control	Psychoeducation	0	0.93	-0.13	1.98
Psycho-support	CBT	1	-0.24	-0.78	0.30
Psycho-support	Third wave	1	-3.76	-4.72	-2.80
Psycho-support	IPT	3	-0.67	-1.21	-0.16
Psycho-support	Bias modification	0	-0.92	-2.29	0.44
Psycho-support	Exercise	0	-0.30	-1.36	0.77
Psycho-support	Psychoeducation	1	0.09	-0.57	0.75
CBT	Third wave	0	-3.52	-4.62	-2.42
CBT	IPT	0	-0.43	-1.19	0.31
CBT	Bias modification	0	-0.68	-1.93	0.57
CBT	Exercise	0	-0.06	-0.97	0.87
CBT	Psychoeducation	1	0.33	-0.16	0.83
Third wave	IPT	0	3.09	1.99	4.17
Third wave	Bias modification	0	2.84	1.17	4.51
Third wave	Exercise	0	3.46	2.03	4.90
Third wave	Psychoeducation	0	3.85	2.69	5.02
IPT	Bias modification	0	-0.25	-1.70	1.22
IPT	Exercise	0	0.37	-0.80	1.58
IPT	Psychoeducation	0	0.76	-0.06	1.62
Bias modification	Exercise	0	0.62	-0.93	2.19
Bias modification	Psychoeducation	0	1.01	-0.33	2.36
Exercise	Psychoeducation	0	0.39	-0.66	1.43

Targeted Secondary Anxiety

**Reference**	**Active intervention**	**Direct trials #**	**SMD**	**Lower CrI**	**Upper CrI**
No intervention	Wait list	0	0.30	0.09	0.53
No intervention	Attention control	0	-0.09	-0.39	0.22
No intervention	Psycho-support	0	1.08	0.52	1.64
No intervention	CBT	4	0.03	-0.11	0.16
No intervention	Biofeedback	0	-0.18	-0.55	0.21

No intervention	Mindfulness/Relaxation	0	0.03	-0.42	0.48
No intervention	Bias modification	1	-0.17	-0.45	0.11
No intervention	Exercise	1	-0.47	-0.86	-0.09
Wait list	Attention control	0	-0.40	-0.71	-0.09
Wait list	Psycho-support	0	0.77	0.20	1.34
Wait list	CBT	4	-0.28	-0.45	-0.11
Wait list	Biofeedback	1	-0.48	-0.86	-0.11
Wait list	Mindfulness/Relaxation	1	-0.28	-0.71	0.15
Wait list	Bias modification	0	-0.48	-0.79	-0.17
Wait list	Exercise	0	-0.77	-1.22	-0.34
Attention control	Psycho-support	0	1.17	0.56	1.78
Attention control	CBT	2	0.12	-0.17	0.40
Attention control	Biofeedback	1	-0.08	-0.38	0.20
Attention control	Mindfulness/Relaxation	1	0.12	-0.31	0.55
Attention control	Bias modification	1	-0.08	-0.36	0.20
Attention control	Exercise	0	-0.38	-0.87	0.11
Psycho-support	CBT	1	-1.05	-1.60	-0.50
Psycho-support	Biofeedback	0	-1.25	-1.91	-0.60
Psycho-support	Mindfulness/Relaxation	0	-1.05	-1.75	-0.36
Psycho-support	Bias modification	0	-1.25	-1.86	-0.64
Psycho-support	Exercise	0	-1.55	-2.23	-0.87
CBT	Biofeedback	0	-0.20	-0.56	0.16
CBT	Mindfulness/Relaxation	0	0.00	-0.43	0.43
CBT	Bias modification	0	-0.20	-0.46	0.07
CBT	Exercise	0	-0.50	-0.91	-0.09
Biofeedback	Mindfulness/Relaxation	0	0.20	-0.24	0.65
Biofeedback	Bias modification	0	0.00	-0.37	0.38
Biofeedback	Exercise	0	-0.30	-0.84	0.25
Mindfulness/Relaxation	Bias modification	0	-0.20	-0.67	0.27
Mindfulness/Relaxation	Exercise	0	-0.50	-1.09	0.09
Bias modification	Exercise	0	-0.30	-0.78	0.17

Targeted Primary Depression

**Reference**	**Active intervention**	**Direct trials #**	**SMD**	**Lower CrI**	**Upper CrI**
Wait list	Attention control	0	-0.72	-3.56	2.10
Wait list	CBT	2	-0.48	-2.49	1.50
Wait list	OT	1	-0.10	-2.94	2.71
Attention control	CBT	2	0.25	-1.76	2.21
Attention control	OT	0	0.62	-3.39	4.60

CBT	OT	0	0.38	-3.06	3.84

Targeted Primary Anxiety

**Reference**	**Active intervention**	**Direct trials #**	**SMD**	**Lower CrI**	**Upper CrI**
Wait list	Attention control	0	-0.35	-1.05	0.33
Wait list	CBT	5	-0.38	-0.84	0.07
Wait list	OT	1	0.11	-0.91	1.14
Wait list	Biofeedback	0	-0.38	-1.50	0.72
Attention control	CBT	4	-0.03	-0.54	0.49
Attention control	OT	0	0.47	-0.77	1.71
Attention control	Biofeedback	0	-0.03	-1.16	1.11
CBT	OT	0	0.50	-0.62	1.62
CBT	Biofeedback	1	0.00	-1.01	1.01
OT	Biofeedback	0	-0.50	-2.01	1.01
